# Incorporating a Piperidinyl Group in the Fluorophore Extends the Fluorescence Lifetime of Click-Derived Cyclam-Naphthalimide Conjugates

**DOI:** 10.1371/journal.pone.0100761

**Published:** 2014-07-01

**Authors:** Mingfeng Yu, Sandra Ast, Qun Yu, Anthony T. S. Lo, Roman Flehr, Matthew H. Todd, Peter J. Rutledge

**Affiliations:** 1 School of Chemistry, The University of Sydney, Sydney, New South Wales, Australia; 2 Institute for Chemistry, University of Potsdam, Potsdam, Brandenburg, Germany; University of East Anglia, United Kingdom

## Abstract

Ligands incorporating a tetraazamacrocycle receptor, a ‘click’- derived triazole and a 1,8-naphthalimide fluorophore have proven utility as probes for metal ions. Three new cyclam-based molecular probes are reported, in which a piperidinyl group has been introduced at the 4-position of the naphthalimide fluorophore. These compounds have been synthesized using the copper(I)-catalyzed azide-alkyne Huisgen cycloaddition and their photophysical properties studied in detail. The alkylamino group induces the expected red-shift in absorption and emission spectra relative to the simple naphthalimide derivatives and gives rise to extended fluorescence lifetimes in aqueous buffer. The photophysical properties of these systems are shown to be highly solvent-dependent. Screening the fluorescence responses of the new conjugates to a wide variety of metal ions reveals significant and selective fluorescence quenching in the presence of copper(II), yet no fluorescence enhancement with zinc(II) as observed previously for the simple naphthalimide derivatives. Reasons for this different behaviour are proposed. Cytotoxicity testing shows that these new cyclam-triazole-dye conjugates display little or no toxicity against either DLD-1 colon carcinoma cells or MDA-MB-231 breast carcinoma cells, suggesting a potential role for these and related systems in biological sensing applications.

## Introduction

Given the various essential roles played by metal ions in biological systems and environmental processes, the development of fluorescent probes with high selectivity and sensitivity for these species is of great importance. [1]–[8] Due to their tunable photophysical properties and ease of preparation, 1,8-naphthalimide derivatives are commonly used as fluorophores in metal ion probes. [9]–[22] Structural modifications of the 1,8-naphthalimide are readily accommodated on either the aromatic naphthalene moiety or the imide-NH site.

**Figure 1:**
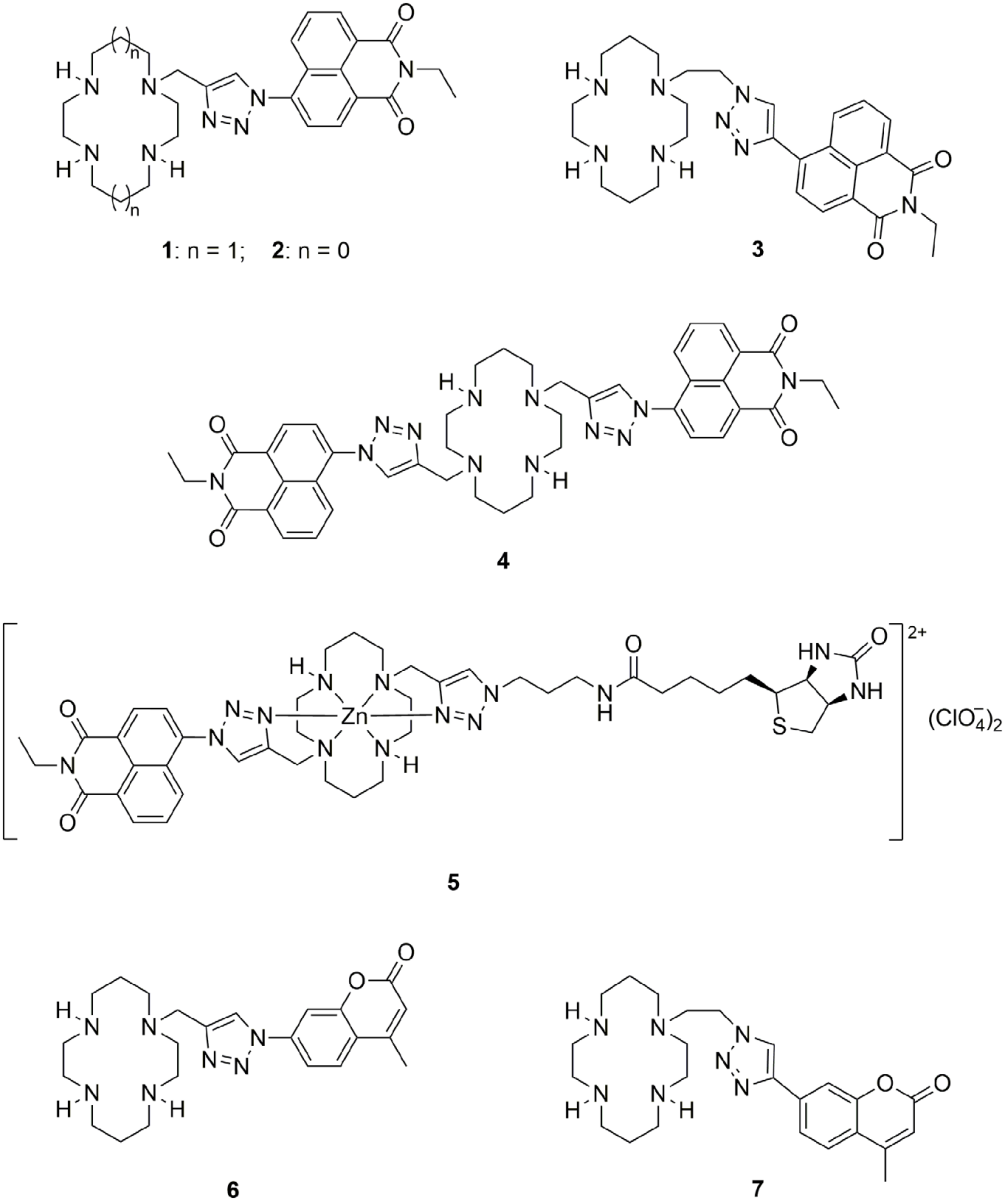
Fluorescent probes 1–7 used in previous studies.

We have recently developed a novel class of fluorescent probes for Zn^2+^ (Figure 1) by attaching the 1,8-naphthalimide fluorophore to a tetraazamacrocycle scaffold *via* copper(I)-catalyzed azide-alkyne Huisgen cycloaddition (colloquially known as the click reaction). [19]–[22] The click-generated triazole is a linker but also acts as a coordination site, thus playing a role in the metal ion binding and detection. Compounds **1**–**4** signal the binding of Zn^2+^ to the tetraazamacrocycle-triazole moiety with a multifold increase in fluorescence emission of the pendant 1,8-naphthalimide. Reversing the triazole topology in the cyclam-triazole-naphthalimide system (**3** *vs.* **1**) gives a 10-fold brighter fluorescence response to Zn^2+^ in HEPES buffer (10 mM, pH 7.4). [22] Furthermore, the cyclam-based probe **1** has been used to detect the cellular Zn^2+^ flux during apoptosis *in vitro*, [21] and the cyclen-based probe **2** has been applied *in vivo* to image Zn^2+^ in zebrafish. [19] In a related approach, tethering a second pendant group (biotin) to the zinc(II) complex of compound **1** afforded a fluorescent ‘allosteric scorpionand’ probe **5** that visualizes the binding of the pendant biotin to the cognate biomolecule avidin. [23] Replacement of the 1,8-naphthalimide dye in compounds **1** and **3** with the coumarin fluorophore provided probes **6** and **7** (Figure 1) that respond selectively to Cu^2+^ and Hg^2+^ [22], [24].

**Figure 2:**
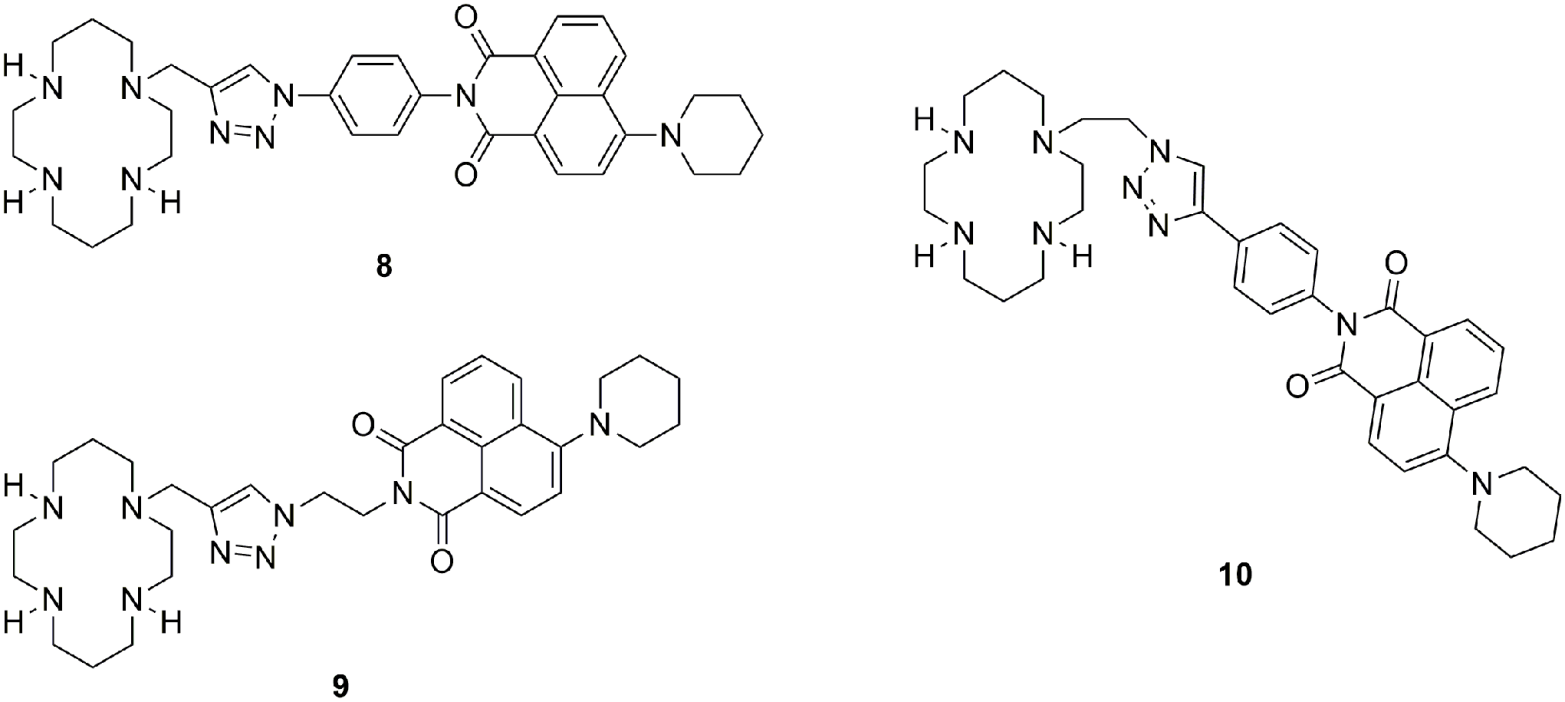
Cyclam-piperidinylnaphthalimide conjugates 8–10 studied in this work.

To minimize cell damage and interference from background autofluorescence in cell-based assays, the absorption and emission spectra of the fluorescent probe should be as close as possible to the red end of the visible spectrum. [25] In this regard the spectral characteristics of probes **1**–**7** are sub-optimal (λ_abs_∼320–360 nm, λ_em_∼380–460 nm in aqueous buffer). [19]–[24] Previous studies have shown that introducing alkylamino groups at the naphthalene moiety of 1,8-naphthalimide induces such a bathochromic shift. [11], [26]–[29] To this end, we designed three new cyclam-piperidinylnaphthalimide conjugates **8**–**10** (Figure 2). A phenyl linker was used in compounds **8** and **10** to connect the cyclam-triazole moiety to the piperidinylnaphthalimide fluorophore, while compound **9**, containing a flexible ethylene chain, was designed as a control to verify the importance of conjugation. The metal-ion responsiveness, fluorescence quantum yields and decay times, and cytotoxicity of these new conjugates were investigated to explore their potential for application as metal ion probes *in vitro* and *in vivo*.

## Results and Discussion

### (a) Synthesis

**Figure 3:**
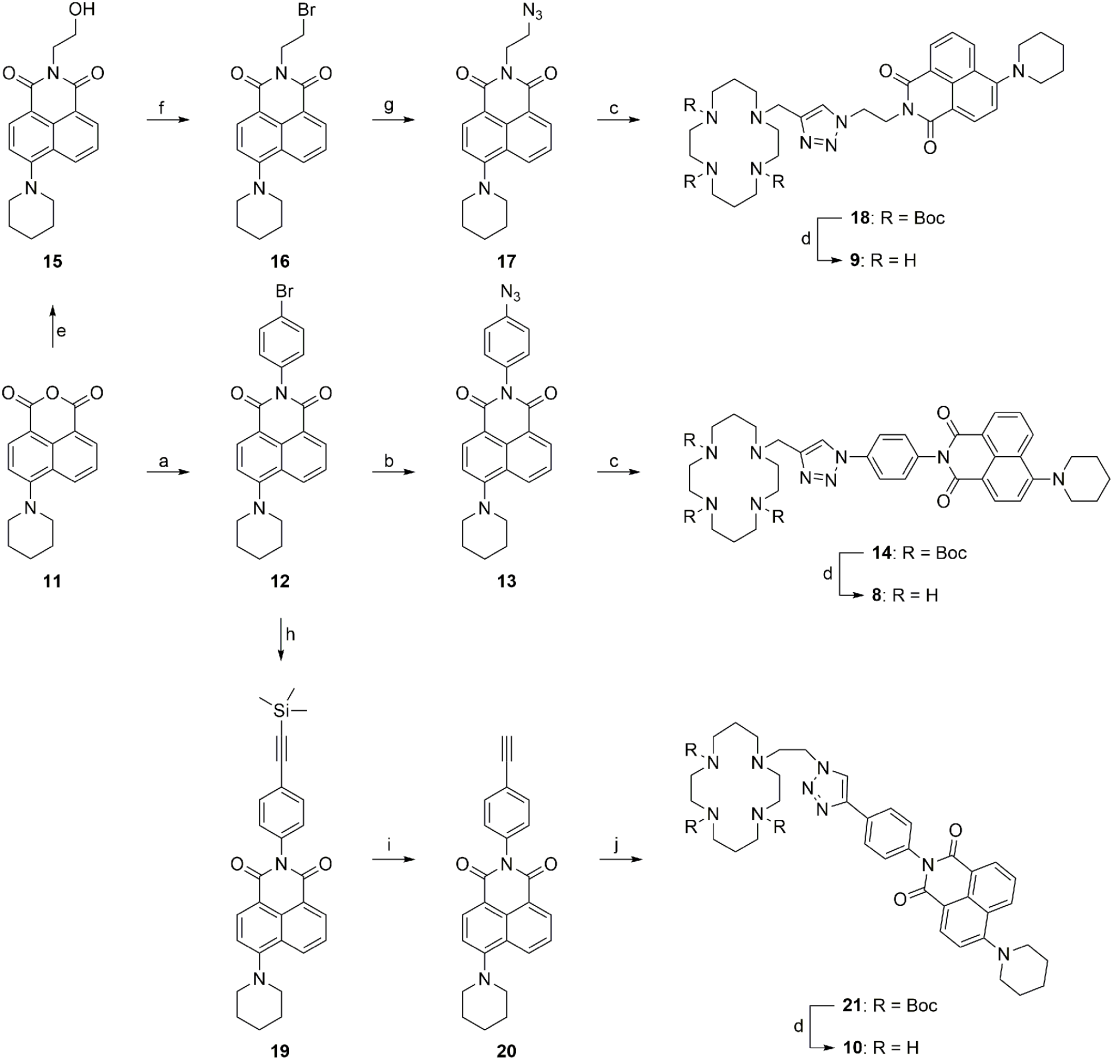
Synthesis of the cyclam-piperidinylnaphthalimide conjugates 8–10. *Reagents and conditions*: (a) 4-bromoaniline, piperidine, 2-methoxyethanol, reflux, 72 h, 90%; (b) NaN_3_, CuI, sodium ascorbate, DMEDA, THF/H_2_O (7∶3), 12 h, 50%; (c) propargyl-tri-Boc cyclam, CuSO_4_·5H_2_O, sodium ascorbate, THF/H_2_O (7∶3), rt for **13** and 50°C for **17**, 12 h, **14**: 96%, **18**: 92%; (d) (i) TFA/DCM/H_2_O (90∶5∶5), rt, 6 h; (ii) Ambersep 900 hydroxide form, CH_3_OH, rt, 15 min, **8**: 96%, **9**: 99%, **10**: 99%; (e) 2-aminoethanol, EtOH, reflux, 22 h, 92%; (f) PBr_3_, pyridine, THF, 50°C, 16 h, 60%; (g) NaN_3_, EtOH, reflux, 6 h, 80%; (h) trimethylsilylacetylene, CuI, triphenylphosphine, Pd(PPh_3_)_4_, Et_3_N, pyridine, 85°C, o/n, 94%; (i) K_2_CO_3_, CH_3_OH, rt, o/n, 97%; (j) 2-azidoethyl-tri-Boc cyclam, CuSO_4_·5H_2_O, sodium ascorbate, THF/H_2_O (7∶3), 12 h, 66%.

Synthesis of the cyclam-piperidinylnaphthalimide conjugates **8**–**10** required the preparation of precursors **13**, **17** and **20** (Figure 3). Azide **17** [30], [31] and alkyne **20** [29], [30], [32] were successfully synthesized according to literature procedures, whereas the preparation of azide **13** proved challenging. Conversion of bromide **12** to the corresponding azide **13** was initially attempted with sodium azide in the presence of sodium ascorbate, copper(I) iodide and *N*, *N*′-dimethylethylenediamine (DMEDA) at reflux in either an ordinary round-bottomed flask or a pressure tube. [33]–[35] A solvent screen including methanol/water, ethanol/water or dimethyl sulfoxide (DMSO)/water (7∶3 in all cases) failed to afford the desired azide **13**, giving instead full recovery of starting material **12**; this outcome may be attributed to the extraordinarily low solubility of bromide **12** in these solvent combinations. Switching to tetrahydrofuran (THF)/water (7∶3), all reactants and reagents were dissolved at reflux in the pressure tube and reaction proceeded to give azide **13** in 50% yield. The corresponding amine was also detected by LCMS analysis of the reaction mixture, consistent with previous observations that both azide and amine may be generated through a copper-assisted aromatic substitution reaction with sodium azide. [33] Reacting each of the three precursors **13**, **17** and **20** individually with the complementary propargyl-tri-Boc cyclam [23], [36] or azidoethyl-tri-Boc cyclam [24] under standard click conditions [24], [36] yielded the Boc-protected cyclam-piperidinylnaphthalimide conjugates **14**, **18** and **21** respectively in good to excellent yields. Removal of Boc groups from these conjugates was effected in a mixture of TFA/DCM/H_2_O (90∶5∶5), [23], [36], [37] followed by basification to recover the corresponding free amines **8**–**10**. However, the outcome of the basification step was contingent on the base used. Addition of 2 M sodium hydroxide solution [36], [38] or saturated sodium carbonate solution [36] resulted in decomposition of the desired free amines or incomplete removal of trifluoroacetate counter ions respectively (indicated by analysis with ^1^H and ^13^C NMR spectroscopy). Successful isolation of the pure amines **8**–**10** was achieved using excess Ambersep 900 (hydroxide form) in methanol.

### (b) Photophysical Properties

#### i) Steady-state photophysical properties

**Figure 4:**
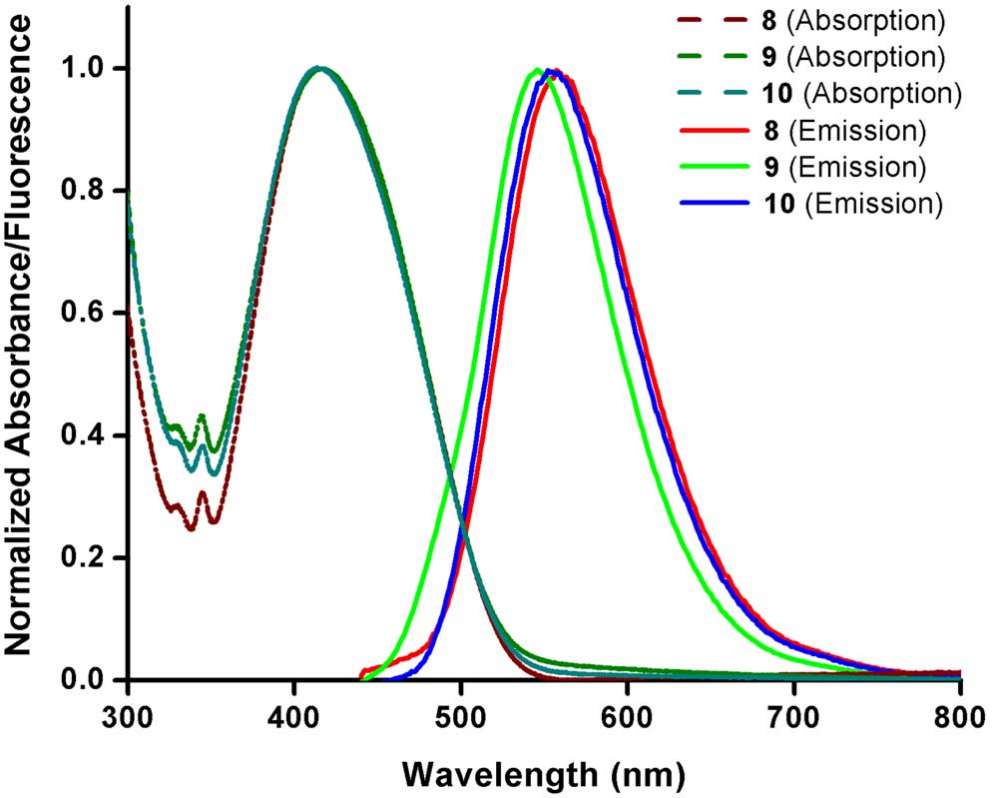
Normalized UV-Vis and fluorescence spectra of 8–10. Experiments were carried out in HEPES buffer (10 mM, pH 7.4) at 25°C.

The steady state photophysical properties of cyclam-piperidinylnaphthalimide conjugates **8**–**10** were investigated using both UV-Vis and fluorescence spectroscopy. The UV-Vis absorption spectra of **8**–**10** in HEPES buffer (10 mM, pH 7.4) are almost identical, with the lowest-energy absorption (λ_abs_) centered at 415±2 nm and stretching out to 500 nm (Figure 4). The fluorescence emission spectra of **8**–**10** are only slightly shifted giving a broad emission band ranging from 500 to 700 nm, centered around 545–558 nm (λ_em_) (Figure 4). Introduction of the piperidine to the naphthalimide fluorophore not only leads to a red-shifted emission maximum but also to a broadening of both the absorption and emission bands. The similarity of these spectra in aqueous buffer is remarkable, and implies i) the role of the linker (phenyl **8** *vs* ethyl **9**) exerts minimal influence and ii) the triazole connectivity (**8** *vs* **10**) does not have a significant impact on the UV-Vis absorption and fluorescence emission of these conjugates. The fact that the π-system is not extended in **8** or **10** by conjugation of the phenyl group with the 1,8-naphthalimide core can be rationalized by considering a twisting of the two aromatic planes to minimize adverse steric interactions. This effect may be enhanced after excitation of the probe, giving rise to charge separated states and significant solvent-dependent variation in spectral properties.

Screening the spectral properties of **8**–**10** in various solvents spanning a wide range of polarities revealed a solvent-dependent shift in absorption, and – to a much larger extent – emission maxima (Table 1). Comparing measurements made in aqueous buffer versus non-polar toluene shows that the impact of solvent polarity is less in the case of ethyl-linked **9**, where the emission in HEPES buffer (545 nm) shifts less than 40 nm in toluene (507 nm). In the emissions of **8** and **10**, a blue-shift of nearly 60 nm is seen in toluene relative to HEPES buffer. The Stokes shifts (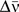) (calculated from the difference of the absorption and emission maxima) allow easier comparison: the Stokes shifts of ligands **8** and **10** respond similarly throughout the solvent screen; the slight differences that are observed between the two ligands can be attributed to the effect of the different triazole connectivity (further evidence for the minor impact this structural change exerts on the spectral properties). More importantly, there is a distinct decrease in the Stokes shift of both compounds when moving from HEPES buffer into less polar solvents, suggesting that charge separation in the excited state is most likely linked to conformational changes. In the ethyl-linked analogue **9**, the effect of the solvent is weaker, indicating that the excited state of **9** incorporates a much smaller charge separation. Lippert-Mataga plots [39]–[41] (Figures S1–S3 and Text S1 in File S1) were constructed to build a picture of solvent-fluorophore interactions. The Stokes shifts of all three conjugates in the hydrogen bonding solvents (*e.g.* alcohols) are typically greater than those in solvents that less readily form hydrogen bonds (*e.g.* toluene); such behavior can be attributed to protic solvent-fluorophore hydrogen bonding and has been observed for other fluorophores [42], [43].

**Table 1:** Photophysical properties of 8–10 in various solvents with decreasing polarity from aqueous (HEPES buffer) to toluene.

	λ_abs_/nm			λ_em_/nm			/cm^−1^		
Solvent	8	9	10	8	9	10	8	9	10
**HEPES**	415	417	414	558	545	555	6175	5632	6137
**MeOH**	415	7	415	542	538	542	5646	5393	5646
**EtOH**	413	415	412	540	536	539	5695	5440	5719
**n-PrOH**	413	413	412	537	534	536	5591	5486	5615
**n-BuOH**	413	411	411	536	534	534	5556	5604	5604
**DMSO**	415	416	413	540	536	540	5578	5382	5695
**MeCN**	411	412	409	539	537	538	5778	5650	5863
**DMF**	410	412	409	538	535	537	5803	5580	5828
**Acetone**	406	409	405	531	530	530	5798	5582	5823
**EtOAc**	401	404	398	518	520	515	5633	5522	5708
**THF**	403	406	401	517	518	514	5472	5326	5482
**DCM**	418	421	415	523	524	521	4803	4669	4903
**CHCl_3_**	418	418	415	513	516	512	4430	4544	4565
**Toluene**	404	406	402	500	507	499	4752	4907	4836

λ_abs_: wavelength of maximum UV-Vis absorbance; λ_em_: wavelength of maximum emission intensity; : Stokes shift.

#### ii) Response to metal ions

The UV-Vis and fluorescence responses of conjugates **8**–**10** to a wide variety of metal ions (Ag^+^, Ba^2+^, Ca^2+^, Cd^2+^, Co^2+^, Cu^2+^, Fe^2+^, Fe^3+^, Hg^2+^, K^+^, Li^+^, Mg^2+^, Mn^2+^, Na^+^, Ni^2+^, Pb^2+^, Rb^+^ and Zn^2+^) were assessed in HEPES buffer (10 mM, pH 7.4 – see File S1). Of the metals tested, only Cu^2+^ triggered a significant response, quenching the fluorescence of all three conjugates (Figures S4–S6 in File S1). This response is consistent with previous observations that Cu^2+^ quenches the fluorescence of derivatives **1**, **3**, **6** and **7**, [20]–[22] and may be due to paramagnetic or heavy atom effects; [44]–[47] work is underway to determine the mechanism of Cu^2+^- mediated fluorescence quenching in these systems. However none of the cyclam-piperidinylnaphthalimide conjugates **8**–**10** show any meaningful response to either Zn^2+^ or Hg^2+^, in contrast to the previously-reported cyclam-naphthalimide conjugates **1**, **3** and **4** which exhibited fluorescence increases in the presence of Zn^2+^ and quenching in response to Hg^2+^ respectively. [20]–[22] Addition of Co^2+^, Fe^2+^ and Fe^3+^ each triggered a small to moderate reduction in the fluorescence of **10**, but had no effect on the fluorescence of **8** or **9**. Taken together, these fluorescence results imply that both the nature of the pendant fluorophore and the connectivity between the fluorophore and metal-cyclam complex play a role in the metal-ion responsiveness of these conjugates. The addition of these metal ions had no significant effect on the UV-Vis absorption spectra of all three conjugates (Figures S7–S9 in File S1).

**Figure 5:**
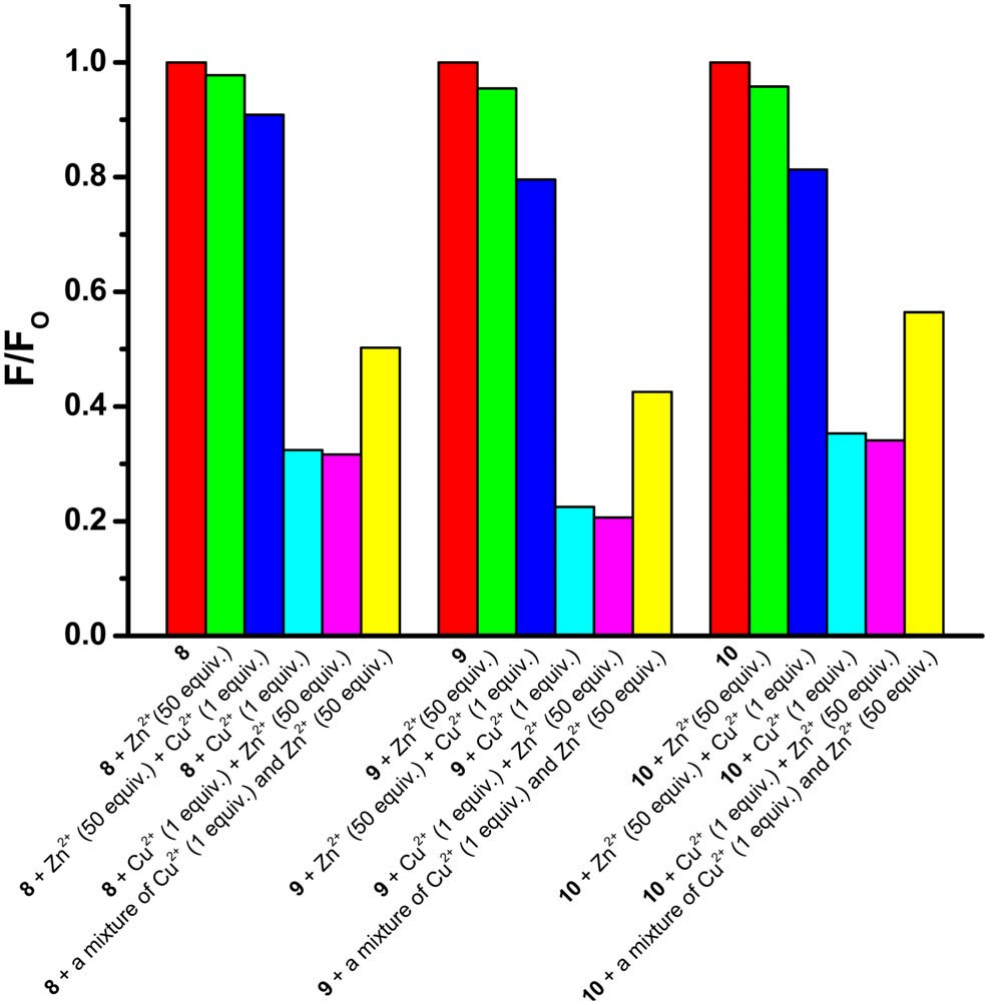
Competitive binding experiments. Experiments were carried out to investigate the effectiveness of Cu^2+^-induced (1 equiv.) quenching of the fluorescence of probes **8**–**10** (10 µM) in HEPES buffer (10 mM, pH 7.4) at 25°C in the presence of excess Zn^2+^ (50 equiv.).

To investigate the effectiveness of **8**–**10** as probes for Cu^2+^ in the presence of competing metal ions, competitive binding experiments were conducted using Zn^2+^. Thus a 10 µM solution of **8**–**10** in HEPES buffer (10 mM, pH 7.4) was combined with 50 equivalents of Zn^2+^, followed after approximately 3 minutes by 1 equivalent of Cu^2+^. In all cases, much weaker fluorescence quenching was observed than in the experiments in which the two metal ions were added in the reverse order, or a premixed Cu^2+^/Zn^2+^ (1∶50) solution was added (Figure 5). These results show the effectiveness of **8–10** as Cu^2+^- probes but indicate a limitation in the presence of high Zn^2+^ concentrations.

The fluorescence responses of probes **8**–**10** were evaluated over a wide pH range, both in the absence and presence of Cu^2+^ (Figures S10–S12 in File S1). These experiments indicate optimum responsiveness to Cu^2+^ at neutral pH. At low pH, the fluorescence responses of the free ligands **8**–**10** change little in the presence of Cu^2+^, presumably due to inhibition of metal coordination when the cyclam amine groups are protonated. The fluorescence of the free ligands **8**–**10** is diminished at high pH, as previously observed with probe **1**. [21] However, it is the absence of any protonation-induced fluorescence enhancement with probes **8**–**10** that is more significant. This indicates that the photoinduced electron transfer (PET) from the cyclam-triazole moiety to naphthalimide observed with probes **1**–**3** [22] does not occur with the piperidinylnaphthalimide fluorophore incumbent. This in turn means that the full fluorescence response of **8**–**10** is turned ‘on’ in the free ligands, eliminating the possibility of a fluorescence ‘turn-on’ pathway upon protonation or metal binding. The fact that PET is not favoured with probes **8**–**10** can be rationalized by considering the push-pull-character of the 4-aminonaphthalimide, where the electron acceptor is located at the amine and the electron donor at the imide. In the excited state, the resultant negative charge density on the imide inhibits acceptance of an additional electron *via* PET when the electron donor is connected at this position on the fluorophore [48].

#### iii) Time resolved photophysical properties and fluorescence quantum yields

Fluorescence quantum yields were acquired in three representative solvents (HEPES buffer, ethyl acetate and acetonitrile) to investigate the intrinsic photophysical properties in more detail (Table 2). In HEPES-buffer and acetonitrile, the quantum yields and the fluorescence decay times of the free ligands **8**–**10** are generally low, although ligand **9** gives a significantly longer decay time (<*τ*> = 4.86 ns) in buffer compared to ligands **8** and **10** (<*τ*> = 2.47 and 2.42 ns respectively). The quantum yields in ethyl acetate (0.25–0.50) are at least one order of magnitude higher than in acetonitrile (0.036–0.052) and about twice as high as in buffer (0.005–0.009). Clearly solvent has a strong influence on the photophysical properties of these probes. Strong solvent-dependence was also observed in the decay time profile of all three ligands. In ethyl acetate, ligands **8** and **10** decay with a single exponential profile, ligand **9** with a bis-exponential profile. In acetonitrile, decay times for all three ligands are fitted with two exponentials. In buffer, three exponentials give the best fit in all three cases. These multi-exponential fits indicate the presence of multiple excited species in these solvents. The additional components observed in aqueous buffer over the organic solvents can be rationalized by considering changes in ligand protonation, which give rise to new species that are absent in the aprotic organic solvents. Interestingly, the photophysical properties of **8**, **9** and **10** change relative to each other with changes in solvent: while similar values for quantum yields and decay times are observed for all three ligands in acetonitrile, only **8** and **10** afford similar data in ethyl acetate and buffer, and the values recorded for **9** are appreciably different. In ethyl acetate, longer decay times for **8** and **10** (5.73 and 6.36 ns respectively) and higher quantum yields (0.44 and 0.50) are found compared to ligand **9**, but in buffer ligand **9** gives higher values than ligands **8** and **10**. Notably, the averaged decay of **9** is particularly long (∼5 ns) in the aqueous solvent. The long decay times may render these new probes suitable for time-correlated assays, *e.g.* fluorescence lifetime imaging (FLIM) techniques in biological samples.

**Table 2:** Fluorescence quantum yields (*Φ*_F_) and decay times (*τ*) of 8–10 in HEPES buffer (10 mM, pH 7.4), ethyl acetate and acetonitrile.

Solvent	Compound	*Φ*_F_	*τ*/ns			<*τ*>/ns
**HEPES**	**8**	0.005	0.12 (28%)	2.32 (46%)	5.28 (26%)	2.47
	**9**	0.009	0.10 (9%)	4.03 (45%)	6.60 (46%)	4.86
	**10**	0.005	0.17 (26%)	1.83 (37%)	4.58 (37%)	2.42
**EtOAc**	**8**	0.44	5.73			5.73
	**9**	0.25	2.36 (29%)	5.13 (71%)		4.33
	**10**	0.50	6.36			6.36
**MeCN**	**8**	0.043	0.85 (94%)	8.38 (6%)		1.30
	**9**	0.036	0.80 (94%)	8.58 (6%)		1.27
	**10**	0.052	1.04 (95%)	7.18 (5%)		1.35

<*τ*> is the averaged time from the multi-exponential decay profiles.

### (c) Biological Evaluation

The cytotoxicity of cyclam-piperidinylnaphthalimide conjugates **8**–**10** and Boc-protected precursors **14**, **18**, and **21** was assayed against DLD-1 colon carcinoma cells and MDA-MB-231 breast carcinoma cells (Table 3). Cisplatin was used as a positive control in this cell viability study; its cytotoxicity against DLD-1 cells was found to be 11.2±0.3 µM, consistent with literature value of 11.8±1.2 µM. [49] Five of the six cyclam-piperidinylnaphthalimide compounds did not display any significant cytotoxicity against either cell line, with a safe dosage level of 20 µM. The single exception was conjugate **10**, which showed moderate activity against both carcinoma cell lines. In general, IC_50_ values for all cyclam-piperidinylnaphthalimide conjugates **8**–**10** were lower than those for the corresponding Boc-protected counterparts **14**, **18** and **21**.

**Table 3:** Cytotoxicity of 8–10, 14, 18 and 21 against DLD-1 colon carcinoma cells and MDA-MB-231 breast carcinoma cells after incubation for 72 h.

	IC_50_/µM	
Compound	DLD-1	MDA-MB-231
**cisplatin**	11.2±0.3	22.0±0.6
**8**	>100	54.1±0.8
**9**	>200	>100
**10**	53.8±1.3	37.4±0.6
**14**	>200	>200
**18**	>200	>100
**21**	>200	>200

IC_50_ values are expressed as mean ± standard error of mean of at least 3 independent experiments.

## Conclusions

We have reported the synthesis of three cyclam-piperidinylnaphthalimide conjugates **8**–**10** which respond to the presence of copper(II) with a significant decrease in fluorescence. Despite the different triazole connectivities and the variation of the pendant alkyl arm length, these probes exhibit remarkably similar photophysical properties. However, these photophysical properties are highly dependent on solvent, as seen in the UV-Vis and fluorescence spectra, quantum yields and decay times of all three ligands. The influence of the flexible ethyl linker is reflected in the long averaged fluorescence decay time of compound **9** in HEPES buffer, which is twice as long as those of ligands **8** and **10**. None of the probes display significant cytotoxicity to mammalian cells, supporting the potential suitability of this new probe class for sensing, labeling or imaging studies in biological systems.

## Experimental

### (a) General Materials

All reactions except azidation of **12** were carried out with continuous magnetic stirring in ordinary glassware; azidation of **12** was performed in a 15 mL Ace pressure tube, purchased from Sigma-Aldrich. Heating of reactions was conducted with a paraffin oil bath or a water bath. All reagents and solvents were purchased from Sigma-Aldrich, Alfa Acer, Merck, or Ajax Finechem. Reagents were used as received unless otherwise specified. Hexane and ethyl acetate were distilled before use. Dichloromethane and ethanol were distilled over calcium hydride and stored over activated 4 Å molecular sieves. Tetrahydrofuran was distilled over sodium wire/benzophenone. Methanol and acetonitrile were collected freshly from a PureSolv MD 7 solvent purification system having been passed through anhydrous alumina columns.

### (b) Instrumentation and Methods

^1^H and ^13^C NMR spectra were recorded at 300 K on a Bruker AVANCE 300 spectrometer (^1^H at 300.13 MHz and ^13^C at 75.47 MHz) or a Bruker DRX 400 spectrometer (^1^H at 400.13 MHz and ^13^C at 100.61 MHz). ^1^H and ^13^C NMR spectra are referenced to ^1^H signals of residual nondeuterated solvents (or tetramethylsilane) and ^13^C signals of the deuterated solvents respectively. ^1^H NMR signals are reported with chemical shift values *δ* (ppm), multiplicity (s = singlet, d = doublet, t = triplet, q = quartet, dd = doublet of doublets, m = multiplet and br = broad), relative integral, coupling constants *J* (Hz) and assignments. Infrared spectra were recorded on a Bruker Alpha FT-IR spectrometer. Low resolution and high resolution mass spectra were recorded on a Finnigan LCQ mass spectrometer and a Bruker 7T Fourier Transform Ion Cyclotron Resonance (FT-ICR) Mass Spectrometer respectively. Ionisation of all samples was carried out using either ESI or APCI. Melting points were determined on an OptiMelt 100 automated melting point apparatus and are uncorrected. Elemental analyses were carried out by the Campbell Microanalytical Laboratory (University of Otago, New Zealand) on a Carlo Erba EA 1108 Elemental Analyser. HEPES buffer was sterile filtered before use and the pH values were determined by a Mettler Toledo S20 SevenEasy™ pH meter or Minilab ISFET pH meter. Analytical TLC was performed on Merck silica gel 60 F_254_ pre-coated aluminum plates (0.2 mm) and visualized under UV light (254 nm), followed by staining with ninhydrin. Flash column chromatography was carried out using Merck silica gel 60 (0.040–0.063 mm). UV-Vis spectra were recorded on a Varian Cary 4000 or Varian Cary 1E UV-visible spectrophotometer. Fluorescence spectra were recorded on a Varian Cary Eclipse fluorescence spectrophotometer. Temperature control for both UV-visible spectrophotometer and fluorescence spectrophotometer was provided by a Varian Cary PCB water peltier system. In the time-resolved measurements, the frequency doubled output of Titan sapphire laser (Tsunami 3960; Spectra Physics) was used for excitation. The repetition rate of 80.2 MHz was reduced to 3.8 MHz with a Pulse Picker (Pulse Select; APE). The luminescence was detected in a right angle configuration to the incomming beam. For the detection, a multichannel plate (ELDI EM1-132/300; europhoton GmbH) coupled to a FL920 fluorescence lifetime spectrometer (Edinburgh Instruments) was used. The time-resolved emission was recorded in time-correlated single photon counting mode. The FAST software package (Edinburgh Instruments) was used to analyse the fluorescence decays. The fluorescence quantum yields were measured on a PL Quantum Yield Measurement System C9920-02 with an integrating sphere (Hamamatsu).

### (c) Synthesis

See the Supporting Information for synthetic experimental procedures of known compounds (Text S2 and Figures S13–S15 in File S1) and ^1^H and ^13^C NMR spectra of novel compounds (Figures S16–S33 in File S1).

#### General Synthetic Procedure A: The Copper(I)-Catalyzed Huisgen 1,3-Dipolar Cycloaddition of Azides and Alkynes

Alkyne (1.00 eq.) and azide (1.00 eq.) were dissolved in THF/H_2_O (7∶3, 50 mM in alkyne). A brown cloudy solution of CuSO_4_·5H_2_O (0.05 eq., 5 mol%) and sodium ascorbate (0.10 eq., 10 mol%) in H_2_O (25 mM in copper) was added. The reaction mixture was stirred at room temperature [23], [36], [37] or heated at 50°C under Ar for 12 h and quenched with saturated aqueous NH_4_Cl (100 L/mol copper). THF was evaporated under reduced pressure, and the remaining mixture was extracted with DCM (3×). The combined organic extracts were dried over Na_2_SO_4_ and concentrated under reduced pressure. The residue was purified by flash column chromatography (silica gel, EtOAc:petroleum benzine = 1∶1 ramping to EtOAc) to give the desired triazole.

#### General Synthetic Procedure B: TFA-Mediated Boc Removal [23], [36], [37] & Basification of Trifluoroacetates

Boc-protected amine (1.0 eq.) was dissolved in a mixture of TFA/DCM/H_2_O (90∶5∶5, 5 mM). The reaction mixture was stirred at room temperature for 6 h and concentrated under reduced pressure. The residue was dissolved in CH_3_OH (5 mL), and Ambersep 900 hydroxide form (pre-swelled with H_2_O for 30 min and CH_3_OH for 30 min) in CH_3_OH (10 mL) was added. The mixture was stirred at room temperature for 15 min and filtered, and the solid was washed with CH_3_OH (15 mL). The filtrate and washings were combined and concentrated under reduced pressure to give the desired *N*-functionalized cyclam.

#### 2-(4-Azidophenyl)-6-(piperidin-1-yl)-1*H*-benzo[*de*]isoquinoline-1,3(2*H*)-dione (13)

To a solution of **12** (218 mg, 0.501 mmol) and sodium azide (65.0 mg, 1.00 mmol) in THF/H_2_O (7 mL/3 mL) in an Ace pressure tube were added CuI (19 mg, 0.10 mmol), sodium ascorbate (10 mg, 0.050 mmol) and DMEDA (22 µL, 0.20 mmol). The reaction mixture was heated at reflux under Ar for 12 h and cooled to room temperature. After addition of H_2_O (7 mL), the reaction mixture was extracted with DCM (3×25 mL). The combined organic extracts were concentrated under reduced pressure, and the residue was purified by flash column chromatography (silica gel, DCM) to give **13** as a yellow solid (100 mg, 50%). ***R*** **_F_** (DCM) 0.66. **m.p.** 190–191°C. **IR** *ν*_max_/cm^−1^ 2936, 2853, 2794, 2112, 1705, 1657, 1589, 1506, 1452, 1375, 1287, 1234, 1195, 1143. **^1^H NMR** (300 MHz, CDCl_3_) *δ* 1.60–1.84 (m, 2H, CH_2_CH_2_C*H*_2_CH_2_CH_2_), 1.84–2.10 (m, 4H, CH_2_C*H*_2_CH_2_C*H*_2_CH_2_), 3.26 (t, 4H, *J* 5.1, CH_2_NCH_2_), 7.18 (d, 2H, *J* 8.4, Ph-H), 7.19 (d, 1H, *J* 7.5, naphthalene-H), 7.30 (d, 2H, *J* 8.7, Ph-H), 7.70 (t, 1H, *J* 7.8, naphthalene-H), 8.43 (d, 1H, *J* 8.4, naphthalene-H), 8.51 (d, 1H, *J* 8.1, naphthalene-H), 8.59 (d, 1H, *J* 7.5, naphthalene-H). **^13^C NMR** (75 MHz, CDCl_3_) *δ* 24.4, 26.3, 54.6, 114.9, 115.7, 120.0, 123.2, 125.5, 126.4, 130.3, 130.4, 131.2, 131.6, 132.5, 133.3, 140.2, 157.8, 164.3, 164.8 (four carbon signals overlapping or obscured). **HRMS** (ESI) 420.14297 ([M+Na]^+^); calcd. for C_23_H_19_N_5_NaO_2_ ([M+Na]^+^) 420.14310.

#### Tri-*tert*-butyl 11-((1-(4-(1,3-dioxo-6-(piperidin-1-yl)-1*H*-benzo[*de*]isoquinolin-2(3*H*)-yl)phenyl)-1*H*-1,2,3-triazol-4-yl)methyl)-1,4,8,11-tetraazacyclotetradecane-1,4,8-tricarboxylate (14)

Propargyl-tri-Boc cyclam [23], [36] (84 mg, 0.16 mmol) and azide **13** (62 mg, 0.16 mmol) were reacted at room temperature using general synthetic procedure A to give **14** as a yellow foam (140 mg, 96%). ***R*** **_F_** (EtOAc:hexane = 1∶1) 0.19. **IR** *ν*_max_/cm^−1^ 2973, 2934, 2857, 2816, 1670, 1584, 1517, 1460, 1413, 1365, 1322, 1298, 1234, 1160, 1075, 1039, 995, 915, 857, 832. **^1^H NMR** (300 MHz, CDCl_3_) *δ* 1.44 (s, 9H, C(CH_3_)_3_), 1.46 (s, 18H, 2×C(CH_3_)_3_), 1.70–1.85 (m, 4H, CH_2_CH_2_C*H*_2_CH_2_CH_2_ & NCH_2_C*H*_2_CH_2_N), 1.85–2.02 (m, 6H, CH_2_C*H*_2_CH_2_C*H*_2_CH_2_ & NCH_2_C*H*_2_CH_2_N), 2.45–2.60 (m, 2H, C*H*_2_N(CH_2_-triazole)CH_2_), 2.62–2.78 (m, 2H, CH_2_N(CH_2_-triazole)C*H*_2_), 3.28 (t, 4H, *J* 4.8, C*H*_2_N(naphthalene)C*H*_2_), 3.20–3.54 (m, 12H, 3×C*H*_2_N(Boc)C*H*_2_), 3.91 (s, 2H, NC*H*_2_-triazole), 7.22 (d, 1H, *J* 8.1, naphthalene-H), 7.49 (d, 2H, *J* 8.7, Ph-H), 7.72 (t, 1H, *J* 7.8, naphthalene-H), 7.96 (d, 2H, *J* 8.4, Ph-H), 8.08 (br s, 1H, triazole-H), 8.46 (d, 1H, *J* 8.4, naphthalene-H), 8.54 (d, 1H, *J* 8.1, naphthalene-H), 8.62 (d, 1H, *J* 7.2, naphthalene-H). **^13^C NMR** (75 MHz, CDCl_3_) *δ* 24.4, 26.3, 28.6, 45.7, 47.4, 48.9, 51.4, 54.6, 79.7, 114.9, 115.6, 121.2, 123.1, 125.5, 126.4, 130.5, 131.4, 131.6, 133.3, 136.0, 136.9, 144.4, 155.6, 155.9, 157.9, 164.1, 164.7 (twenty four carbon signals overlapping or obscured). **MS** (ESI) *m/z* 936.0 ([M+H]^+^, 48%), 958.1 ([M+Na]^+^, 100%). **HRMS** (ESI) 936.53398 ([M+H]^+^); calcd. for C_51_H_70_N_9_O_8_ ([M+H]^+^) 936.53419.

#### 2-(4-(4-((1,4,8,11-Tetraazacyclotetradecan-1-yl)methyl)-1*H*-1,2,3-triazol-1-yl)phenyl)-6-(piperidin-1-yl)-1*H*-benzo[*de*]isoquinoline-1,3(2*H*)-dione (8)

Compound **14** (112 mg, 0.120 mmol) was deprotected using general synthetic procedure B to give **8** as a yellow glue (73 mg, 96%). **IR** *ν*_max_/cm^−1^ 3384, 3287, 3123, 3058, 2926, 2850, 2820, 1701, 1660, 1584, 1517, 1457, 1367, 1233, 1189, 1135, 1114, 1078, 1044, 998, 833. **^1^H NMR** (400 MHz, CDCl_3_) *δ* 1.56–1.81 (m, 4H, CH_2_CH_2_C*H*_2_CH_2_CH_2_ & NCH_2_C*H*_2_CH_2_N), 1.81–2.10 (m, 6H, CH_2_C*H*_2_CH_2_C*H*_2_CH_2_ & NCH_2_C*H*_2_CH_2_N), 2.40–3.10 (m, 19H, 3×CH_2_NHCH_2_ & C*H*_2_N(CH_2_-triazole)C*H*_2_), 3.15–3.40 (m, 4H, C*H*_2_N(naphthalene)C*H*_2_), 3.93 (s, 2H, NC*H*_2_-triazole), 7.20 (d, 1H, *J* 8.0, naphthalene-H), 7.47 (d, 2H, *J* 8.4, Ph-H), 7.70 (t, 1H, *J* 8.0, naphthalene-H), 7.92 (d, 2H, *J* 8.4, Ph-H), 8.11 (s, 1H, triazole-H), 8.44 (d, 1H, *J* 8.4, naphthalene-H), 8.51 (d, 1H, *J* 8.0, naphthalene-H), 8.58 (d, 1H, *J* 7.2, naphthalene-H). **^13^C NMR** (100 MHz, CDCl_3_) *δ* 24.3, 26.1, 28.8, 46.9, 47.1, 48.0, 48.8, 49.3, 49.5, 50.7, 52.9, 54.5, 54.7, 114.7, 115.3, 120.8, 121.0, 122.9, 125.4, 126.3, 130.3, 130.4, 131.2, 131.5, 133.2, 135.8, 136.9, 145.0, 157.8, 163.9, 164.5 (five carbon signals overlapping or obscured). **MS** (ESI) *m/z* 636.3 ([M+H]^+^, 100%). **HRMS** (ESI) 636.37673 ([M+H]^+^); calcd. for C_36_H_46_N_9_O_2_ ([M+H]^+^) 636.37690.

#### Tri-*tert*-butyl 11-((1-(2-(1,3-dioxo-6-(piperidin-1-yl)-1*H*-benzo[*de*]isoquinolin-2(3*H*)-yl)ethyl)-1*H*-1,2,3-triazol-4-yl)methyl)-1,4,8,11-tetraazacyclotetradecane-1,4,8-tricarboxylate (18)

Propargyl-tri-Boc cyclam [23], [36] (298 mg, 0.553 mmol) and azide **17** (193 mg, 0.552 mmol) were reacted at room temperature using general synthetic procedure A to give **18** as a yellow foam (453 mg, 92%). ***R*** **_F_** (EtOAc:hexane = 1∶1) 0.19. **IR** *ν*_max_/cm^−1^ 2973, 2934, 2859, 2815, 1689, 1659, 1585, 1459, 1363, 1240, 1162, 1035, 864. **^1^H NMR** (400 MHz, CDCl_3_) *δ* 1.44 (s, 9H, C(CH_3_)_3_), 1.47 (s, 18H, 2×C(CH_3_)_3_), 1.63–1.78 (m, 4H, CH_2_CH_2_C*H*_2_CH_2_CH_2_ & NCH_2_C*H*_2_CH_2_N), 1.83–1.99 (m, 6H, CH_2_C*H*_2_CH_2_C*H*_2_CH_2_ & NCH_2_C*H*_2_CH_2_N), 2.28–2.42 (m, 2H, C*H*_2_N(CH_2_-triazole)CH_2_), 2.49–2.61 (m, 2H, CH_2_N(CH_2_-triazole)C*H*_2_), 3.23 (t, 4H, *J* 5.2, C*H*_2_N(naphthalene)C*H*_2_), 3.18–3.52 (m, 12H, 3×C*H*_2_N(Boc)C*H*_2_), 3.78 (s, 2H, NC*H*_2_-triazole), 4.64 (t, 2H, *J* 6.0), 4.75 (t, 2H, *J* 6.0) (total 4H, triazole-C*H*_2_C*H*_2_N), 7.15 (d, 1H, *J* 8.4, naphthalene-H), 7.55 (br s, 1H, triazole-H), 7.64 (dd, 1H, *J* 8.4 & 7.2, naphthalene-H), 8.37 (dd, 1H, *J* 8.4 & 1.2, naphthalene-H), 8.40 (d, 1H, *J* 8.0, naphthalene-H), 8.47 (dd, 1H, *J* 7.6 & 1.2, naphthalene-H). **^13^C NMR** (100 MHz, CDCl_3_) *δ* 24.3, 26.2, 26.6, 28.5, 28.6, 39.5, 45.2, 47.0, 47.9, 50.7, 51.7, 53.0, 54.5, 79.5, 114.7, 115.1, 122.5, 123.1, 125.3, 126.3, 130.1, 131.1, 131.3, 133.0, 143.0, 155.5, 155.8, 157.7, 163.7, 164.3 (seventeen carbon signals overlapping or obscured). **MS** (ESI) *m/z* 588.2 ([M-3Boc+H]^+^, 22%), 688.0 ([M-2Boc+H]^+^, 16%), 788.2 ([M-Boc+H]^+^, 31%), 888.2 ([M+H]^+^, 100%), 910.3 ([M+Na]^+^, 46%). **HRMS** (ESI) 910.51580 ([M+Na]^+^); calcd. for C_47_H_69_N_9_NaO_8_ ([M+Na]^+^) 910.51613.

#### 2-(2-(4-((1,4,8,11-Tetraazacyclotetradecan-1-yl)methyl)-1*H*-1,2,3-triazol-1-yl)ethyl)-6-(piperidin-1-yl)-1*H*-benzo[*de*]isoquinoline-1,3(2*H*)-dione (9)

Compound **18** (133 mg, 0.150 mmol) was deprotected using general synthetic procedure B to give **9** as a yellow glue (87 mg, 99%). **IR** *ν*_max_/cm^−1^ 3269, 2934, 2846, 2814, 1692, 1653, 1584, 1516, 1458, 1384, 1356, 1239, 1118, 1079, 1028. **^1^H NMR** (400 MHz, CDCl_3_) *δ* 1.50–2.00 (m, 10H, CH_2_C*H*_2_C*H*_2_C*H*_2_CH_2_ & 2×NCH_2_C*H*_2_CH_2_N), 2.20–2.84 (m, 16H, 3×C*H*_2_NHC*H*_2_ & C*H*_2_N(CH_2_-triazole)C*H*_2_), 3.95–3.50 (m, 7H, 3×CH_2_N*H*CH_2_ & C*H*_2_N(naphthalene)C*H*_2_), 3.69 (s, 2H, NC*H*_2_-triazole), 4.54 (t, 2H, *J* 5.2), 4.65 (t, 2H, *J* 5.2) (total 4H, triazole-C*H*_2_C*H*_2_N), 7.04 (d, 1H, *J* 8.0, naphthalene-H), 7.54 (t, 1H, *J* 7.6, naphthalene-H), 7.60 (s, 1H, triazole-H), 8.26 (d, 1H, *J* 6.8, naphthalene-H), 8.28 (d, 1H, *J* 7.6, naphthalene-H), 8.36 (d, 1H, *J* 6.8, naphthalene-H). **^13^C NMR** (100 MHz, CDCl_3_) *δ* 24.2, 25.7, 26.1, 28.4, 39.5, 46.7, 47.0, 47.6, 47.8, 48.6, 49.2, 50.7, 52.4, 54.0, 54.4, 114.6, 114.9, 122.3, 123.0, 125.2, 126.1, 129.9, 131.0, 131.1, 132.8, 143.9, 157.6, 163.5, 164.1 (three carbon signals overlapping or obscured). **MS** (ESI) *m/z* 588.2 ([M+H]^+^, 100%). **HRMS** (ESI) 588.37694 ([M+H]^+^); calcd. for C_32_H_46_N_9_O_2_ ([M+H]^+^) 588.37690.

#### 6-(Piperidin-1-yl)-2-(4-((trimethylsilyl)ethynyl)phenyl)-1*H*-benzo[*de*]isoquinoline-1,3(2*H*)-dione (19) [32]

To a solution of **12** (1.31 g, 3.01 mmol), CuI (269 mg, 1.41 mmol), triphenylphosphine (1.06 g, 4.04 mmol) and Pd(PPh_3_)_4_ (263 mg, 0.228 mmol) in Et_3_N (18 mL) were added pyridine (9 mL) and trimethylsilylacetylene (4.25 mL, 30.1 mmol). The reaction mixture was heated at 85°C under Ar overnight and cooled to room temperature before addition of DCM (40 mL). The organic phase was washed with H_2_O (4×40 mL), dried over MgSO_4_ and concentrated under reduced pressure. The residue was purified by flash column chromatography (silica gel, DCM:hexane = 5∶1) to give **19** as a yellow solid (1.28 g, 94%). ***R*** **_F_** (DCM) 0.67. **m.p.** 258–259°C. **IR** *ν*_max_/cm^−1^ 2959, 2926, 2854, 2794, 2364, 2332, 2156, 1707, 1658, 1590, 1508, 1453, 1401, 1376, 1237, 1196, 1148, 869, 838. **^1^H NMR** (300 MHz, CDCl_3_) *δ* 0.27 (s, 9H, Si(CH_3_)_3_), 1.65–1.80 (m, 2H, CH_2_CH_2_C*H*_2_CH_2_CH_2_), 1.84–1.98 (m, 4H, CH_2_C*H*_2_CH_2_C*H*_2_CH_2_), 3.25 (t, 4H, *J* 5.1, CH_2_NCH_2_), 7.18 (d, 1H, *J* 8.1, naphthalene-H), 7.24 (d, 2H, *J* 8.1, Ph-H), 7.61 (d, 2H, *J* 8.4, Ph-H), 7.69 (t, 1H, *J* 7.8, naphthalene-H), 8.43 (d, 1H, *J* 8.4, naphthalene-H), 8.50 (d, 1H, *J* 8.1, naphthalene-H), 8.58 (d, 1H, *J* 7.5, naphthalene-H). **^13^C NMR** (75 MHz, CDCl_3_) *δ* 0.1, 24.4, 26.3, 54.6, 95.1, 104.7, 114.9, 115.8, 123.2, 123.6, 125.5, 126.4, 128.9, 130.4, 131.2, 131.5, 132.9, 133.2, 135.9, 157.8, 164.1, 164.6 (six carbon signals overlapping or obscured). **HRMS** (ESI) 453.19960 ([M+H]^+^); calcd. for C_28_H_29_N_2_O_2_Si ([M+H]^+^) 453.19928.

#### 2-(4-Ethynylphenyl)-6-(piperidin-1-yl)-1*H*-benzo[*de*]isoquinoline-1,3(2*H*)-dione (20) [32]

To a solution of **19** (78 mg, 0.17 mmol) in CH_3_OH (5 mL) was added K_2_CO_3_ (95 mg, 0.69 mmol). The reaction mixture was stirred at room temperature overnight and filtered. The solids were washed with H_2_O (3×5 mL), dried and purified by flash column chromatography (silica gel, DCM:hexane = 1∶1 ramping to DCM) to give **20** as a yellow solid (63 mg, 97%). ***R*** **_F_** (DCM:hexane = 5∶1) 0.50. **m.p.** 263–264°C. **IR** *ν*_max_/cm^−1^ 3254, 2943, 2917, 2850, 2807, 2361, 2331, 1696, 1647, 1585, 1508, 1449, 1372, 1233, 1187, 1138, 1077, 1034, 1000, 914, 832. **^1^H NMR** (400 MHz, CDCl_3_) *δ* 1.70–1.77 (m, 2H, CH_2_CH_2_C*H*_2_CH_2_CH_2_), 1.87–1.94 (m, 4H, CH_2_C*H*_2_CH_2_C*H*_2_CH_2_), 3.12 (s, 1H, C≡CH), 3.26 (t, 4H, *J* 5.2, CH_2_NCH_2_), 7.20 (d, 1H, *J* 8.0, naphthalene-H), 7.28 (d, 2H, *J* 8.4, Ph-H), 7.65 (d, 2H, *J* 8.4, Ph-H), 7.70 (t, 1H, *J* 8.0, naphthalene-H), 8.44 (d, 1H, *J* 8.4, naphthalene-H), 8.52 (d, 1H, *J* 8.0, naphthalene-H), 8.60 (d, 1H, *J* 7.2, naphthalene-H). **^13^C NMR** (75 MHz, CDCl_3_) *δ* 24.5, 26.4, 54.7, 83.3, 114.9, 115.8, 122.6, 123.2, 125.6, 126.5, 129.1, 130.5, 131.3, 131.6, 133.2, 133.3, 136.3, 157.9, 164.2, 164.7 (five carbon signals overlapping or obscured). **HRMS** (ESI) 403.14150 ([M+Na]^+^); calcd. for C_25_H_20_N_2_NaO_2_ ([M+Na]^+^) 403.14170.

#### Tri-*tert*-butyl 11-(2-(4-(4-(1,3-dioxo-6-(piperidin-1-yl)-1*H*-benzo[*de*]isoquinolin-2(3*H*)-yl)phenyl)-1*H*-1,2,3-triazol-1-yl)ethyl)-1,4,8,11-tetraazacyclotetradecane-1,4,8-tricarboxylate (21)

Azide **S3** (382 mg, 0.670 mmol) and alkyne **20** (255 mg, 0.670 mmol) were reacted at 50°C using general synthetic procedure A to give **21** as a yellow foam (423 mg, 66%). ***R*** **_F_** (EtOAc:hexane = 1∶1) 0.17. **IR** *ν*_max_/cm^−1^ 2973, 2937, 2861, 2817, 1689, 1584, 1462, 1413, 1366, 1235, 1161, 1074. **^1^H NMR** (300 MHz, CDCl_3_) *δ* 1.46 (s, 9H, C(CH_3_)_3_), 1.47 (s, 18H, 2×C(CH_3_)_3_), 1.62–1.85 (m, 6H, CH_2_CH_2_C*H*_2_CH_2_CH_2_ & 2×NCH_2_C*H*_2_CH_2_N), 1.85–2.00 (m, 4H, CH_2_C*H*_2_CH_2_C*H*_2_CH_2_), 2.46–2.62 (m, 2H, C*H*_2_N(CH_2_CH_2_-triazole)CH_2_), 2.62–2.79 (m, 2H, CH_2_N(CH_2_CH_2_-triazole)C*H*_2_), 2.92–3.10 (m, 2H, NC*H*_2_CH_2_-triazole), 3.10–3.50 (m, 16H, 3×C*H*_2_N(Boc)C*H*_2_ & C*H*_2_N(naphthalene)C*H*_2_), 4.36–4.53 (m, 2H, NCH_2_C*H*_2_-triazole), 7.21 (d, 1H, *J* 8.1, naphthalene-H), 7.38 (d, 2H, *J* 8.1, Ph-H), 7.71 (t, 1H, *J* 7.8, naphthalene-H), 7.91 (br s, 1H, triazole-H), 8.01 (d, 2H, *J* 8.1, Ph-H), 8.45 (d, 1H, *J* 8.4, naphthalene-H), 8.53 (d, 1H, *J* 8.1, naphthalene-H), 8.61 (d, 1H, *J* 7.2, naphthalene-H). **^13^C NMR** (75 MHz, CDCl_3_) *δ* 24.4, 26.3, 28.6, 45.9, 47.0, 47.5, 47.9, 48.3, 52.4, 53.5, 54.6, 55.1, 79.8, 79.9, 114.8, 115.9, 120.7, 123.3, 125.5, 126.4, 126.5, 129.4, 130.4, 130.9, 131.1, 131.5, 133.2, 135.6, 147.1, 155.6, 155.8, 157.7, 164.2, 164.8 (eighteen carbon signals overlapping or obscured). **MS** (ESI) *m/z* 972.2 ([M+Na]^+^, 100%). **HRMS** (ESI) 972.53168 ([M+Na]^+^); calcd. for C_52_H_71_N_9_NaO_8_ ([M+Na]^+^) 972.53178.

#### 2-(4-(1-(2-(1,4,8,11-Tetraazacyclotetradecan-1-yl)ethyl)-1*H*-1,2,3-triazol-4-yl)phenyl)-6-(piperidin-1-yl)-1*H*-benzo[*de*]isoquinoline-1,3(2*H*)-dione (10)

Compound **21** (143 mg, 0.150 mmol) was deprotected using general synthetic procedure B to give **10** as a yellow glue (97 mg, 99%). **IR** *ν*_max_/cm^−1^ 3282, 3056, 2933, 2814, 1700, 1659, 1582, 1509, 1457, 1363, 1230, 1188, 1134, 1076, 1045, 1000, 912, 830. **^1^H NMR** (400 MHz, CDCl_3_) *δ* 1.60–1.82 (m, 6H, CH_2_CH_2_C*H*_2_CH_2_CH_2_ & 2×NCH_2_C*H*_2_CH_2_N), 1.82–2.00 (m, 4H, CH_2_C*H*_2_CH_2_C*H*_2_CH_2_), 2.20–2.85 (m, 19H, 3×CH_2_NHCH_2_ & C*H*_2_N(CH_2_CH_2_-triazole)C*H*_2_), 2.94 (t, 2H, *J* 6.0, NC*H*_2_CH_2_-triazole), 3.12–3.42 (m, 4H, C*H*_2_N(naphthalene)C*H*_2_), 4.57 (t, 2H, *J* 6.0, NCH_2_C*H*_2_-triazole), 7.19 (d, 1H, *J* 8.0, naphthalene-H), 7.37 (d, 2H, *J* 8.4, Ph-H), 7.69 (t, 1H, *J* 8.0, naphthalene-H), 8.01 (d, 2H, *J* 8.0, Ph-H), 8.20 (s, 1H, triazole-H), 8.43 (d, 1H, *J* 8.4, naphthalene-H), 8.50 (d, 1H, *J* 8.0, naphthalene-H), 8.58 (d, 1H, *J* 6.8, naphthalene-H). **^13^C NMR** (100 MHz, CDCl_3_) *δ* 24.3, 26.1, 28.5, 46.7, 47.2, 47.6, 48.3, 48.6, 50.7, 51.2, 52.9, 54.4, 54.6, 114.7, 115.7, 121.7, 123.1, 125.3, 126.3, 129.3, 130.3, 130.9, 131.0, 131.3, 132.9, 135.5, 146.5, 157.5, 164.0, 164.5 (seven carbon signals overlapping or obscured). **MS** (ESI) *m/z* 650.2 ([M+H]^+^, 100%), 1299.1 ([2M+H]^+^, 30%). **HRMS** (ESI) 650.39253 ([M+H]^+^); calcd. for C_37_H_48_N_9_O_2_ ([M+H]^+^) 650.39255.

### (d) Photophysical Studies

All UV-Vis and fluorescence experiments were performed with a 1 cm fluorescence quartz cuvette. For metal ion binding studies, a small amount (2–10 µL) of a solution of metal perchlorate (2–10 mM in metal) in HEPES buffer (10 mM, pH 7.4) was added to a solution of **8**–**10** (10 µM, 2 mL) in HEPES buffer (10 mM, pH 7.4). For competitive binding studies, 50 equivalents of Zn^2+^ or Cu^2+^ were added to a 10 µM solution of **8**–**10** in HEPES buffer (10 mM, pH 7.4), followed after approximately 3 min by addition of 1 equivalent of Cu^2+^ or Zn^2+^ respectively; a premixed Cu^2+^/Zn^2+^ (1 equivalent/50 equivalents) solution was added to a 10 µM solution of **8**–**10** in HEPES buffer (10 mM, pH 7.4). For pH studies, the pH value of a solution of **8**–**10** (10 µM, 2 mL) in HEPES buffer (10 mM, pH 7.4) was adjusted with either 1 M HClO_4_ or 1 M NaOH prior to addition of Cu^2+^. For the solvent studies, solutions of **8**–**10** (1–2 µM, 2 mL) were prepared in different solvents. For the quantum yield and time resolved measurements in HEPES-buffer, ethyl acetate and MeCN, solutions were prepared freshly and the absorbance was adjusted to 0.1.

### (e) Cell Viability Assay

The cytotoxicity of compounds **8**–**10**, **14**, **18** and **21** was evaluated using cell viability assay as described previously. [50] Compounds **8**–**10**, **14**, **18** and **21** were prepared as a 10 mM stock solution in DMSO and diluted with growth medium (2% FCS and 1% glutamine) to give rise to a range of concentrations (0–200 µM). DLD-1 colon carcinoma cells and MDA-MB-231 breast carcinoma cells were cultured as monolayers in Advanced DMEM, supplemented with 2% FCS, 1% glutamine and 1% antibiotic/antimycotic (A/A). Cells were incubated at 37°C with 5% CO_2_ in a humidified incubator, seeded at 1×10^4^ cells per well of a 96-well plate in 100 µL of growth medium, and allowed to adhere for 15 h. Growth medium was removed, and 100 µL of compounds at different concentrations were added in triplicate. The plates were incubated for 72 h. 3-(4,5-Dimethylthiazol-2-yl)-2,5-diphenyltetrazolium bromide (MTT, 20 µL), a water-aqueous soluble yellow tetrazole compound, was added to a final concentration of 1 mM per well and the plates were incubated for 4 h. Growth medium was removed, and DMSO (150 µL) was added to dissolve the water-insoluble purple formazan crystals. The plates were shaken until all the crystals were dissolved. The absorbances at 600 nm were read by a microplate reader (Victor, PerkinElmer) and averaged for each concentration. IC_50_ value was determined by the concentration of the compound at which the absorbance was half of that of the cells grown in only growth medium. The average of three independent IC_50_ values for each concentration was used to calculate the standard error of the mean.

## Supporting Information

File S1 **File S1 Contains the files: Text S1. Lippert-Mataga Equation. Text S2. Synthesis of Known Compounds. Figure S1. Stokes shift () of 8 versus orientation polarizability (Δ** ***f*** **).** The red, straight line represents the best linear fit to the 13 data points [coefficient of determination *R* ^2^ = 0.560, slope = (4.32±1.07)×10^3^ cm^−1^, intercept = (4.41±0.26)×10^3^ cm^−1^]. **Figure S2. Stokes shift () of 9 versus orientation polarizability (Δ** ***f*** **).** The red, straight line represents the best linear fit to the 13 data points [coefficient of determination *R* ^2^ = 0.392, slope = (3.00±1.02)×10^3^ cm^−1^, intercept = (4.61±0.25)×10^3^ cm^−1^]. **Figure S3. Stokes shift () of 10 versus orientation polarizability (Δ** ***f*** **).** The red, straight line represents the best linear fit to the 13 data points [coefficient of determination *R* ^2^ = 0.562, slope = (4.07±1.00)×10^3^ cm^−1^, intercept = (4.53±0.25)×10^3^ cm^−1^]. **Figure S4. Fluorescence spectra of 8 (10 µM) in the presence of various metal ions.** Experiments were carried out in HEPES buffer (10 mM, pH 7.4) at 25°C and the fluorescence emission spectra were recorded about 5 min after addition of various metal ions (1 equiv.). **Figure S5. Fluorescence spectra of 9 (10 µM) in the presence of various metal ions.** Experiments were carried out in HEPES buffer (10 mM, pH 7.4) at 25°C and the fluorescence emission spectra were recorded about 5 min after addition of various metal ions (1 equiv.). **Figure S6. Fluorescence spectra of 10 (10 µM) in the presence of various metal ions.** Experiments were carried out in HEPES buffer (10 mM, pH 7.4) at 25°C and the fluorescence emission spectra were recorded about 5 min after addition of various metal ions (1 equiv.). **Figure S7. UV-Vis spectra of 8 (10 µM) in the presence of various metal ions.** Experiments were carried out in HEPES buffer (10 mM, pH 7.4) at 25°C and the UV-Vis spectra were recorded about 5 min after addition of various metal ions (1 equiv.). **Figure S8. UV-Vis spectra of 9 (10 µM) in the presence of various metal ions.** Experiments were carried out in HEPES buffer (10 mM, pH 7.4) at 25°C and the UV-Vis spectra were recorded about 5 min after addition of various metal ions (1 equiv.). **Figure S9. UV-Vis spectra of 10 (10 µM) in the presence of various metal ions.** Experiments were carried out in HEPES buffer (10 mM, pH 7.4) at 25°C and the UV-Vis spectra were recorded about 5 min after addition of various metal ions (1 equiv.). **Figure S10. Fluorescence emission of 8 (10 µM) in the absence and presence of Cu^2+^ (1 equiv.) over a range of pH values.** Experiments were carried out in HEPES buffer (10 mM, pH 7.4) at 25°C. **Figure S11. Fluorescence emission of 9 (10 µM) in the absence and presence of Cu^2+^ (1 equiv.) over a range of pH values.** Experiments were carried out in HEPES buffer (10 mM, pH 7.4) at 25°C. **Figure S12. Fluorescence emission of 10 (10 µM) in the absence and presence of Cu^2+^ (1 equiv.) over a range of pH values.** Experiments were carried out in HEPES buffer (10 mM, pH 7.4) at 25°C. **Figure S13. Synthesis of 2-azidoethyl-tri-Boc cyclam S3.** *Reagents and conditions*: (a) (i) NaN_3_, H_2_O, reflux, o/n; (ii) *p*-toluenesulfonyl chloride, Et_3_N, rt, 6 h; (iii) glycine, rt, 2 h, 65%; (b) tri-Boc cyclam, Na_2_CO_3_, CH_3_CN, reflux, 96 h, 66%. **Figure S14. Synthesis of compound 12.** *Reagents and conditions*: (a) piperidine, 2-methoxyethanol, reflux, 36 h, 86%; (b) 4-bromoaniline, piperidine, 2-methoxyethanol, reflux, 72 h, 90%. **Figure S15. Synthesis of compound 17.** *Reagents and conditions*: (a) 2-aminoethanol, EtOH, reflux, 22 h, 92%; (b) PBr_3_, pyridine, THF, 50°C, 16 h, 60%; (c) NaN_3_, EtOH, reflux, 6 h, 80%. **Figure S16. ^1^H NMR spectrum (300 MHz) of 13 in CDCl_3_. Figure S17. ^13^C NMR spectrum (75 MHz) of 13 in CDCl_3_. Figure S18. ^1^H NMR spectrum (300 MHz) of 14 in CDCl_3_. Figure S19. ^13^C NMR spectrum (75 MHz) of 14 in CDCl_3_. Figure S20. ^1^H NMR spectrum (400 MHz) of 8 in CDCl_3_. Figure S21. ^13^C NMR spectrum (100 MHz) of 8 in CDCl_3_. Figure S22. ^1^H NMR spectrum (400 MHz) of 18 in CDCl_3_. Figure S23. ^13^C NMR spectrum (100 MHz) of 18 in CDCl_3_. Figure S24. ^1^H NMR spectrum (400 MHz) of 9 in CDCl_3_. Figure S25. ^13^C NMR spectrum (100 MHz) of 9 in CDCl_3_. Figure S26. ^1^H NMR spectrum (300 MHz) of 19 in CDCl_3_. Figure S27. ^13^C NMR spectrum (75 MHz) of 19 in CDCl_3_. Figure S28. ^1^H NMR spectrum (400 MHz) of 20 in CDCl_3_. Figure S29. ^13^C NMR spectrum (75 MHz) of 20 in CDCl_3_. Figure S30. ^1^H NMR spectrum (300 MHz) of 21 in CDCl_3_. Figure S31. ^13^C NMR spectrum (75 MHz) of 21 in CDCl_3_. Figure S32. ^1^H NMR spectrum (400 MHz) of 10 in CDCl_3_. Figure S33. ^13^C NMR spectrum (100 MHz) of 10 in CDCl_3_.**
(ZIP)
