# Supplementary material for: Incorporating a Piperidinyl Group in the Fluorophore Extends the Fluorescence Lifetime of Click-Derived Cyclam-Naphthalimide Conjugates
Source: PLoS One. 2014 Jul 1;9(7):e100761. doi: 10.1371/journal.pone.0100761 (PMC4077572; doi:10.1371/journal.pone.0100761)
Supplement: File S1 — Contains the files: Text S1. Lippert-Mataga Equation. Text S2. Synthesis of Known Compounds. Figure S1. Stokes shift () of 8 versus orientation polarizability (Δ f ). The red, straight line represents the best linear fit to the 13 data points [coefficient of determination R 2 = 0.560, slope = (4.32±1.07)×103 cm−1, intercept = (4.41±0.26)×103 cm−1]. Figure S2. Stokes shift () of 9 versus orientation polarizability (Δ f ). The red, straight line represents the best linear fit to the 13 data points [coefficient of determination R 2 = 0.392, slope = (3.00±1.02)×103 cm−1, intercept = (4.61±0.25)×103 cm−1]. Figure S3. Stokes shift () of 10 versus orientation polarizability (Δ f ). The red, straight line represents the best linear fit to the 13 data points [coefficient of determination R 2 = 0.562, slope = (4.07±1.00)×103 cm−1, intercept = (4.53±0.25)×103 cm−1]. Figure S4. Fluorescence spectra of 8 (10 µM) in the presence of various metal ions. Experiments were carried out in HEPES buffer (10 mM, pH 7.4) at 25°C and the fluorescence emission spectra were recorded about 5 min after addition of various metal ions (1 equiv.). Figure S5. Fluorescence spectra of 9 (10 µM) in the presence of various metal ions. Experiments were carried out in HEPES buffer (10 mM, pH 7.4) at 25°C and the fluorescence emission spectra were recorded about 5 min after addition of various metal ions (1 equiv.). Figure S6. Fluorescence spectra of 10 (10 µM) in the presence of various metal ions. Experiments were carried out in HEPES buffer (10 mM, pH 7.4) at 25°C and the fluorescence emission spectra were recorded about 5 min after addition of various metal ions (1 equiv.). Figure S7. UV-Vis spectra of 8 (10 µM) in the presence of various metal ions. Experiments were carried out in HEPES buffer (10 mM, pH 7.4) at 25°C and the UV-Vis spectra were recorded about 5 min after addition of various metal ions (1 equiv.). Figure S8. UV-Vis spectra of 9 (10 µM) in the presence of various metal ions. Ex [file pone.0100761.s001.zip › SI/Text S1_Lippert-Mataga Equation_20140505.docx]

**Lippert-Mataga Equation[**[**1-5**](#_ENREF_1)**]**

*Lippert-Mataga plots of compounds* ***8****,* ***9****,* ***10*** *have been shown in Figures S13-S15.*

The interactions between the solvent and the fluorophore affect the energy difference between the ground and excited states, and are best interpreted by the Lippert-Mataga equation written below.

$$\Delta\bar{}=\frac{2\Delta f}{4\text{0}\text{h}ca\text{3}}(\mu\text{e}-\mu\text{g})\text{2}+constant$$

In this equation, $\Delta\bar{}$ is the solvatochromic Stokes shift (in cm^-1^) between the maxima of UV-Vis absorbance and fluorescence emission.

$\Delta\bar{}= \bar{}$_abs_$-\bar{}$_em_$=1/{}$_abs_$-$1⁄λ_em_

*f* is the polarizability of the solvent, which is a result of both mobility of electrons in the solvent and the dipole moment of the solvent molecules. Each of the two components has a different time dependence. Reorientation of electrons in the solvent is essentially instantaneous. The high frequency polarizability *f*(*n*) is a function of the refractive index (*n*).

$$f(n)=(n\text{2}-1)/(2n\text{2}+1)$$

The polarizability of the solvent is also contingent on the dielectric constant (ε), which includes the effect of molecular orientation of the solvent molecules. Molecular reorientation of the solvent molecules is typically slower. The low frequency polarizability *f*(ε) is a function of ε.

$$f(\varepsilon)=(\varepsilon-1)/(2\varepsilon+1)$$

The difference between these two terms is called the orientation polarizability (Δ*f*).

$$\Delta f=f(\varepsilon)-f(n)$$

ε_0_ is the permittivity of vacuum, *h* is Planck’s constant, *c* is the velocity of light, and *a* represents the radius of the cavity in which the solute resides. *μ*_g_ and *μ*_e_ denote the ground- and excited-state dipole moments of the fluorophore respectively.

**References**

1. Lippert E (1955) Dipolmoment und Elektronenstruktur von angeregten Molekülen. Z Naturforsch, A: Phys Sci 10: 541-545.

2. Mataga N, Kaifu Y and Koizumi M (1955) The Solvent Effect on Fluorescence Spectrum, Change of Solute-Solvent Interaction during the Lifetime of Excited Solute Molecule. Bull Chem Soc Jpn 28: 690-691.

3. Mataga N, Kaifu Y and Koizumi M (1956) Solvent Effects upon Fluorescence Spectra and the Dipolemoments of Excited Molecules. Bull Chem Soc Jpn 29: 465-470.

4. Lakowicz JR (2006) 6. Solvent and Environmental Effects. Principles of Fluorescence Spectroscopy. Singapore: Springer Science+Business Media. pp. 208-216.

5. Filarowski A, Kluba M, Cieslik-Boczula K, Koll A, Kochel A, et al. (2010) Generalized solvent scales as a tool for investigating solvent dependence of spectroscopic and kinetic parameters. Application to fluorescent BODIPY dyes. Photochem Photobiol Sci 9: 996-1008.
