# Supplementary figures and images for: Incorporating a Piperidinyl Group in the Fluorophore Extends the Fluorescence Lifetime of Click-Derived Cyclam-Naphthalimide Conjugates
Source: PLoS One. 2014 Jul 1;9(7):e100761. doi: 10.1371/journal.pone.0100761 (PMC4077572; doi:10.1371/journal.pone.0100761)

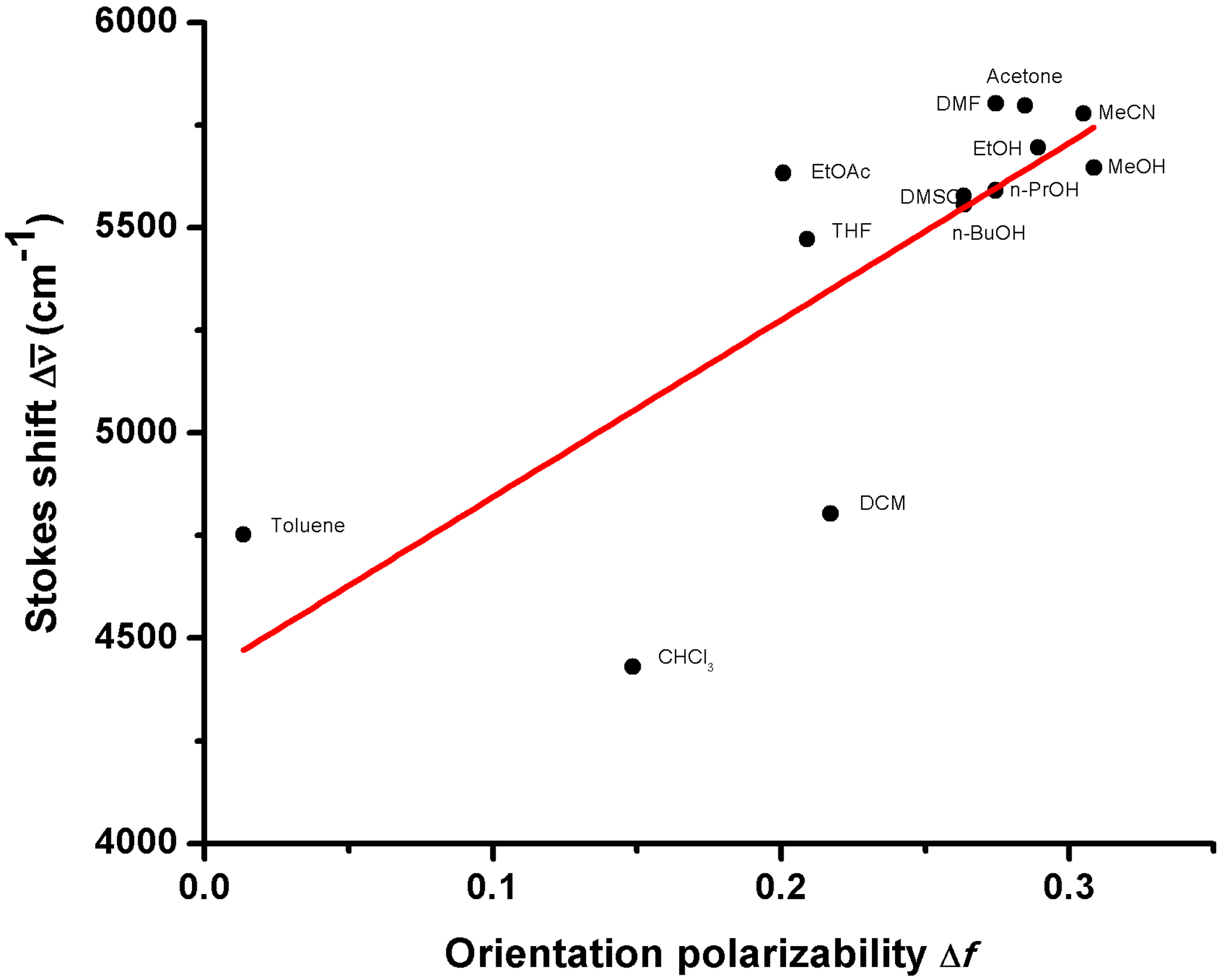

Supplement: File S1 — Contains the files: Text S1. Lippert-Mataga Equation. Text S2. Synthesis of Known Compounds. Figure S1. Stokes shift () of 8 versus orientation polarizability (Δ f ). The red, straight line represents the best linear fit to the 13 data points [coefficient of determination R 2 = 0.560, slope = (4.32±1.07)×103 cm−1, intercept = (4.41±0.26)×103 cm−1]. Figure S2. Stokes shift () of 9 versus orientation polarizability (Δ f ). The red, straight line represents the best linear fit to the 13 data points [coefficient of determination R 2 = 0.392, slope = (3.00±1.02)×103 cm−1, intercept = (4.61±0.25)×103 cm−1]. Figure S3. Stokes shift () of 10 versus orientation polarizability (Δ f ). The red, straight line represents the best linear fit to the 13 data points [coefficient of determination R 2 = 0.562, slope = (4.07±1.00)×103 cm−1, intercept = (4.53±0.25)×103 cm−1]. Figure S4. Fluorescence spectra of 8 (10 µM) in the presence of various metal ions. Experiments were carried out in HEPES buffer (10 mM, pH 7.4) at 25°C and the fluorescence emission spectra were recorded about 5 min after addition of various metal ions (1 equiv.). Figure S5. Fluorescence spectra of 9 (10 µM) in the presence of various metal ions. Experiments were carried out in HEPES buffer (10 mM, pH 7.4) at 25°C and the fluorescence emission spectra were recorded about 5 min after addition of various metal ions (1 equiv.). Figure S6. Fluorescence spectra of 10 (10 µM) in the presence of various metal ions. Experiments were carried out in HEPES buffer (10 mM, pH 7.4) at 25°C and the fluorescence emission spectra were recorded about 5 min after addition of various metal ions (1 equiv.). Figure S7. UV-Vis spectra of 8 (10 µM) in the presence of various metal ions. Experiments were carried out in HEPES buffer (10 mM, pH 7.4) at 25°C and the UV-Vis spectra were recorded about 5 min after addition of various metal ions (1 equiv.). Figure S8. UV-Vis spectra of 9 (10 µM) in the presence of various metal ions. Ex [file pone.0100761.s001.zip › SI/Figure S1.tif]

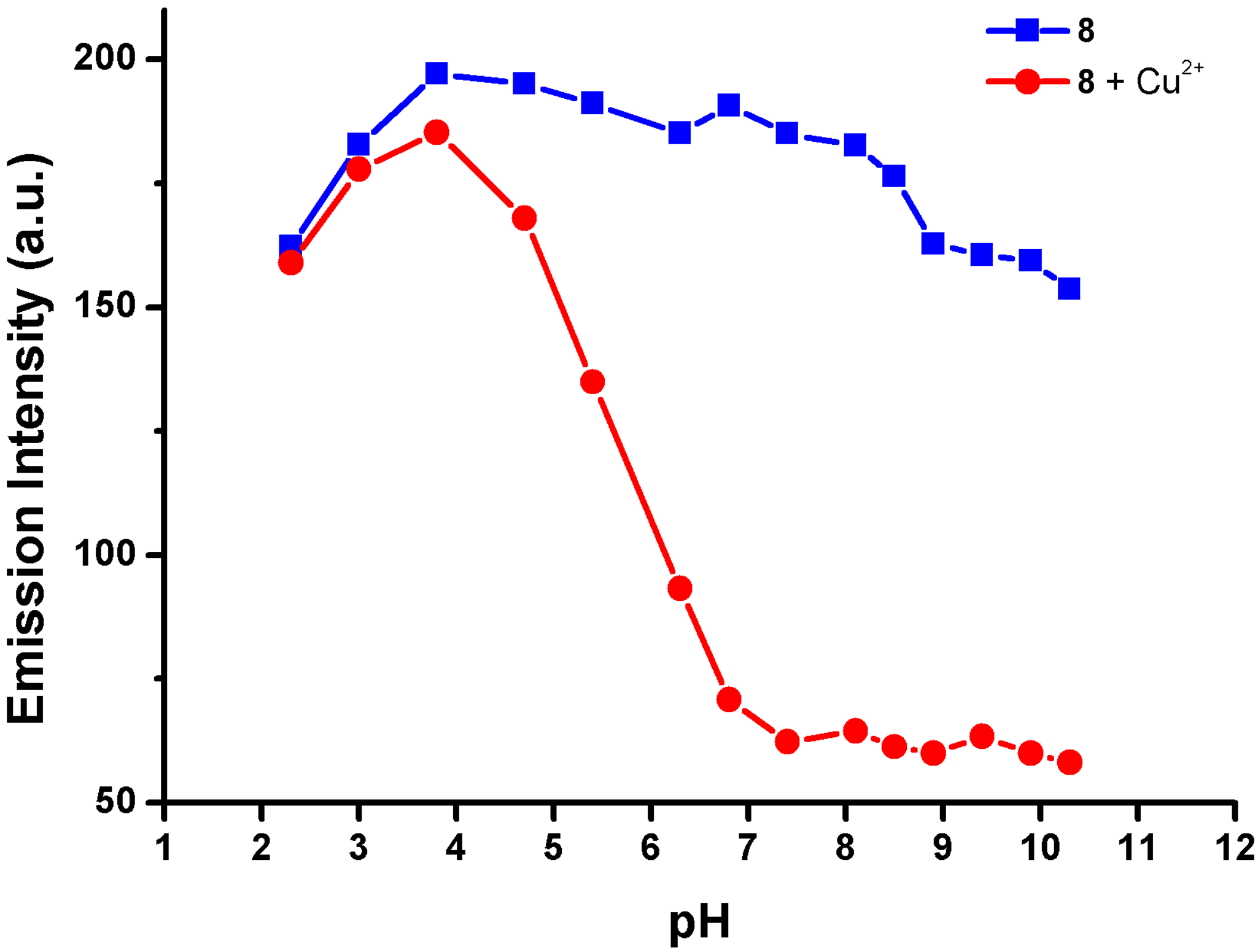

Supplement: File S1 — Contains the files: Text S1. Lippert-Mataga Equation. Text S2. Synthesis of Known Compounds. Figure S1. Stokes shift () of 8 versus orientation polarizability (Δ f ). The red, straight line represents the best linear fit to the 13 data points [coefficient of determination R 2 = 0.560, slope = (4.32±1.07)×103 cm−1, intercept = (4.41±0.26)×103 cm−1]. Figure S2. Stokes shift () of 9 versus orientation polarizability (Δ f ). The red, straight line represents the best linear fit to the 13 data points [coefficient of determination R 2 = 0.392, slope = (3.00±1.02)×103 cm−1, intercept = (4.61±0.25)×103 cm−1]. Figure S3. Stokes shift () of 10 versus orientation polarizability (Δ f ). The red, straight line represents the best linear fit to the 13 data points [coefficient of determination R 2 = 0.562, slope = (4.07±1.00)×103 cm−1, intercept = (4.53±0.25)×103 cm−1]. Figure S4. Fluorescence spectra of 8 (10 µM) in the presence of various metal ions. Experiments were carried out in HEPES buffer (10 mM, pH 7.4) at 25°C and the fluorescence emission spectra were recorded about 5 min after addition of various metal ions (1 equiv.). Figure S5. Fluorescence spectra of 9 (10 µM) in the presence of various metal ions. Experiments were carried out in HEPES buffer (10 mM, pH 7.4) at 25°C and the fluorescence emission spectra were recorded about 5 min after addition of various metal ions (1 equiv.). Figure S6. Fluorescence spectra of 10 (10 µM) in the presence of various metal ions. Experiments were carried out in HEPES buffer (10 mM, pH 7.4) at 25°C and the fluorescence emission spectra were recorded about 5 min after addition of various metal ions (1 equiv.). Figure S7. UV-Vis spectra of 8 (10 µM) in the presence of various metal ions. Experiments were carried out in HEPES buffer (10 mM, pH 7.4) at 25°C and the UV-Vis spectra were recorded about 5 min after addition of various metal ions (1 equiv.). Figure S8. UV-Vis spectra of 9 (10 µM) in the presence of various metal ions. Ex [file pone.0100761.s001.zip › SI/Figure S10.tif]

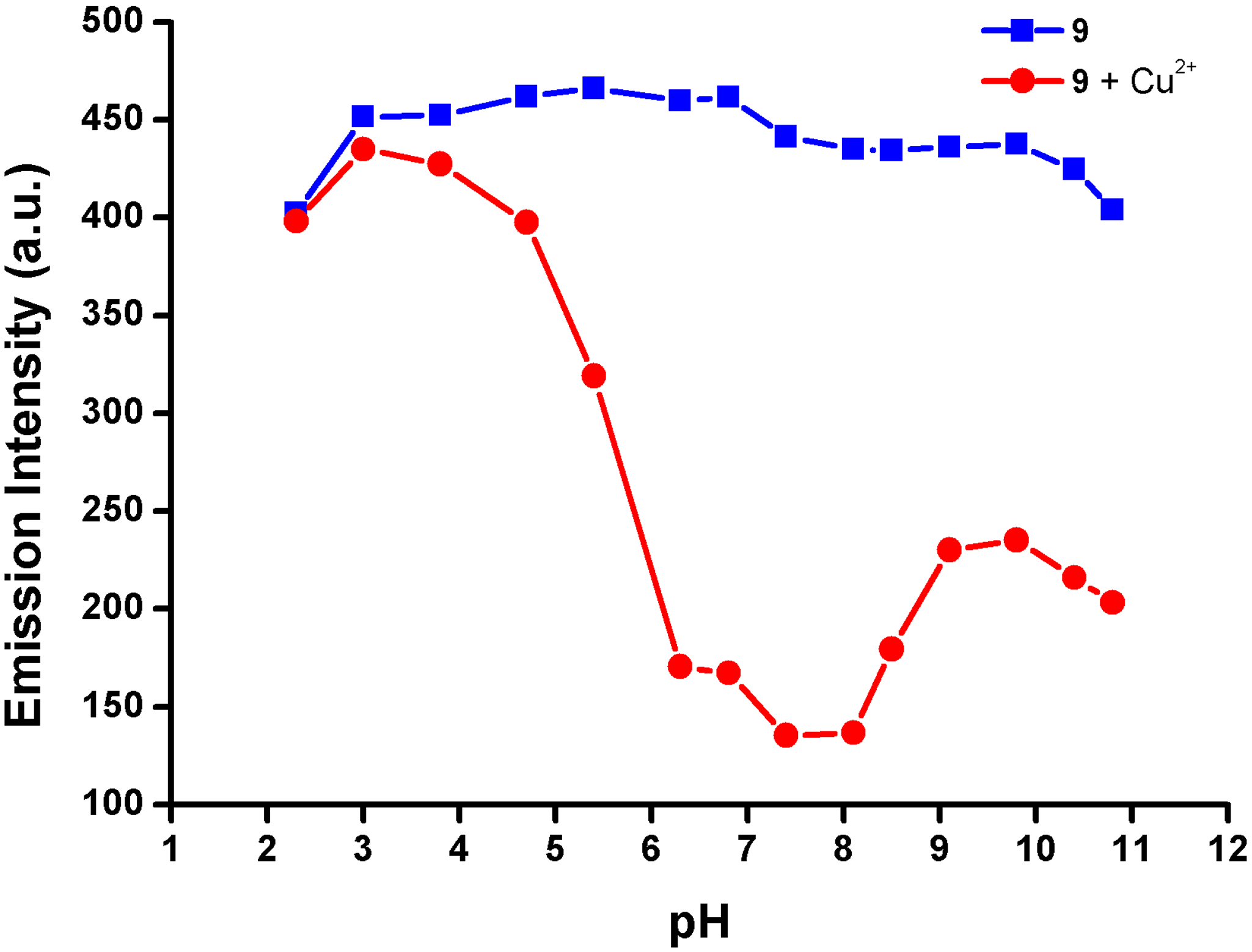

Supplement: File S1 — Contains the files: Text S1. Lippert-Mataga Equation. Text S2. Synthesis of Known Compounds. Figure S1. Stokes shift () of 8 versus orientation polarizability (Δ f ). The red, straight line represents the best linear fit to the 13 data points [coefficient of determination R 2 = 0.560, slope = (4.32±1.07)×103 cm−1, intercept = (4.41±0.26)×103 cm−1]. Figure S2. Stokes shift () of 9 versus orientation polarizability (Δ f ). The red, straight line represents the best linear fit to the 13 data points [coefficient of determination R 2 = 0.392, slope = (3.00±1.02)×103 cm−1, intercept = (4.61±0.25)×103 cm−1]. Figure S3. Stokes shift () of 10 versus orientation polarizability (Δ f ). The red, straight line represents the best linear fit to the 13 data points [coefficient of determination R 2 = 0.562, slope = (4.07±1.00)×103 cm−1, intercept = (4.53±0.25)×103 cm−1]. Figure S4. Fluorescence spectra of 8 (10 µM) in the presence of various metal ions. Experiments were carried out in HEPES buffer (10 mM, pH 7.4) at 25°C and the fluorescence emission spectra were recorded about 5 min after addition of various metal ions (1 equiv.). Figure S5. Fluorescence spectra of 9 (10 µM) in the presence of various metal ions. Experiments were carried out in HEPES buffer (10 mM, pH 7.4) at 25°C and the fluorescence emission spectra were recorded about 5 min after addition of various metal ions (1 equiv.). Figure S6. Fluorescence spectra of 10 (10 µM) in the presence of various metal ions. Experiments were carried out in HEPES buffer (10 mM, pH 7.4) at 25°C and the fluorescence emission spectra were recorded about 5 min after addition of various metal ions (1 equiv.). Figure S7. UV-Vis spectra of 8 (10 µM) in the presence of various metal ions. Experiments were carried out in HEPES buffer (10 mM, pH 7.4) at 25°C and the UV-Vis spectra were recorded about 5 min after addition of various metal ions (1 equiv.). Figure S8. UV-Vis spectra of 9 (10 µM) in the presence of various metal ions. Ex [file pone.0100761.s001.zip › SI/Figure S11.tif]

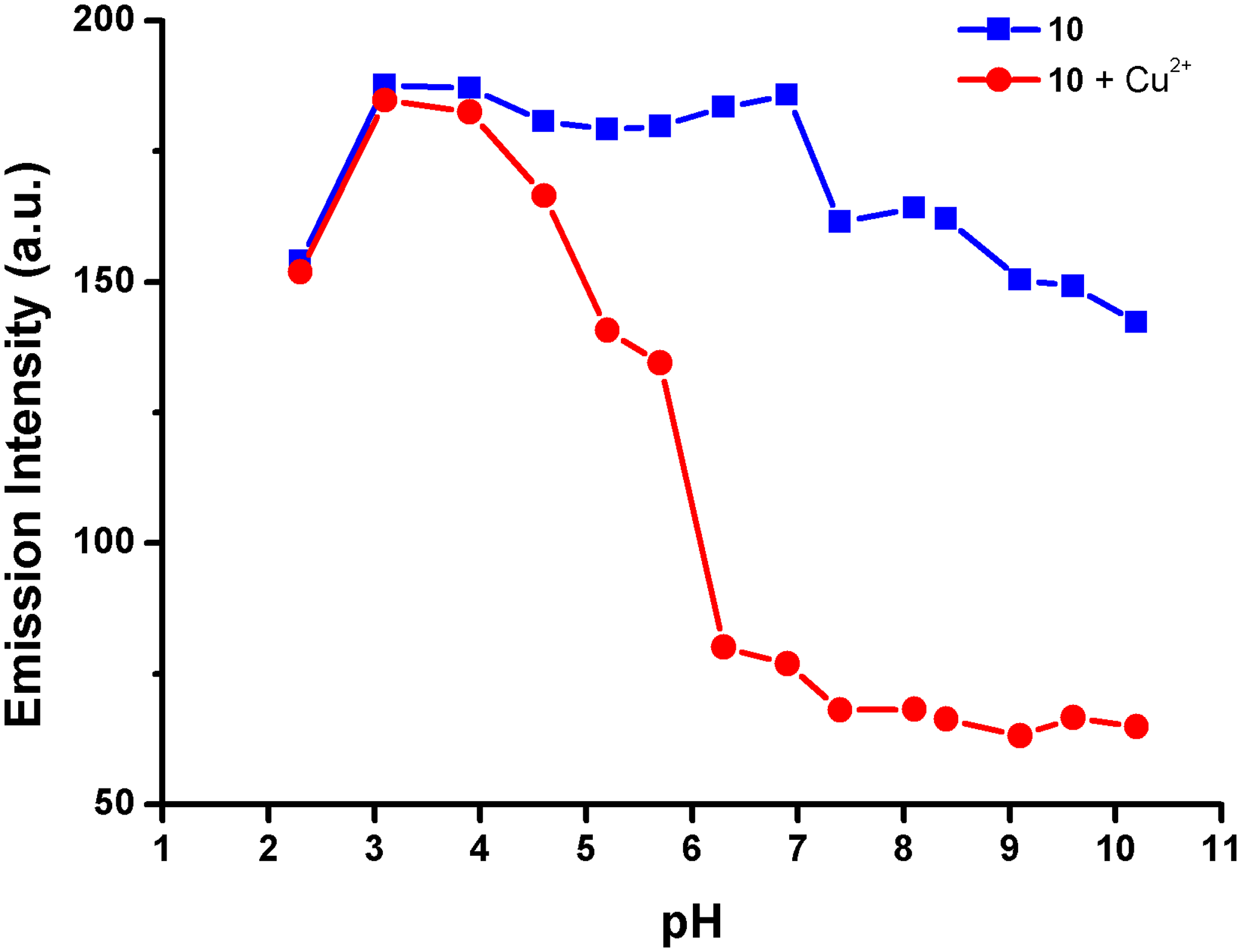

Supplement: File S1 — Contains the files: Text S1. Lippert-Mataga Equation. Text S2. Synthesis of Known Compounds. Figure S1. Stokes shift () of 8 versus orientation polarizability (Δ f ). The red, straight line represents the best linear fit to the 13 data points [coefficient of determination R 2 = 0.560, slope = (4.32±1.07)×103 cm−1, intercept = (4.41±0.26)×103 cm−1]. Figure S2. Stokes shift () of 9 versus orientation polarizability (Δ f ). The red, straight line represents the best linear fit to the 13 data points [coefficient of determination R 2 = 0.392, slope = (3.00±1.02)×103 cm−1, intercept = (4.61±0.25)×103 cm−1]. Figure S3. Stokes shift () of 10 versus orientation polarizability (Δ f ). The red, straight line represents the best linear fit to the 13 data points [coefficient of determination R 2 = 0.562, slope = (4.07±1.00)×103 cm−1, intercept = (4.53±0.25)×103 cm−1]. Figure S4. Fluorescence spectra of 8 (10 µM) in the presence of various metal ions. Experiments were carried out in HEPES buffer (10 mM, pH 7.4) at 25°C and the fluorescence emission spectra were recorded about 5 min after addition of various metal ions (1 equiv.). Figure S5. Fluorescence spectra of 9 (10 µM) in the presence of various metal ions. Experiments were carried out in HEPES buffer (10 mM, pH 7.4) at 25°C and the fluorescence emission spectra were recorded about 5 min after addition of various metal ions (1 equiv.). Figure S6. Fluorescence spectra of 10 (10 µM) in the presence of various metal ions. Experiments were carried out in HEPES buffer (10 mM, pH 7.4) at 25°C and the fluorescence emission spectra were recorded about 5 min after addition of various metal ions (1 equiv.). Figure S7. UV-Vis spectra of 8 (10 µM) in the presence of various metal ions. Experiments were carried out in HEPES buffer (10 mM, pH 7.4) at 25°C and the UV-Vis spectra were recorded about 5 min after addition of various metal ions (1 equiv.). Figure S8. UV-Vis spectra of 9 (10 µM) in the presence of various metal ions. Ex [file pone.0100761.s001.zip › SI/Figure S12.tif]

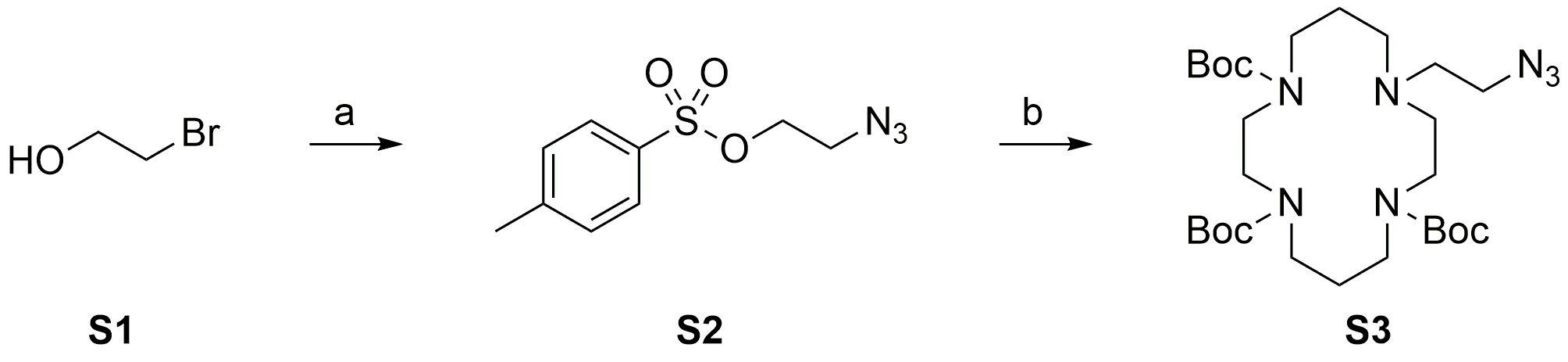

Supplement: File S1 — Contains the files: Text S1. Lippert-Mataga Equation. Text S2. Synthesis of Known Compounds. Figure S1. Stokes shift () of 8 versus orientation polarizability (Δ f ). The red, straight line represents the best linear fit to the 13 data points [coefficient of determination R 2 = 0.560, slope = (4.32±1.07)×103 cm−1, intercept = (4.41±0.26)×103 cm−1]. Figure S2. Stokes shift () of 9 versus orientation polarizability (Δ f ). The red, straight line represents the best linear fit to the 13 data points [coefficient of determination R 2 = 0.392, slope = (3.00±1.02)×103 cm−1, intercept = (4.61±0.25)×103 cm−1]. Figure S3. Stokes shift () of 10 versus orientation polarizability (Δ f ). The red, straight line represents the best linear fit to the 13 data points [coefficient of determination R 2 = 0.562, slope = (4.07±1.00)×103 cm−1, intercept = (4.53±0.25)×103 cm−1]. Figure S4. Fluorescence spectra of 8 (10 µM) in the presence of various metal ions. Experiments were carried out in HEPES buffer (10 mM, pH 7.4) at 25°C and the fluorescence emission spectra were recorded about 5 min after addition of various metal ions (1 equiv.). Figure S5. Fluorescence spectra of 9 (10 µM) in the presence of various metal ions. Experiments were carried out in HEPES buffer (10 mM, pH 7.4) at 25°C and the fluorescence emission spectra were recorded about 5 min after addition of various metal ions (1 equiv.). Figure S6. Fluorescence spectra of 10 (10 µM) in the presence of various metal ions. Experiments were carried out in HEPES buffer (10 mM, pH 7.4) at 25°C and the fluorescence emission spectra were recorded about 5 min after addition of various metal ions (1 equiv.). Figure S7. UV-Vis spectra of 8 (10 µM) in the presence of various metal ions. Experiments were carried out in HEPES buffer (10 mM, pH 7.4) at 25°C and the UV-Vis spectra were recorded about 5 min after addition of various metal ions (1 equiv.). Figure S8. UV-Vis spectra of 9 (10 µM) in the presence of various metal ions. Ex [file pone.0100761.s001.zip › SI/Figure S13.tif]

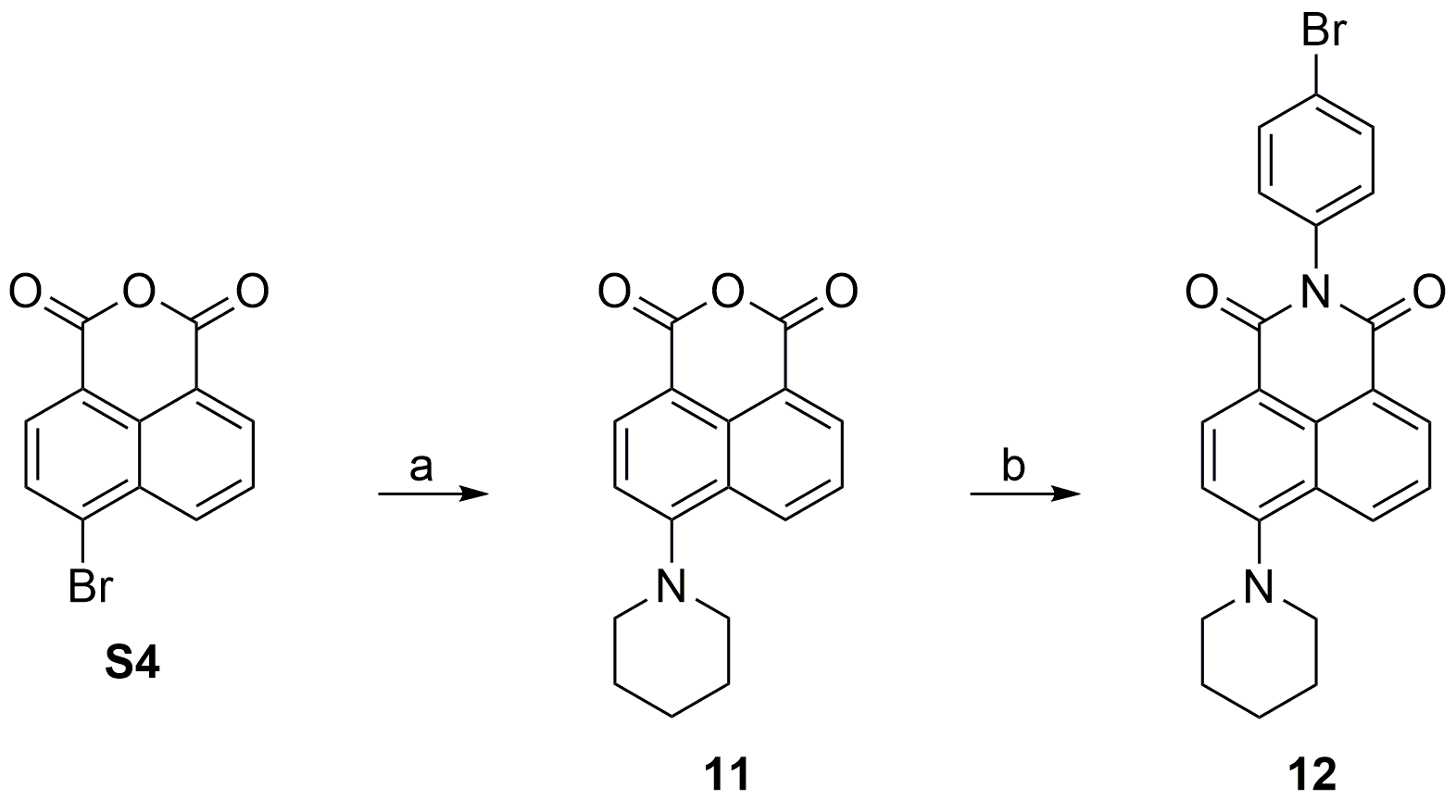

Supplement: File S1 — Contains the files: Text S1. Lippert-Mataga Equation. Text S2. Synthesis of Known Compounds. Figure S1. Stokes shift () of 8 versus orientation polarizability (Δ f ). The red, straight line represents the best linear fit to the 13 data points [coefficient of determination R 2 = 0.560, slope = (4.32±1.07)×103 cm−1, intercept = (4.41±0.26)×103 cm−1]. Figure S2. Stokes shift () of 9 versus orientation polarizability (Δ f ). The red, straight line represents the best linear fit to the 13 data points [coefficient of determination R 2 = 0.392, slope = (3.00±1.02)×103 cm−1, intercept = (4.61±0.25)×103 cm−1]. Figure S3. Stokes shift () of 10 versus orientation polarizability (Δ f ). The red, straight line represents the best linear fit to the 13 data points [coefficient of determination R 2 = 0.562, slope = (4.07±1.00)×103 cm−1, intercept = (4.53±0.25)×103 cm−1]. Figure S4. Fluorescence spectra of 8 (10 µM) in the presence of various metal ions. Experiments were carried out in HEPES buffer (10 mM, pH 7.4) at 25°C and the fluorescence emission spectra were recorded about 5 min after addition of various metal ions (1 equiv.). Figure S5. Fluorescence spectra of 9 (10 µM) in the presence of various metal ions. Experiments were carried out in HEPES buffer (10 mM, pH 7.4) at 25°C and the fluorescence emission spectra were recorded about 5 min after addition of various metal ions (1 equiv.). Figure S6. Fluorescence spectra of 10 (10 µM) in the presence of various metal ions. Experiments were carried out in HEPES buffer (10 mM, pH 7.4) at 25°C and the fluorescence emission spectra were recorded about 5 min after addition of various metal ions (1 equiv.). Figure S7. UV-Vis spectra of 8 (10 µM) in the presence of various metal ions. Experiments were carried out in HEPES buffer (10 mM, pH 7.4) at 25°C and the UV-Vis spectra were recorded about 5 min after addition of various metal ions (1 equiv.). Figure S8. UV-Vis spectra of 9 (10 µM) in the presence of various metal ions. Ex [file pone.0100761.s001.zip › SI/Figure S14.tif]

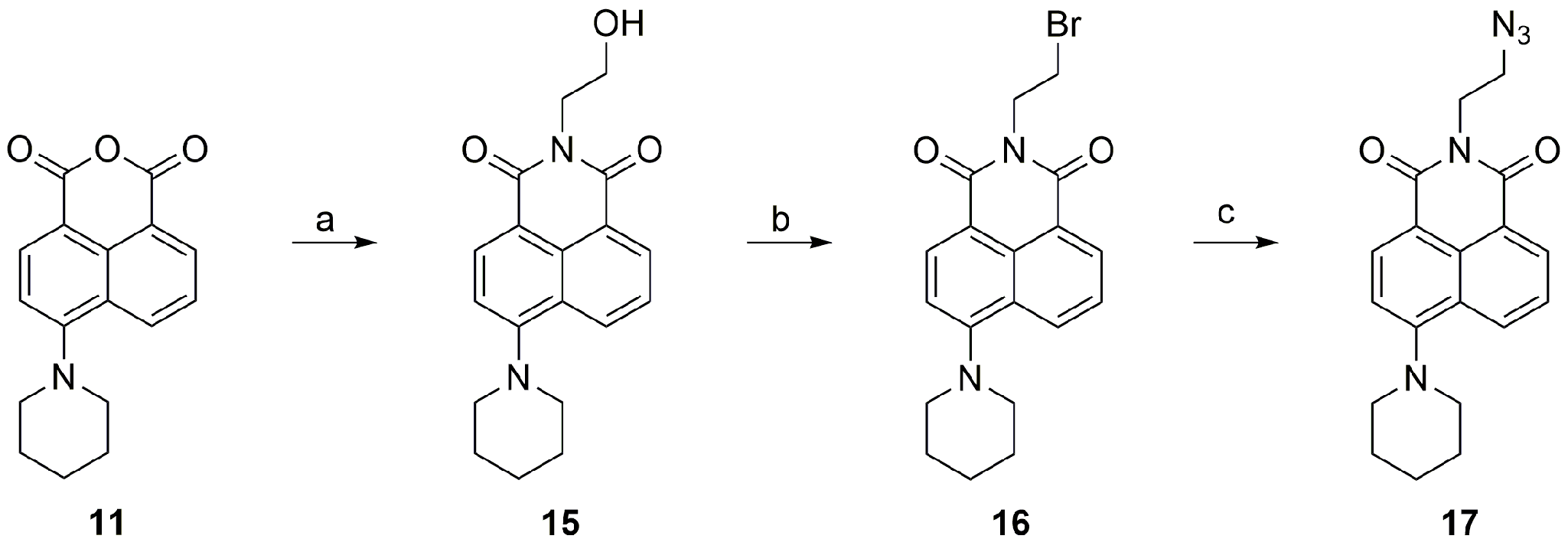

Supplement: File S1 — Contains the files: Text S1. Lippert-Mataga Equation. Text S2. Synthesis of Known Compounds. Figure S1. Stokes shift () of 8 versus orientation polarizability (Δ f ). The red, straight line represents the best linear fit to the 13 data points [coefficient of determination R 2 = 0.560, slope = (4.32±1.07)×103 cm−1, intercept = (4.41±0.26)×103 cm−1]. Figure S2. Stokes shift () of 9 versus orientation polarizability (Δ f ). The red, straight line represents the best linear fit to the 13 data points [coefficient of determination R 2 = 0.392, slope = (3.00±1.02)×103 cm−1, intercept = (4.61±0.25)×103 cm−1]. Figure S3. Stokes shift () of 10 versus orientation polarizability (Δ f ). The red, straight line represents the best linear fit to the 13 data points [coefficient of determination R 2 = 0.562, slope = (4.07±1.00)×103 cm−1, intercept = (4.53±0.25)×103 cm−1]. Figure S4. Fluorescence spectra of 8 (10 µM) in the presence of various metal ions. Experiments were carried out in HEPES buffer (10 mM, pH 7.4) at 25°C and the fluorescence emission spectra were recorded about 5 min after addition of various metal ions (1 equiv.). Figure S5. Fluorescence spectra of 9 (10 µM) in the presence of various metal ions. Experiments were carried out in HEPES buffer (10 mM, pH 7.4) at 25°C and the fluorescence emission spectra were recorded about 5 min after addition of various metal ions (1 equiv.). Figure S6. Fluorescence spectra of 10 (10 µM) in the presence of various metal ions. Experiments were carried out in HEPES buffer (10 mM, pH 7.4) at 25°C and the fluorescence emission spectra were recorded about 5 min after addition of various metal ions (1 equiv.). Figure S7. UV-Vis spectra of 8 (10 µM) in the presence of various metal ions. Experiments were carried out in HEPES buffer (10 mM, pH 7.4) at 25°C and the UV-Vis spectra were recorded about 5 min after addition of various metal ions (1 equiv.). Figure S8. UV-Vis spectra of 9 (10 µM) in the presence of various metal ions. Ex [file pone.0100761.s001.zip › SI/Figure S15.tif]

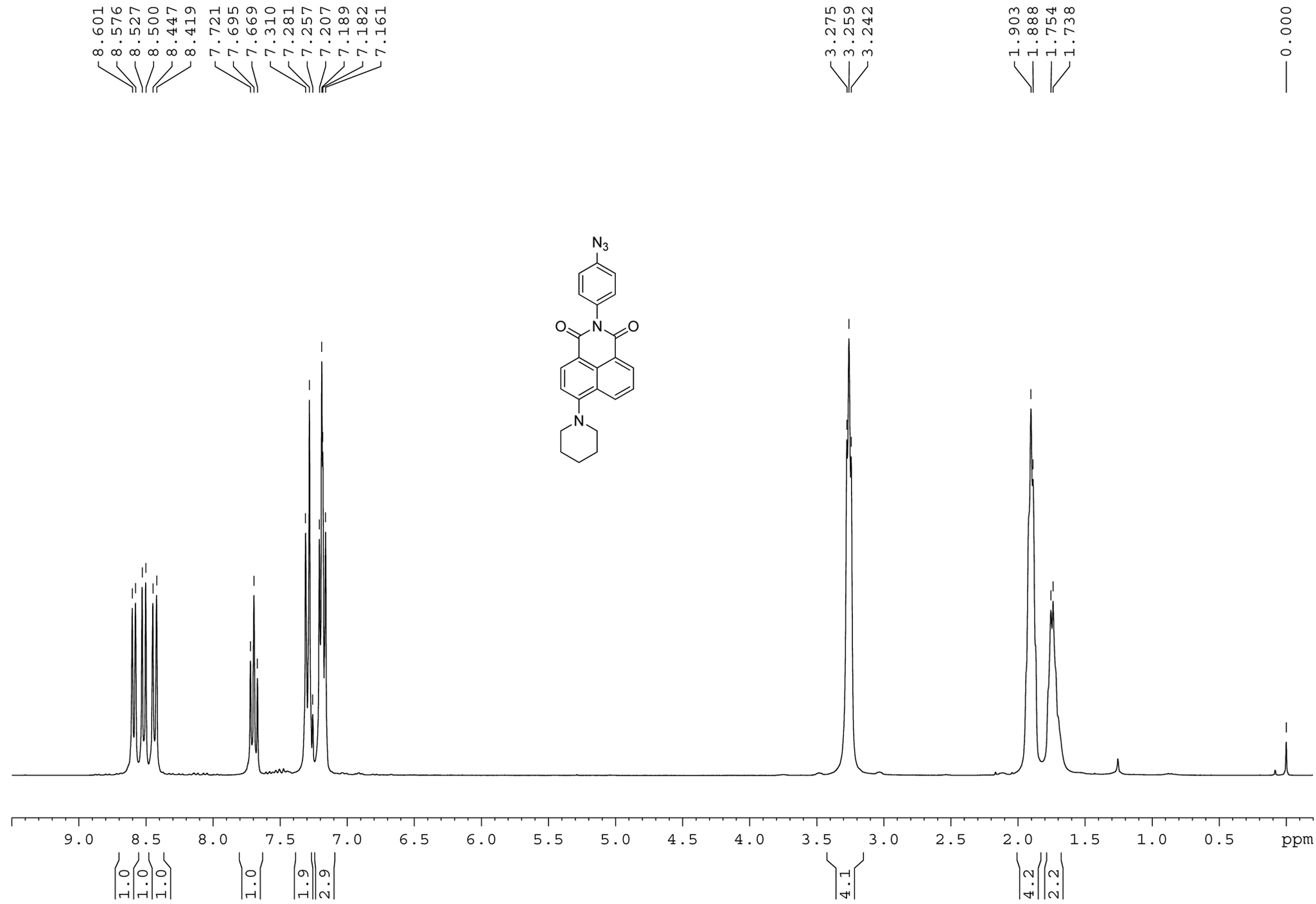

Supplement: File S1 — Contains the files: Text S1. Lippert-Mataga Equation. Text S2. Synthesis of Known Compounds. Figure S1. Stokes shift () of 8 versus orientation polarizability (Δ f ). The red, straight line represents the best linear fit to the 13 data points [coefficient of determination R 2 = 0.560, slope = (4.32±1.07)×103 cm−1, intercept = (4.41±0.26)×103 cm−1]. Figure S2. Stokes shift () of 9 versus orientation polarizability (Δ f ). The red, straight line represents the best linear fit to the 13 data points [coefficient of determination R 2 = 0.392, slope = (3.00±1.02)×103 cm−1, intercept = (4.61±0.25)×103 cm−1]. Figure S3. Stokes shift () of 10 versus orientation polarizability (Δ f ). The red, straight line represents the best linear fit to the 13 data points [coefficient of determination R 2 = 0.562, slope = (4.07±1.00)×103 cm−1, intercept = (4.53±0.25)×103 cm−1]. Figure S4. Fluorescence spectra of 8 (10 µM) in the presence of various metal ions. Experiments were carried out in HEPES buffer (10 mM, pH 7.4) at 25°C and the fluorescence emission spectra were recorded about 5 min after addition of various metal ions (1 equiv.). Figure S5. Fluorescence spectra of 9 (10 µM) in the presence of various metal ions. Experiments were carried out in HEPES buffer (10 mM, pH 7.4) at 25°C and the fluorescence emission spectra were recorded about 5 min after addition of various metal ions (1 equiv.). Figure S6. Fluorescence spectra of 10 (10 µM) in the presence of various metal ions. Experiments were carried out in HEPES buffer (10 mM, pH 7.4) at 25°C and the fluorescence emission spectra were recorded about 5 min after addition of various metal ions (1 equiv.). Figure S7. UV-Vis spectra of 8 (10 µM) in the presence of various metal ions. Experiments were carried out in HEPES buffer (10 mM, pH 7.4) at 25°C and the UV-Vis spectra were recorded about 5 min after addition of various metal ions (1 equiv.). Figure S8. UV-Vis spectra of 9 (10 µM) in the presence of various metal ions. Ex [file pone.0100761.s001.zip › SI/Figure S16.tif]

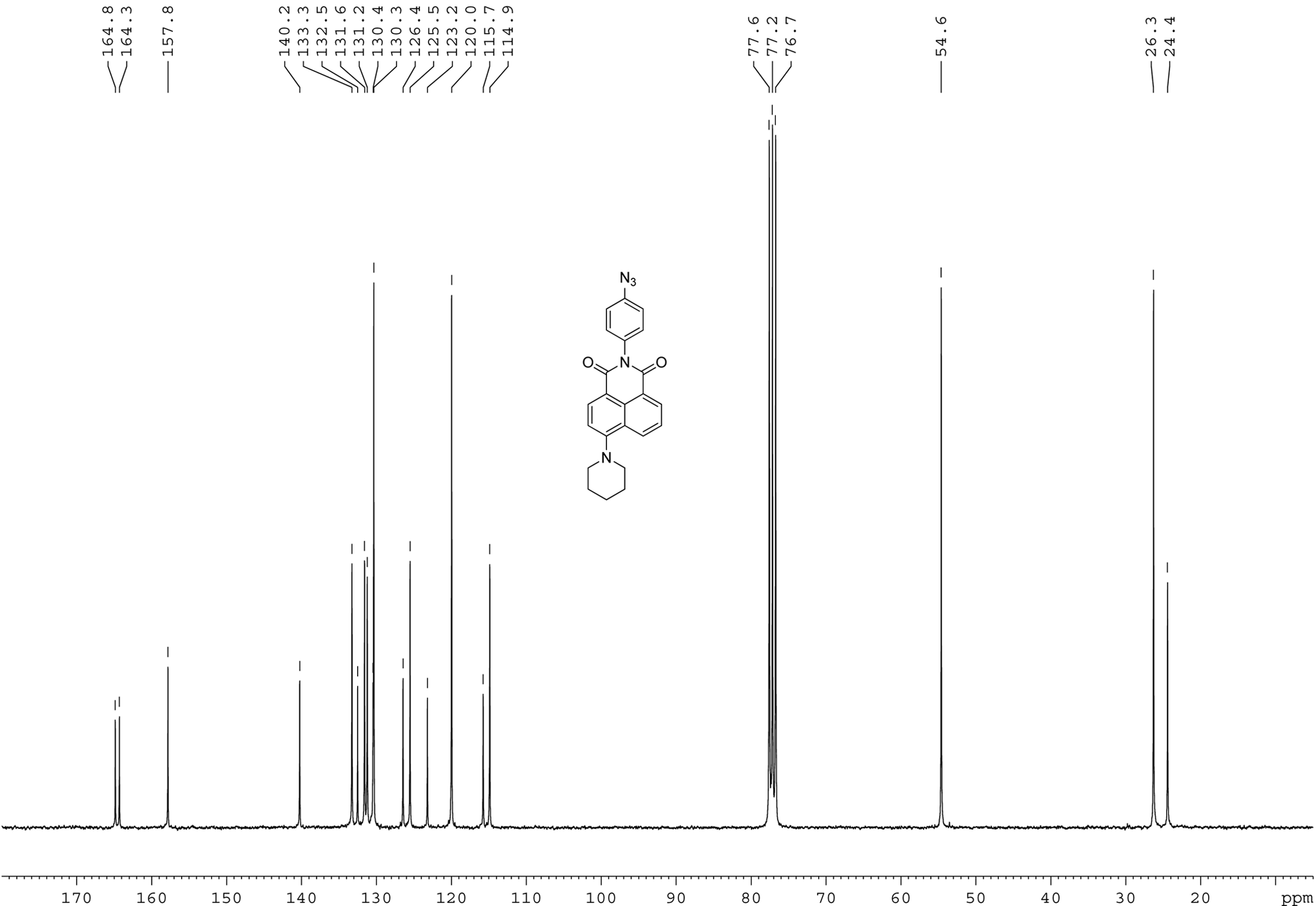

Supplement: File S1 — Contains the files: Text S1. Lippert-Mataga Equation. Text S2. Synthesis of Known Compounds. Figure S1. Stokes shift () of 8 versus orientation polarizability (Δ f ). The red, straight line represents the best linear fit to the 13 data points [coefficient of determination R 2 = 0.560, slope = (4.32±1.07)×103 cm−1, intercept = (4.41±0.26)×103 cm−1]. Figure S2. Stokes shift () of 9 versus orientation polarizability (Δ f ). The red, straight line represents the best linear fit to the 13 data points [coefficient of determination R 2 = 0.392, slope = (3.00±1.02)×103 cm−1, intercept = (4.61±0.25)×103 cm−1]. Figure S3. Stokes shift () of 10 versus orientation polarizability (Δ f ). The red, straight line represents the best linear fit to the 13 data points [coefficient of determination R 2 = 0.562, slope = (4.07±1.00)×103 cm−1, intercept = (4.53±0.25)×103 cm−1]. Figure S4. Fluorescence spectra of 8 (10 µM) in the presence of various metal ions. Experiments were carried out in HEPES buffer (10 mM, pH 7.4) at 25°C and the fluorescence emission spectra were recorded about 5 min after addition of various metal ions (1 equiv.). Figure S5. Fluorescence spectra of 9 (10 µM) in the presence of various metal ions. Experiments were carried out in HEPES buffer (10 mM, pH 7.4) at 25°C and the fluorescence emission spectra were recorded about 5 min after addition of various metal ions (1 equiv.). Figure S6. Fluorescence spectra of 10 (10 µM) in the presence of various metal ions. Experiments were carried out in HEPES buffer (10 mM, pH 7.4) at 25°C and the fluorescence emission spectra were recorded about 5 min after addition of various metal ions (1 equiv.). Figure S7. UV-Vis spectra of 8 (10 µM) in the presence of various metal ions. Experiments were carried out in HEPES buffer (10 mM, pH 7.4) at 25°C and the UV-Vis spectra were recorded about 5 min after addition of various metal ions (1 equiv.). Figure S8. UV-Vis spectra of 9 (10 µM) in the presence of various metal ions. Ex [file pone.0100761.s001.zip › SI/Figure S17.tif]

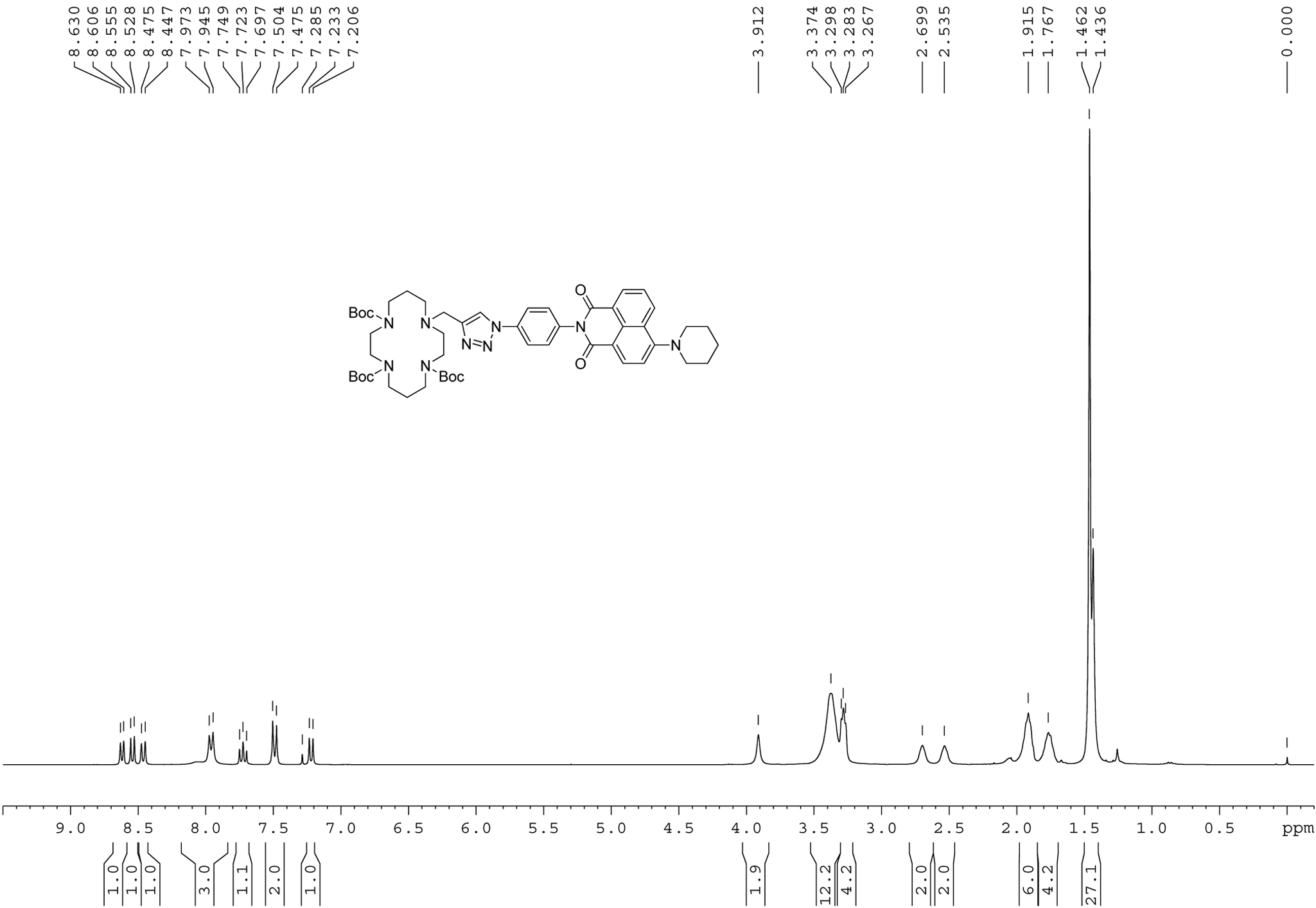

Supplement: File S1 — Contains the files: Text S1. Lippert-Mataga Equation. Text S2. Synthesis of Known Compounds. Figure S1. Stokes shift () of 8 versus orientation polarizability (Δ f ). The red, straight line represents the best linear fit to the 13 data points [coefficient of determination R 2 = 0.560, slope = (4.32±1.07)×103 cm−1, intercept = (4.41±0.26)×103 cm−1]. Figure S2. Stokes shift () of 9 versus orientation polarizability (Δ f ). The red, straight line represents the best linear fit to the 13 data points [coefficient of determination R 2 = 0.392, slope = (3.00±1.02)×103 cm−1, intercept = (4.61±0.25)×103 cm−1]. Figure S3. Stokes shift () of 10 versus orientation polarizability (Δ f ). The red, straight line represents the best linear fit to the 13 data points [coefficient of determination R 2 = 0.562, slope = (4.07±1.00)×103 cm−1, intercept = (4.53±0.25)×103 cm−1]. Figure S4. Fluorescence spectra of 8 (10 µM) in the presence of various metal ions. Experiments were carried out in HEPES buffer (10 mM, pH 7.4) at 25°C and the fluorescence emission spectra were recorded about 5 min after addition of various metal ions (1 equiv.). Figure S5. Fluorescence spectra of 9 (10 µM) in the presence of various metal ions. Experiments were carried out in HEPES buffer (10 mM, pH 7.4) at 25°C and the fluorescence emission spectra were recorded about 5 min after addition of various metal ions (1 equiv.). Figure S6. Fluorescence spectra of 10 (10 µM) in the presence of various metal ions. Experiments were carried out in HEPES buffer (10 mM, pH 7.4) at 25°C and the fluorescence emission spectra were recorded about 5 min after addition of various metal ions (1 equiv.). Figure S7. UV-Vis spectra of 8 (10 µM) in the presence of various metal ions. Experiments were carried out in HEPES buffer (10 mM, pH 7.4) at 25°C and the UV-Vis spectra were recorded about 5 min after addition of various metal ions (1 equiv.). Figure S8. UV-Vis spectra of 9 (10 µM) in the presence of various metal ions. Ex [file pone.0100761.s001.zip › SI/Figure S18.tif]

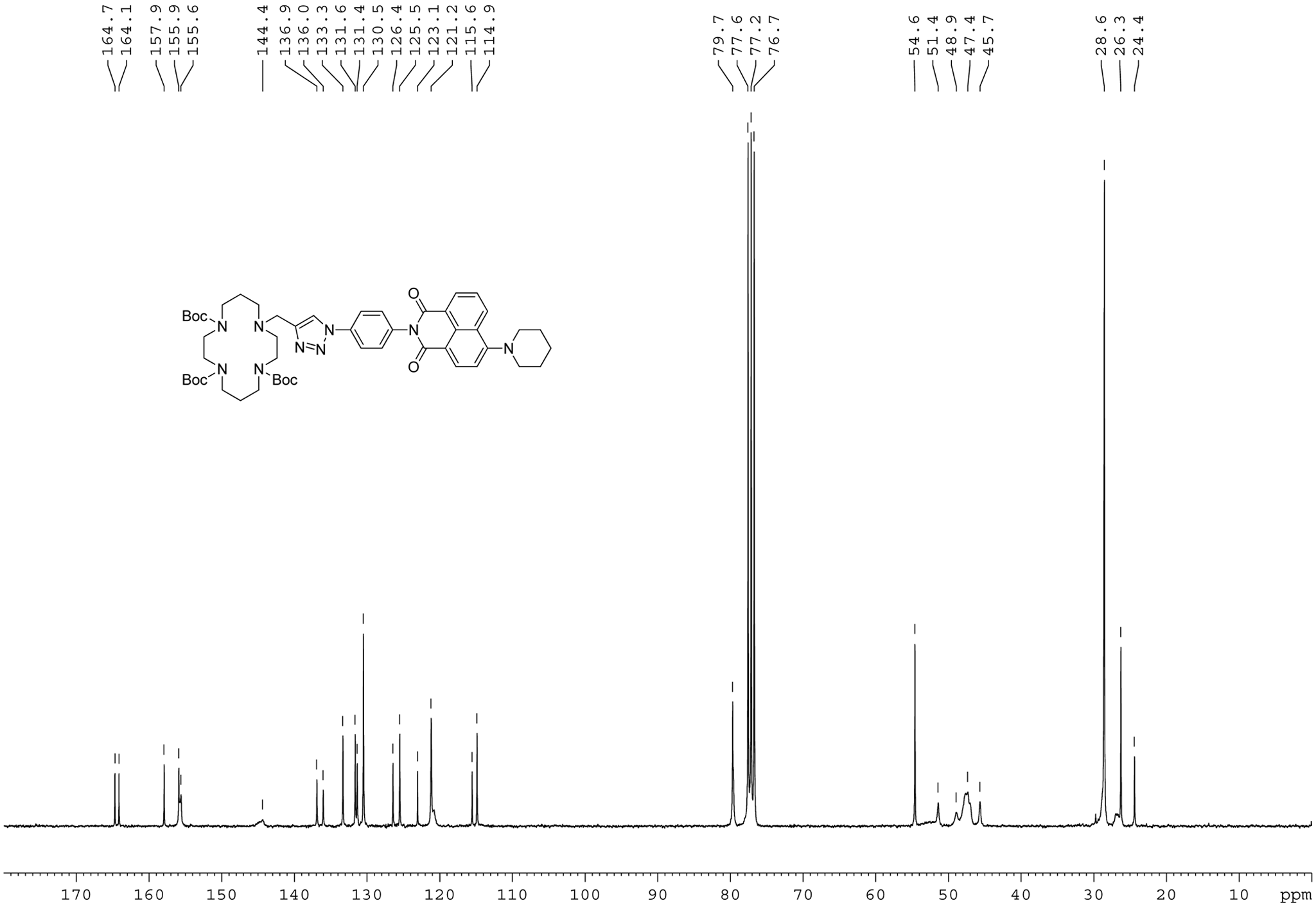

Supplement: File S1 — Contains the files: Text S1. Lippert-Mataga Equation. Text S2. Synthesis of Known Compounds. Figure S1. Stokes shift () of 8 versus orientation polarizability (Δ f ). The red, straight line represents the best linear fit to the 13 data points [coefficient of determination R 2 = 0.560, slope = (4.32±1.07)×103 cm−1, intercept = (4.41±0.26)×103 cm−1]. Figure S2. Stokes shift () of 9 versus orientation polarizability (Δ f ). The red, straight line represents the best linear fit to the 13 data points [coefficient of determination R 2 = 0.392, slope = (3.00±1.02)×103 cm−1, intercept = (4.61±0.25)×103 cm−1]. Figure S3. Stokes shift () of 10 versus orientation polarizability (Δ f ). The red, straight line represents the best linear fit to the 13 data points [coefficient of determination R 2 = 0.562, slope = (4.07±1.00)×103 cm−1, intercept = (4.53±0.25)×103 cm−1]. Figure S4. Fluorescence spectra of 8 (10 µM) in the presence of various metal ions. Experiments were carried out in HEPES buffer (10 mM, pH 7.4) at 25°C and the fluorescence emission spectra were recorded about 5 min after addition of various metal ions (1 equiv.). Figure S5. Fluorescence spectra of 9 (10 µM) in the presence of various metal ions. Experiments were carried out in HEPES buffer (10 mM, pH 7.4) at 25°C and the fluorescence emission spectra were recorded about 5 min after addition of various metal ions (1 equiv.). Figure S6. Fluorescence spectra of 10 (10 µM) in the presence of various metal ions. Experiments were carried out in HEPES buffer (10 mM, pH 7.4) at 25°C and the fluorescence emission spectra were recorded about 5 min after addition of various metal ions (1 equiv.). Figure S7. UV-Vis spectra of 8 (10 µM) in the presence of various metal ions. Experiments were carried out in HEPES buffer (10 mM, pH 7.4) at 25°C and the UV-Vis spectra were recorded about 5 min after addition of various metal ions (1 equiv.). Figure S8. UV-Vis spectra of 9 (10 µM) in the presence of various metal ions. Ex [file pone.0100761.s001.zip › SI/Figure S19.tif]

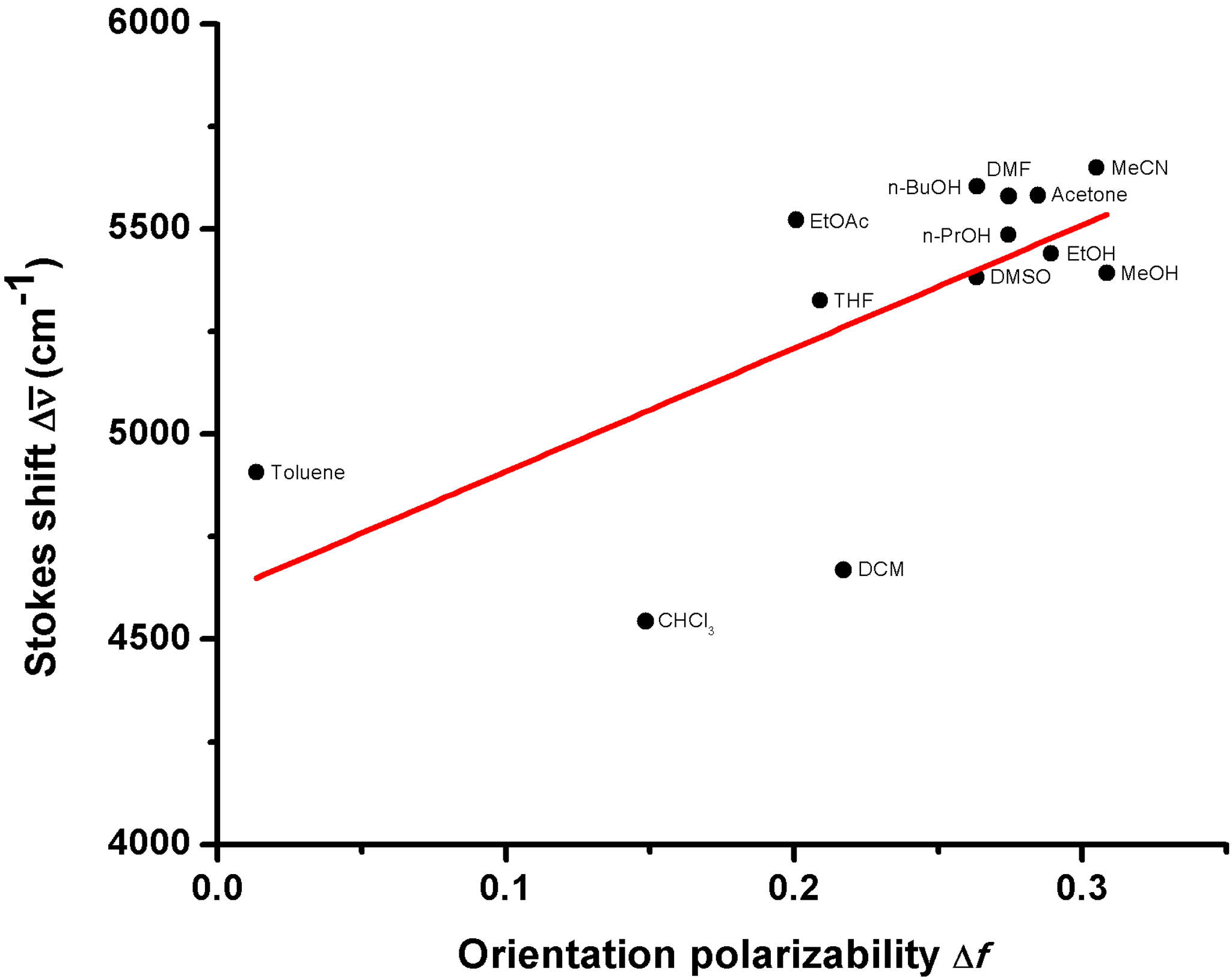

Supplement: File S1 — Contains the files: Text S1. Lippert-Mataga Equation. Text S2. Synthesis of Known Compounds. Figure S1. Stokes shift () of 8 versus orientation polarizability (Δ f ). The red, straight line represents the best linear fit to the 13 data points [coefficient of determination R 2 = 0.560, slope = (4.32±1.07)×103 cm−1, intercept = (4.41±0.26)×103 cm−1]. Figure S2. Stokes shift () of 9 versus orientation polarizability (Δ f ). The red, straight line represents the best linear fit to the 13 data points [coefficient of determination R 2 = 0.392, slope = (3.00±1.02)×103 cm−1, intercept = (4.61±0.25)×103 cm−1]. Figure S3. Stokes shift () of 10 versus orientation polarizability (Δ f ). The red, straight line represents the best linear fit to the 13 data points [coefficient of determination R 2 = 0.562, slope = (4.07±1.00)×103 cm−1, intercept = (4.53±0.25)×103 cm−1]. Figure S4. Fluorescence spectra of 8 (10 µM) in the presence of various metal ions. Experiments were carried out in HEPES buffer (10 mM, pH 7.4) at 25°C and the fluorescence emission spectra were recorded about 5 min after addition of various metal ions (1 equiv.). Figure S5. Fluorescence spectra of 9 (10 µM) in the presence of various metal ions. Experiments were carried out in HEPES buffer (10 mM, pH 7.4) at 25°C and the fluorescence emission spectra were recorded about 5 min after addition of various metal ions (1 equiv.). Figure S6. Fluorescence spectra of 10 (10 µM) in the presence of various metal ions. Experiments were carried out in HEPES buffer (10 mM, pH 7.4) at 25°C and the fluorescence emission spectra were recorded about 5 min after addition of various metal ions (1 equiv.). Figure S7. UV-Vis spectra of 8 (10 µM) in the presence of various metal ions. Experiments were carried out in HEPES buffer (10 mM, pH 7.4) at 25°C and the UV-Vis spectra were recorded about 5 min after addition of various metal ions (1 equiv.). Figure S8. UV-Vis spectra of 9 (10 µM) in the presence of various metal ions. Ex [file pone.0100761.s001.zip › SI/Figure S2.tif]

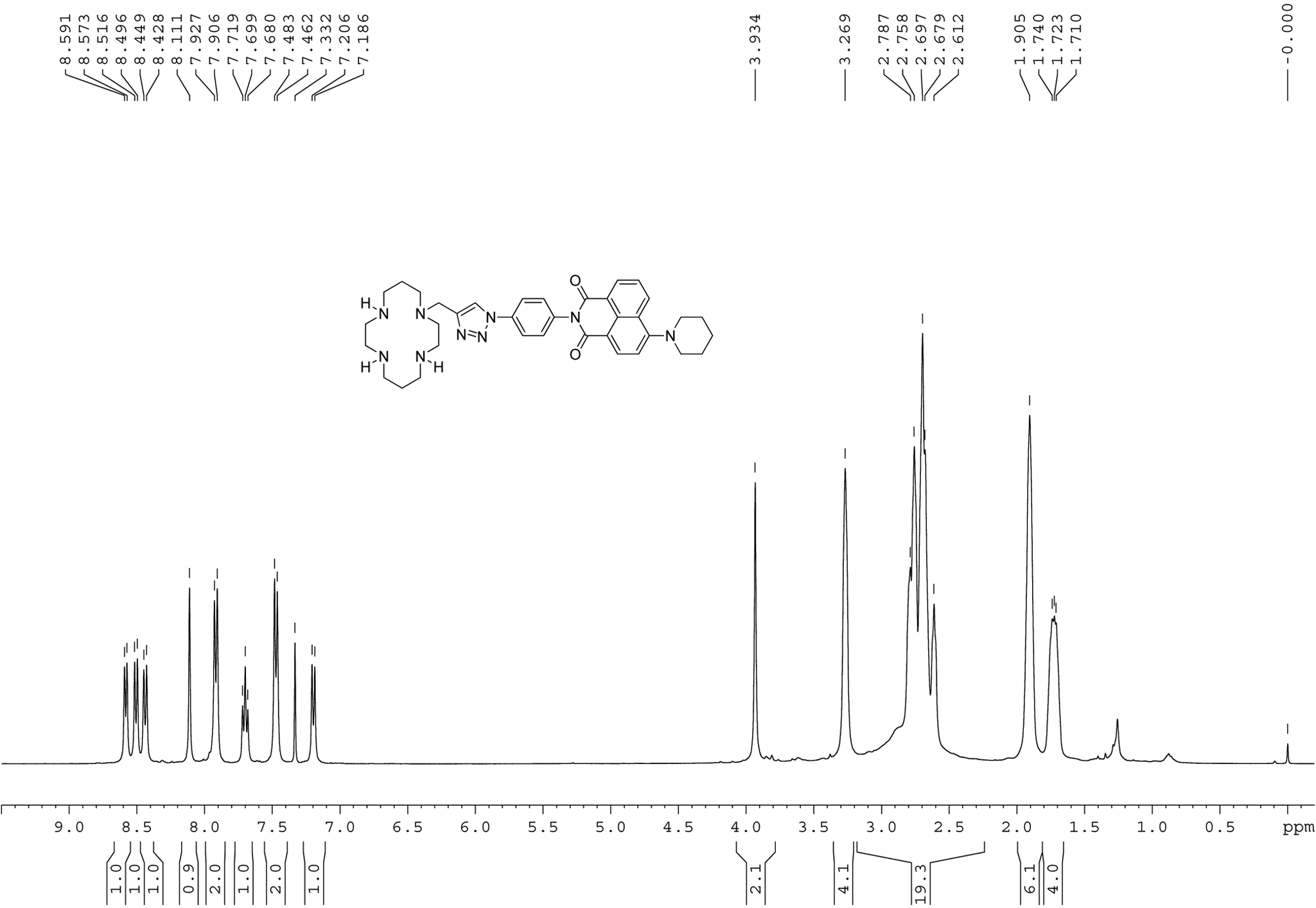

Supplement: File S1 — Contains the files: Text S1. Lippert-Mataga Equation. Text S2. Synthesis of Known Compounds. Figure S1. Stokes shift () of 8 versus orientation polarizability (Δ f ). The red, straight line represents the best linear fit to the 13 data points [coefficient of determination R 2 = 0.560, slope = (4.32±1.07)×103 cm−1, intercept = (4.41±0.26)×103 cm−1]. Figure S2. Stokes shift () of 9 versus orientation polarizability (Δ f ). The red, straight line represents the best linear fit to the 13 data points [coefficient of determination R 2 = 0.392, slope = (3.00±1.02)×103 cm−1, intercept = (4.61±0.25)×103 cm−1]. Figure S3. Stokes shift () of 10 versus orientation polarizability (Δ f ). The red, straight line represents the best linear fit to the 13 data points [coefficient of determination R 2 = 0.562, slope = (4.07±1.00)×103 cm−1, intercept = (4.53±0.25)×103 cm−1]. Figure S4. Fluorescence spectra of 8 (10 µM) in the presence of various metal ions. Experiments were carried out in HEPES buffer (10 mM, pH 7.4) at 25°C and the fluorescence emission spectra were recorded about 5 min after addition of various metal ions (1 equiv.). Figure S5. Fluorescence spectra of 9 (10 µM) in the presence of various metal ions. Experiments were carried out in HEPES buffer (10 mM, pH 7.4) at 25°C and the fluorescence emission spectra were recorded about 5 min after addition of various metal ions (1 equiv.). Figure S6. Fluorescence spectra of 10 (10 µM) in the presence of various metal ions. Experiments were carried out in HEPES buffer (10 mM, pH 7.4) at 25°C and the fluorescence emission spectra were recorded about 5 min after addition of various metal ions (1 equiv.). Figure S7. UV-Vis spectra of 8 (10 µM) in the presence of various metal ions. Experiments were carried out in HEPES buffer (10 mM, pH 7.4) at 25°C and the UV-Vis spectra were recorded about 5 min after addition of various metal ions (1 equiv.). Figure S8. UV-Vis spectra of 9 (10 µM) in the presence of various metal ions. Ex [file pone.0100761.s001.zip › SI/Figure S20.tif]

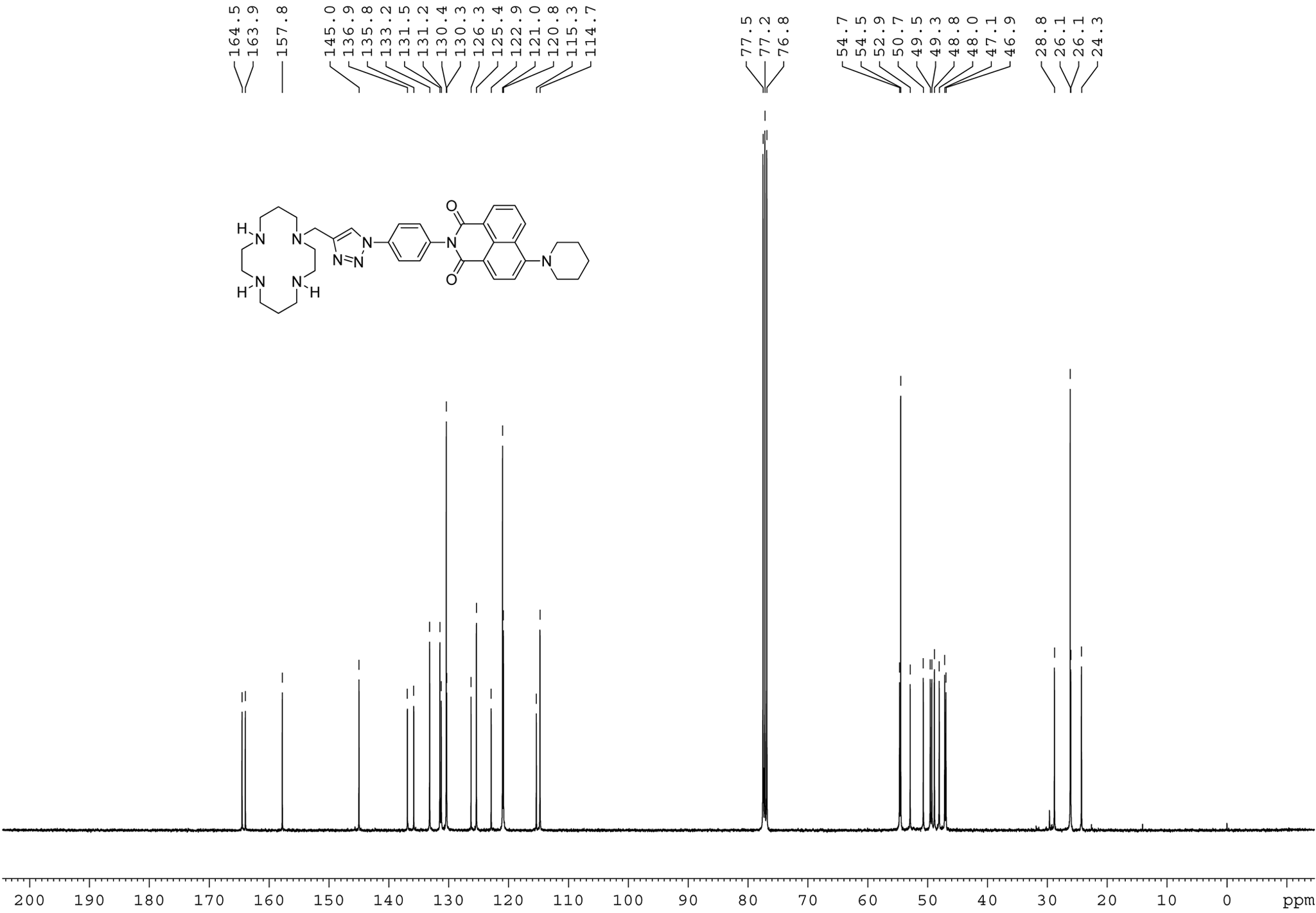

Supplement: File S1 — Contains the files: Text S1. Lippert-Mataga Equation. Text S2. Synthesis of Known Compounds. Figure S1. Stokes shift () of 8 versus orientation polarizability (Δ f ). The red, straight line represents the best linear fit to the 13 data points [coefficient of determination R 2 = 0.560, slope = (4.32±1.07)×103 cm−1, intercept = (4.41±0.26)×103 cm−1]. Figure S2. Stokes shift () of 9 versus orientation polarizability (Δ f ). The red, straight line represents the best linear fit to the 13 data points [coefficient of determination R 2 = 0.392, slope = (3.00±1.02)×103 cm−1, intercept = (4.61±0.25)×103 cm−1]. Figure S3. Stokes shift () of 10 versus orientation polarizability (Δ f ). The red, straight line represents the best linear fit to the 13 data points [coefficient of determination R 2 = 0.562, slope = (4.07±1.00)×103 cm−1, intercept = (4.53±0.25)×103 cm−1]. Figure S4. Fluorescence spectra of 8 (10 µM) in the presence of various metal ions. Experiments were carried out in HEPES buffer (10 mM, pH 7.4) at 25°C and the fluorescence emission spectra were recorded about 5 min after addition of various metal ions (1 equiv.). Figure S5. Fluorescence spectra of 9 (10 µM) in the presence of various metal ions. Experiments were carried out in HEPES buffer (10 mM, pH 7.4) at 25°C and the fluorescence emission spectra were recorded about 5 min after addition of various metal ions (1 equiv.). Figure S6. Fluorescence spectra of 10 (10 µM) in the presence of various metal ions. Experiments were carried out in HEPES buffer (10 mM, pH 7.4) at 25°C and the fluorescence emission spectra were recorded about 5 min after addition of various metal ions (1 equiv.). Figure S7. UV-Vis spectra of 8 (10 µM) in the presence of various metal ions. Experiments were carried out in HEPES buffer (10 mM, pH 7.4) at 25°C and the UV-Vis spectra were recorded about 5 min after addition of various metal ions (1 equiv.). Figure S8. UV-Vis spectra of 9 (10 µM) in the presence of various metal ions. Ex [file pone.0100761.s001.zip › SI/Figure S21.tif]

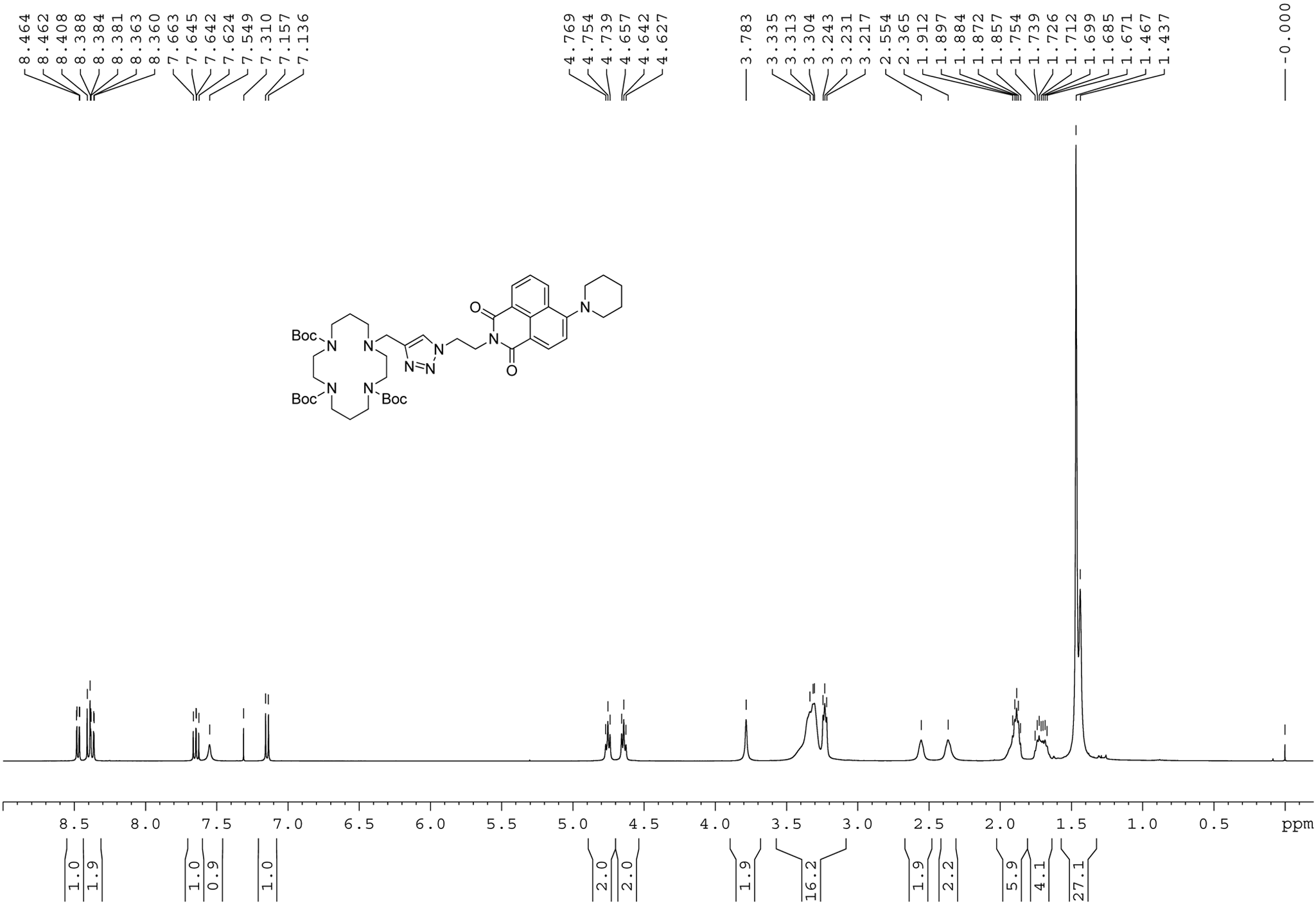

Supplement: File S1 — Contains the files: Text S1. Lippert-Mataga Equation. Text S2. Synthesis of Known Compounds. Figure S1. Stokes shift () of 8 versus orientation polarizability (Δ f ). The red, straight line represents the best linear fit to the 13 data points [coefficient of determination R 2 = 0.560, slope = (4.32±1.07)×103 cm−1, intercept = (4.41±0.26)×103 cm−1]. Figure S2. Stokes shift () of 9 versus orientation polarizability (Δ f ). The red, straight line represents the best linear fit to the 13 data points [coefficient of determination R 2 = 0.392, slope = (3.00±1.02)×103 cm−1, intercept = (4.61±0.25)×103 cm−1]. Figure S3. Stokes shift () of 10 versus orientation polarizability (Δ f ). The red, straight line represents the best linear fit to the 13 data points [coefficient of determination R 2 = 0.562, slope = (4.07±1.00)×103 cm−1, intercept = (4.53±0.25)×103 cm−1]. Figure S4. Fluorescence spectra of 8 (10 µM) in the presence of various metal ions. Experiments were carried out in HEPES buffer (10 mM, pH 7.4) at 25°C and the fluorescence emission spectra were recorded about 5 min after addition of various metal ions (1 equiv.). Figure S5. Fluorescence spectra of 9 (10 µM) in the presence of various metal ions. Experiments were carried out in HEPES buffer (10 mM, pH 7.4) at 25°C and the fluorescence emission spectra were recorded about 5 min after addition of various metal ions (1 equiv.). Figure S6. Fluorescence spectra of 10 (10 µM) in the presence of various metal ions. Experiments were carried out in HEPES buffer (10 mM, pH 7.4) at 25°C and the fluorescence emission spectra were recorded about 5 min after addition of various metal ions (1 equiv.). Figure S7. UV-Vis spectra of 8 (10 µM) in the presence of various metal ions. Experiments were carried out in HEPES buffer (10 mM, pH 7.4) at 25°C and the UV-Vis spectra were recorded about 5 min after addition of various metal ions (1 equiv.). Figure S8. UV-Vis spectra of 9 (10 µM) in the presence of various metal ions. Ex [file pone.0100761.s001.zip › SI/Figure S22.tif]

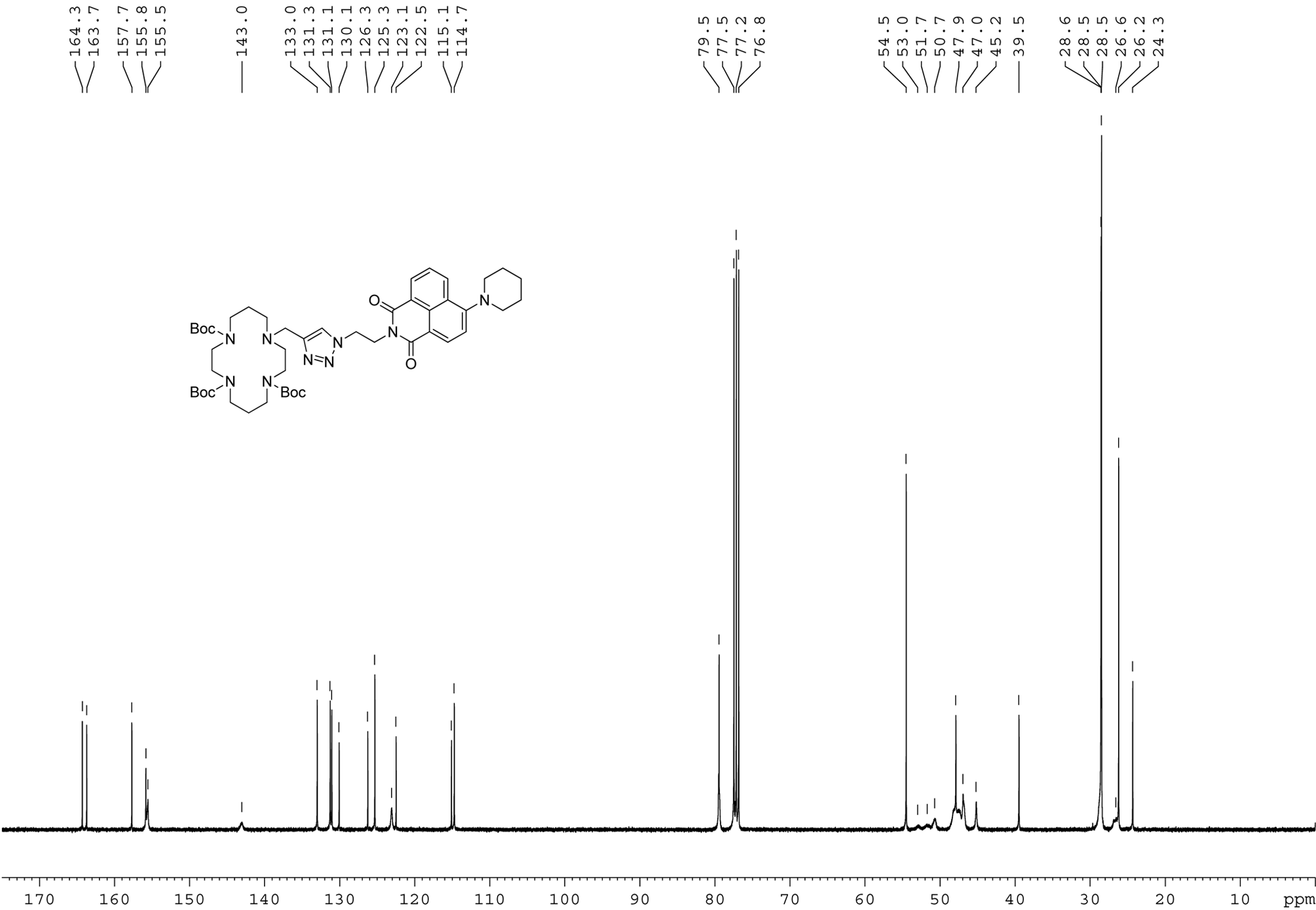

Supplement: File S1 — Contains the files: Text S1. Lippert-Mataga Equation. Text S2. Synthesis of Known Compounds. Figure S1. Stokes shift () of 8 versus orientation polarizability (Δ f ). The red, straight line represents the best linear fit to the 13 data points [coefficient of determination R 2 = 0.560, slope = (4.32±1.07)×103 cm−1, intercept = (4.41±0.26)×103 cm−1]. Figure S2. Stokes shift () of 9 versus orientation polarizability (Δ f ). The red, straight line represents the best linear fit to the 13 data points [coefficient of determination R 2 = 0.392, slope = (3.00±1.02)×103 cm−1, intercept = (4.61±0.25)×103 cm−1]. Figure S3. Stokes shift () of 10 versus orientation polarizability (Δ f ). The red, straight line represents the best linear fit to the 13 data points [coefficient of determination R 2 = 0.562, slope = (4.07±1.00)×103 cm−1, intercept = (4.53±0.25)×103 cm−1]. Figure S4. Fluorescence spectra of 8 (10 µM) in the presence of various metal ions. Experiments were carried out in HEPES buffer (10 mM, pH 7.4) at 25°C and the fluorescence emission spectra were recorded about 5 min after addition of various metal ions (1 equiv.). Figure S5. Fluorescence spectra of 9 (10 µM) in the presence of various metal ions. Experiments were carried out in HEPES buffer (10 mM, pH 7.4) at 25°C and the fluorescence emission spectra were recorded about 5 min after addition of various metal ions (1 equiv.). Figure S6. Fluorescence spectra of 10 (10 µM) in the presence of various metal ions. Experiments were carried out in HEPES buffer (10 mM, pH 7.4) at 25°C and the fluorescence emission spectra were recorded about 5 min after addition of various metal ions (1 equiv.). Figure S7. UV-Vis spectra of 8 (10 µM) in the presence of various metal ions. Experiments were carried out in HEPES buffer (10 mM, pH 7.4) at 25°C and the UV-Vis spectra were recorded about 5 min after addition of various metal ions (1 equiv.). Figure S8. UV-Vis spectra of 9 (10 µM) in the presence of various metal ions. Ex [file pone.0100761.s001.zip › SI/Figure S23.tif]

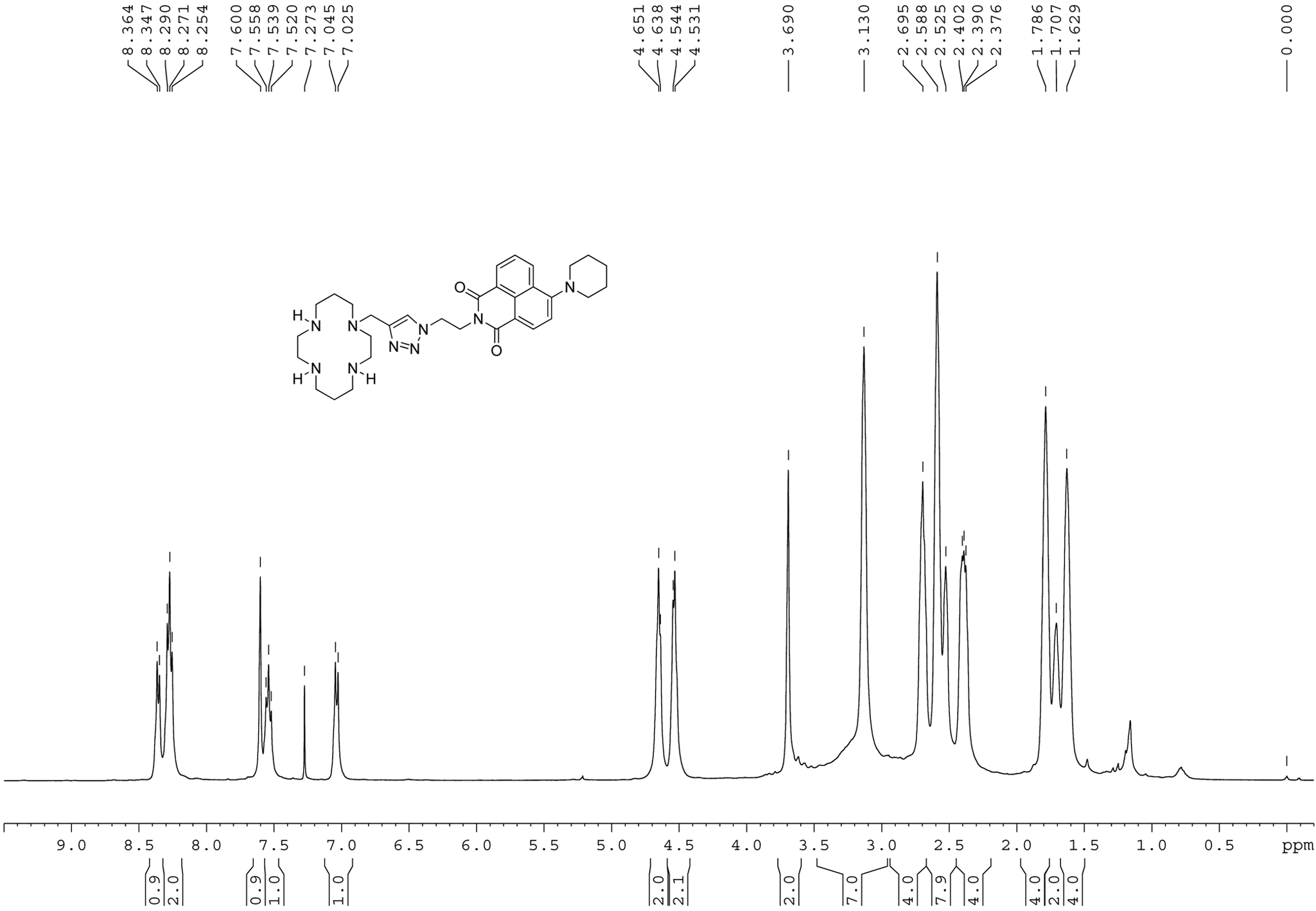

Supplement: File S1 — Contains the files: Text S1. Lippert-Mataga Equation. Text S2. Synthesis of Known Compounds. Figure S1. Stokes shift () of 8 versus orientation polarizability (Δ f ). The red, straight line represents the best linear fit to the 13 data points [coefficient of determination R 2 = 0.560, slope = (4.32±1.07)×103 cm−1, intercept = (4.41±0.26)×103 cm−1]. Figure S2. Stokes shift () of 9 versus orientation polarizability (Δ f ). The red, straight line represents the best linear fit to the 13 data points [coefficient of determination R 2 = 0.392, slope = (3.00±1.02)×103 cm−1, intercept = (4.61±0.25)×103 cm−1]. Figure S3. Stokes shift () of 10 versus orientation polarizability (Δ f ). The red, straight line represents the best linear fit to the 13 data points [coefficient of determination R 2 = 0.562, slope = (4.07±1.00)×103 cm−1, intercept = (4.53±0.25)×103 cm−1]. Figure S4. Fluorescence spectra of 8 (10 µM) in the presence of various metal ions. Experiments were carried out in HEPES buffer (10 mM, pH 7.4) at 25°C and the fluorescence emission spectra were recorded about 5 min after addition of various metal ions (1 equiv.). Figure S5. Fluorescence spectra of 9 (10 µM) in the presence of various metal ions. Experiments were carried out in HEPES buffer (10 mM, pH 7.4) at 25°C and the fluorescence emission spectra were recorded about 5 min after addition of various metal ions (1 equiv.). Figure S6. Fluorescence spectra of 10 (10 µM) in the presence of various metal ions. Experiments were carried out in HEPES buffer (10 mM, pH 7.4) at 25°C and the fluorescence emission spectra were recorded about 5 min after addition of various metal ions (1 equiv.). Figure S7. UV-Vis spectra of 8 (10 µM) in the presence of various metal ions. Experiments were carried out in HEPES buffer (10 mM, pH 7.4) at 25°C and the UV-Vis spectra were recorded about 5 min after addition of various metal ions (1 equiv.). Figure S8. UV-Vis spectra of 9 (10 µM) in the presence of various metal ions. Ex [file pone.0100761.s001.zip › SI/Figure S24.tif]

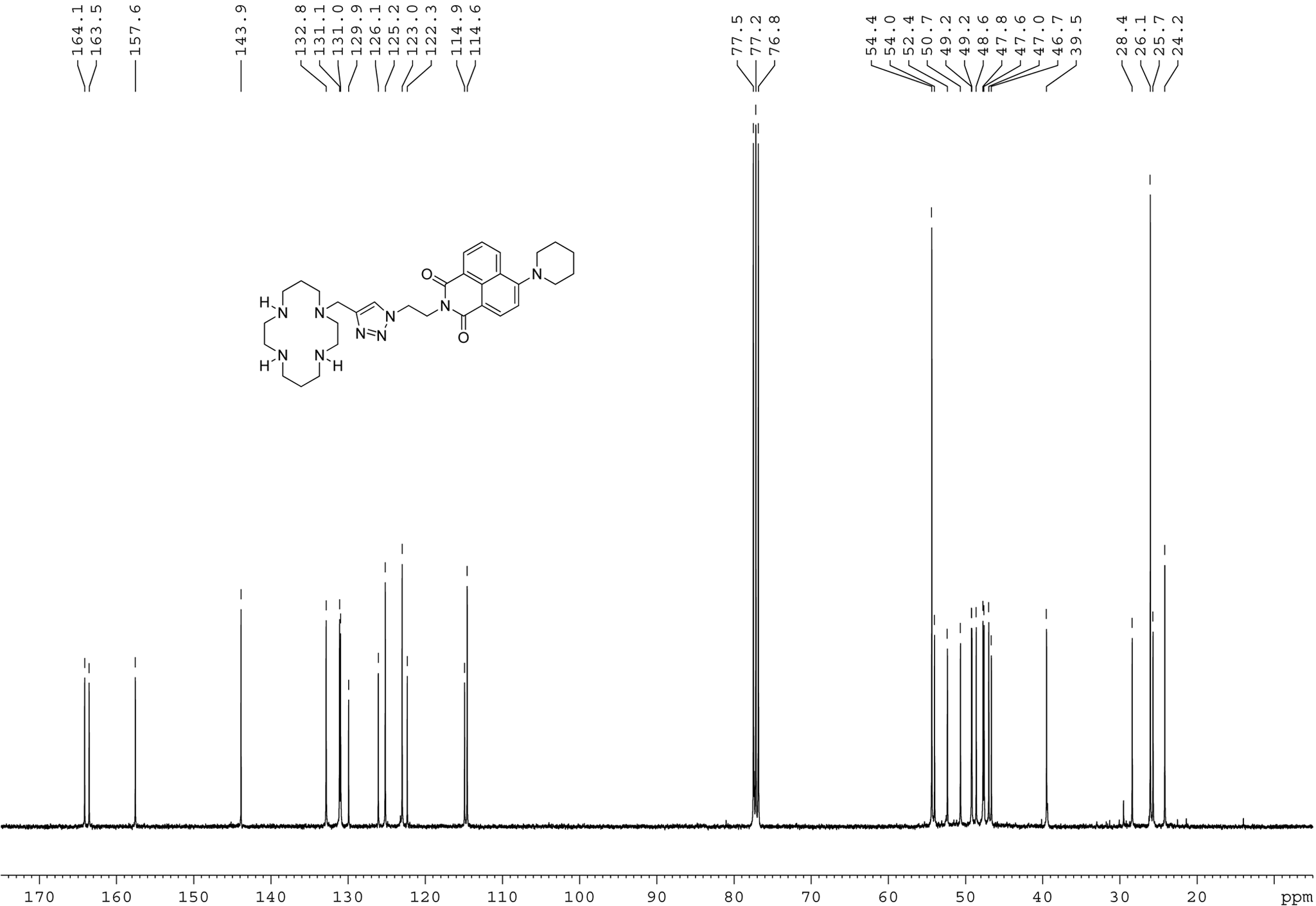

Supplement: File S1 — Contains the files: Text S1. Lippert-Mataga Equation. Text S2. Synthesis of Known Compounds. Figure S1. Stokes shift () of 8 versus orientation polarizability (Δ f ). The red, straight line represents the best linear fit to the 13 data points [coefficient of determination R 2 = 0.560, slope = (4.32±1.07)×103 cm−1, intercept = (4.41±0.26)×103 cm−1]. Figure S2. Stokes shift () of 9 versus orientation polarizability (Δ f ). The red, straight line represents the best linear fit to the 13 data points [coefficient of determination R 2 = 0.392, slope = (3.00±1.02)×103 cm−1, intercept = (4.61±0.25)×103 cm−1]. Figure S3. Stokes shift () of 10 versus orientation polarizability (Δ f ). The red, straight line represents the best linear fit to the 13 data points [coefficient of determination R 2 = 0.562, slope = (4.07±1.00)×103 cm−1, intercept = (4.53±0.25)×103 cm−1]. Figure S4. Fluorescence spectra of 8 (10 µM) in the presence of various metal ions. Experiments were carried out in HEPES buffer (10 mM, pH 7.4) at 25°C and the fluorescence emission spectra were recorded about 5 min after addition of various metal ions (1 equiv.). Figure S5. Fluorescence spectra of 9 (10 µM) in the presence of various metal ions. Experiments were carried out in HEPES buffer (10 mM, pH 7.4) at 25°C and the fluorescence emission spectra were recorded about 5 min after addition of various metal ions (1 equiv.). Figure S6. Fluorescence spectra of 10 (10 µM) in the presence of various metal ions. Experiments were carried out in HEPES buffer (10 mM, pH 7.4) at 25°C and the fluorescence emission spectra were recorded about 5 min after addition of various metal ions (1 equiv.). Figure S7. UV-Vis spectra of 8 (10 µM) in the presence of various metal ions. Experiments were carried out in HEPES buffer (10 mM, pH 7.4) at 25°C and the UV-Vis spectra were recorded about 5 min after addition of various metal ions (1 equiv.). Figure S8. UV-Vis spectra of 9 (10 µM) in the presence of various metal ions. Ex [file pone.0100761.s001.zip › SI/Figure S25.tif]

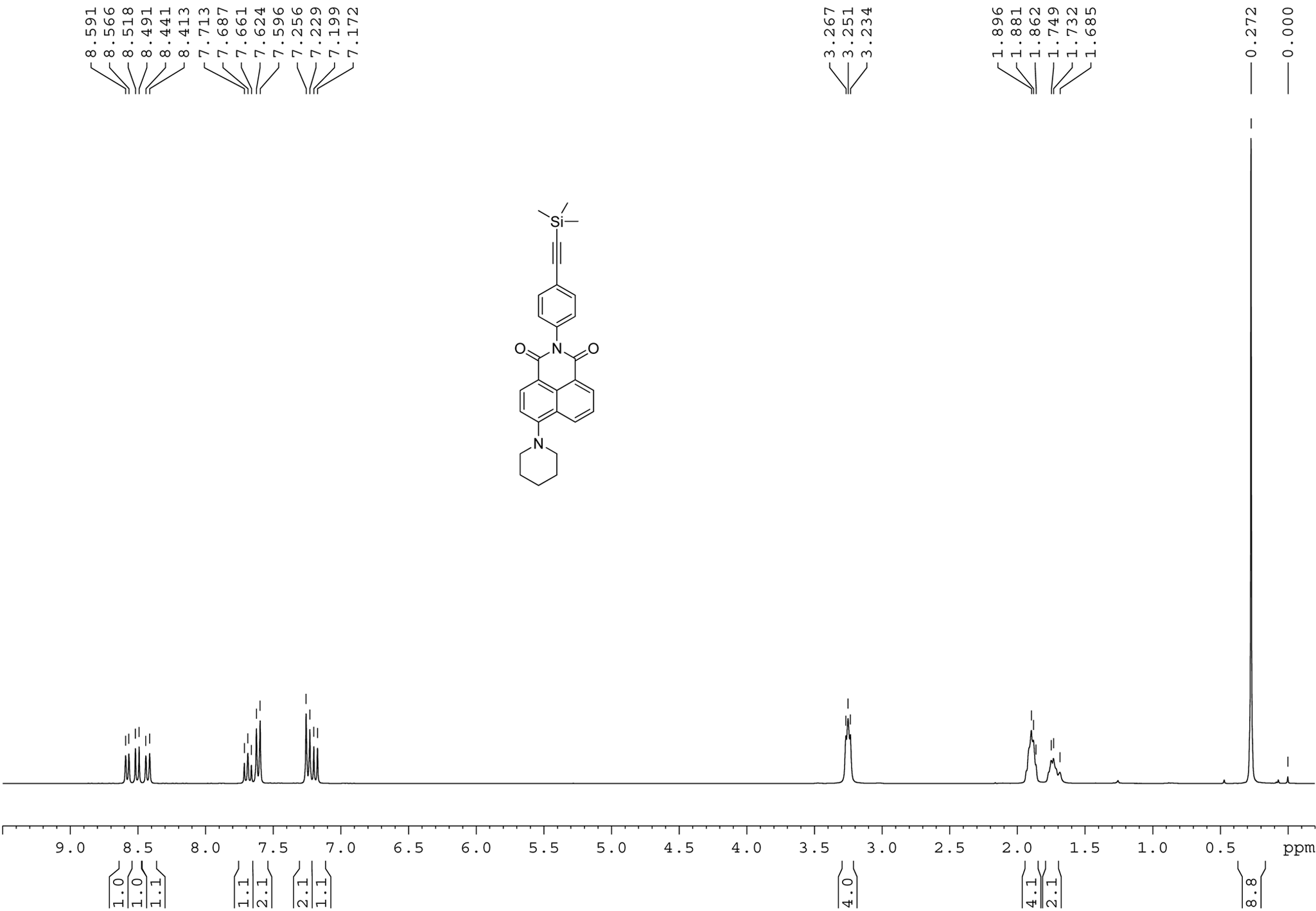

Supplement: File S1 — Contains the files: Text S1. Lippert-Mataga Equation. Text S2. Synthesis of Known Compounds. Figure S1. Stokes shift () of 8 versus orientation polarizability (Δ f ). The red, straight line represents the best linear fit to the 13 data points [coefficient of determination R 2 = 0.560, slope = (4.32±1.07)×103 cm−1, intercept = (4.41±0.26)×103 cm−1]. Figure S2. Stokes shift () of 9 versus orientation polarizability (Δ f ). The red, straight line represents the best linear fit to the 13 data points [coefficient of determination R 2 = 0.392, slope = (3.00±1.02)×103 cm−1, intercept = (4.61±0.25)×103 cm−1]. Figure S3. Stokes shift () of 10 versus orientation polarizability (Δ f ). The red, straight line represents the best linear fit to the 13 data points [coefficient of determination R 2 = 0.562, slope = (4.07±1.00)×103 cm−1, intercept = (4.53±0.25)×103 cm−1]. Figure S4. Fluorescence spectra of 8 (10 µM) in the presence of various metal ions. Experiments were carried out in HEPES buffer (10 mM, pH 7.4) at 25°C and the fluorescence emission spectra were recorded about 5 min after addition of various metal ions (1 equiv.). Figure S5. Fluorescence spectra of 9 (10 µM) in the presence of various metal ions. Experiments were carried out in HEPES buffer (10 mM, pH 7.4) at 25°C and the fluorescence emission spectra were recorded about 5 min after addition of various metal ions (1 equiv.). Figure S6. Fluorescence spectra of 10 (10 µM) in the presence of various metal ions. Experiments were carried out in HEPES buffer (10 mM, pH 7.4) at 25°C and the fluorescence emission spectra were recorded about 5 min after addition of various metal ions (1 equiv.). Figure S7. UV-Vis spectra of 8 (10 µM) in the presence of various metal ions. Experiments were carried out in HEPES buffer (10 mM, pH 7.4) at 25°C and the UV-Vis spectra were recorded about 5 min after addition of various metal ions (1 equiv.). Figure S8. UV-Vis spectra of 9 (10 µM) in the presence of various metal ions. Ex [file pone.0100761.s001.zip › SI/Figure S26.tif]

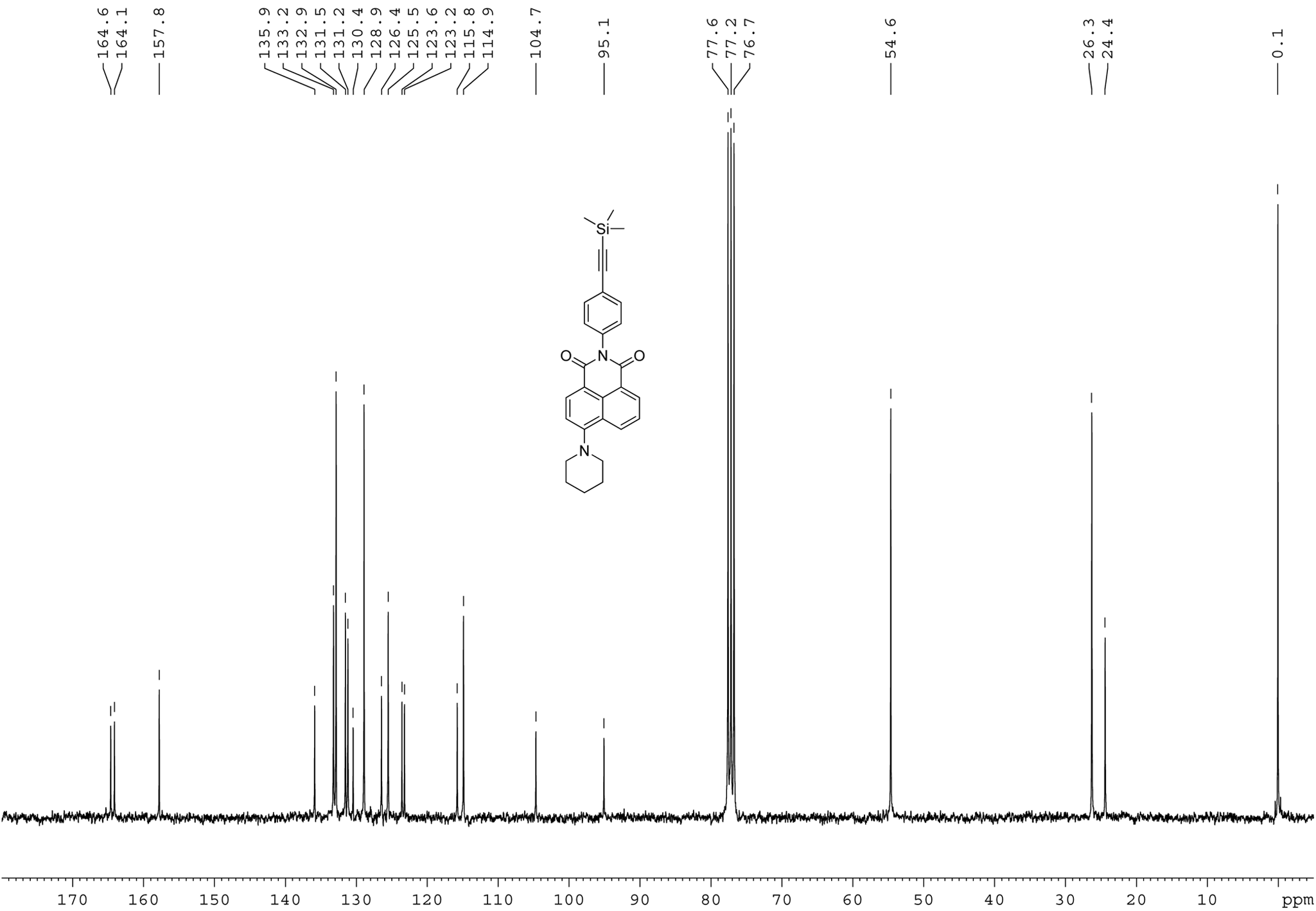

Supplement: File S1 — Contains the files: Text S1. Lippert-Mataga Equation. Text S2. Synthesis of Known Compounds. Figure S1. Stokes shift () of 8 versus orientation polarizability (Δ f ). The red, straight line represents the best linear fit to the 13 data points [coefficient of determination R 2 = 0.560, slope = (4.32±1.07)×103 cm−1, intercept = (4.41±0.26)×103 cm−1]. Figure S2. Stokes shift () of 9 versus orientation polarizability (Δ f ). The red, straight line represents the best linear fit to the 13 data points [coefficient of determination R 2 = 0.392, slope = (3.00±1.02)×103 cm−1, intercept = (4.61±0.25)×103 cm−1]. Figure S3. Stokes shift () of 10 versus orientation polarizability (Δ f ). The red, straight line represents the best linear fit to the 13 data points [coefficient of determination R 2 = 0.562, slope = (4.07±1.00)×103 cm−1, intercept = (4.53±0.25)×103 cm−1]. Figure S4. Fluorescence spectra of 8 (10 µM) in the presence of various metal ions. Experiments were carried out in HEPES buffer (10 mM, pH 7.4) at 25°C and the fluorescence emission spectra were recorded about 5 min after addition of various metal ions (1 equiv.). Figure S5. Fluorescence spectra of 9 (10 µM) in the presence of various metal ions. Experiments were carried out in HEPES buffer (10 mM, pH 7.4) at 25°C and the fluorescence emission spectra were recorded about 5 min after addition of various metal ions (1 equiv.). Figure S6. Fluorescence spectra of 10 (10 µM) in the presence of various metal ions. Experiments were carried out in HEPES buffer (10 mM, pH 7.4) at 25°C and the fluorescence emission spectra were recorded about 5 min after addition of various metal ions (1 equiv.). Figure S7. UV-Vis spectra of 8 (10 µM) in the presence of various metal ions. Experiments were carried out in HEPES buffer (10 mM, pH 7.4) at 25°C and the UV-Vis spectra were recorded about 5 min after addition of various metal ions (1 equiv.). Figure S8. UV-Vis spectra of 9 (10 µM) in the presence of various metal ions. Ex [file pone.0100761.s001.zip › SI/Figure S27.tif]

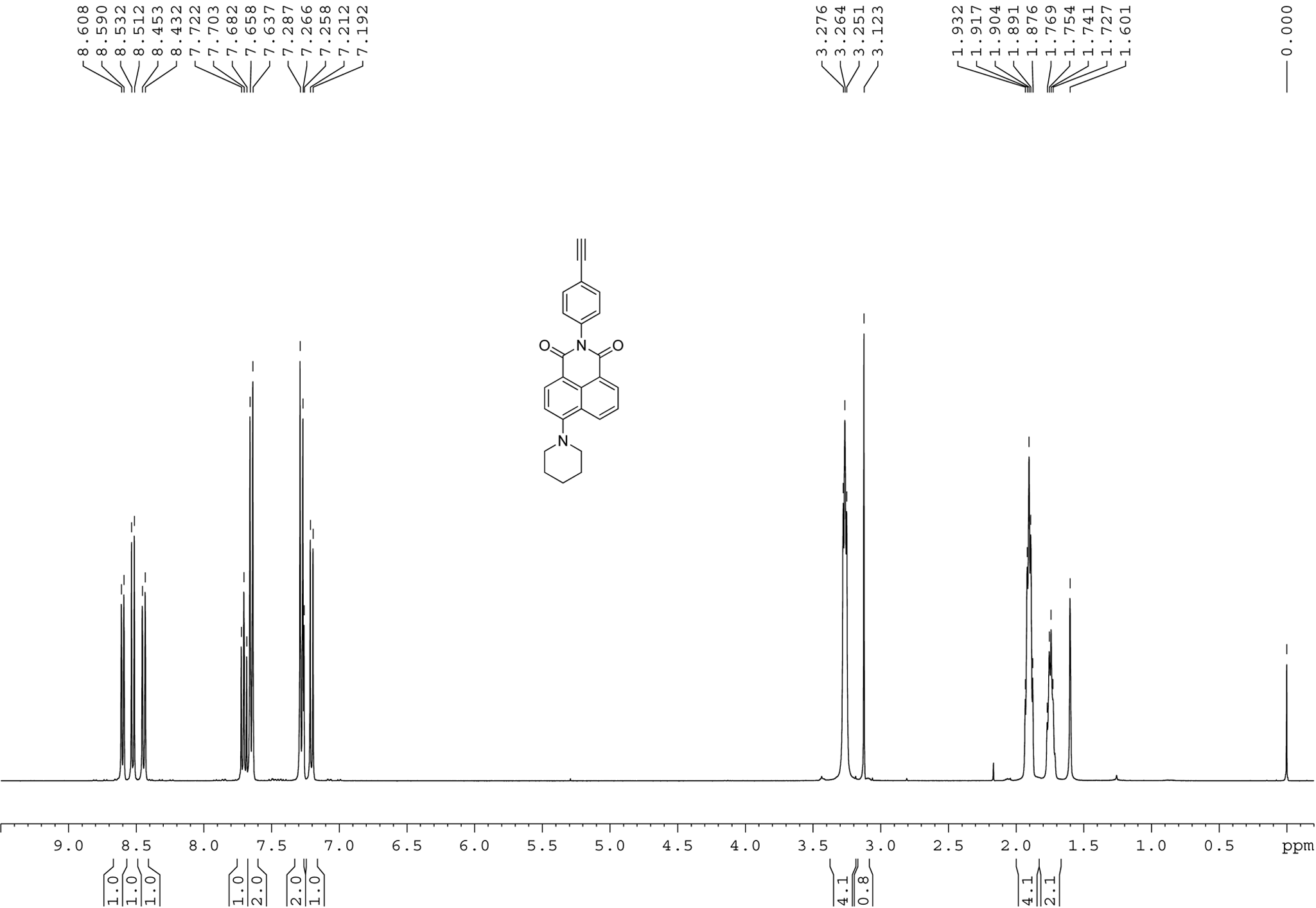

Supplement: File S1 — Contains the files: Text S1. Lippert-Mataga Equation. Text S2. Synthesis of Known Compounds. Figure S1. Stokes shift () of 8 versus orientation polarizability (Δ f ). The red, straight line represents the best linear fit to the 13 data points [coefficient of determination R 2 = 0.560, slope = (4.32±1.07)×103 cm−1, intercept = (4.41±0.26)×103 cm−1]. Figure S2. Stokes shift () of 9 versus orientation polarizability (Δ f ). The red, straight line represents the best linear fit to the 13 data points [coefficient of determination R 2 = 0.392, slope = (3.00±1.02)×103 cm−1, intercept = (4.61±0.25)×103 cm−1]. Figure S3. Stokes shift () of 10 versus orientation polarizability (Δ f ). The red, straight line represents the best linear fit to the 13 data points [coefficient of determination R 2 = 0.562, slope = (4.07±1.00)×103 cm−1, intercept = (4.53±0.25)×103 cm−1]. Figure S4. Fluorescence spectra of 8 (10 µM) in the presence of various metal ions. Experiments were carried out in HEPES buffer (10 mM, pH 7.4) at 25°C and the fluorescence emission spectra were recorded about 5 min after addition of various metal ions (1 equiv.). Figure S5. Fluorescence spectra of 9 (10 µM) in the presence of various metal ions. Experiments were carried out in HEPES buffer (10 mM, pH 7.4) at 25°C and the fluorescence emission spectra were recorded about 5 min after addition of various metal ions (1 equiv.). Figure S6. Fluorescence spectra of 10 (10 µM) in the presence of various metal ions. Experiments were carried out in HEPES buffer (10 mM, pH 7.4) at 25°C and the fluorescence emission spectra were recorded about 5 min after addition of various metal ions (1 equiv.). Figure S7. UV-Vis spectra of 8 (10 µM) in the presence of various metal ions. Experiments were carried out in HEPES buffer (10 mM, pH 7.4) at 25°C and the UV-Vis spectra were recorded about 5 min after addition of various metal ions (1 equiv.). Figure S8. UV-Vis spectra of 9 (10 µM) in the presence of various metal ions. Ex [file pone.0100761.s001.zip › SI/Figure S28.tif]

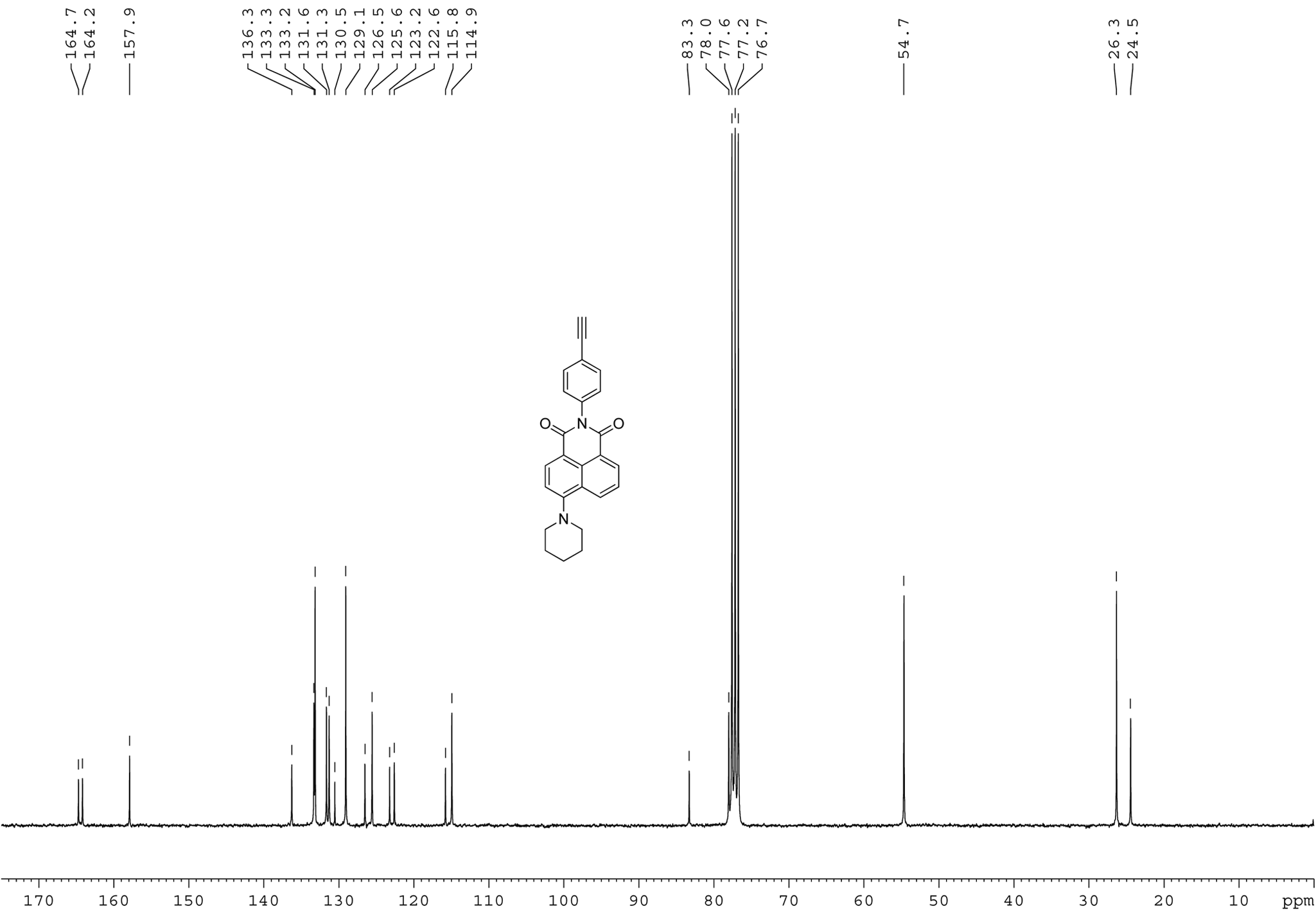

Supplement: File S1 — Contains the files: Text S1. Lippert-Mataga Equation. Text S2. Synthesis of Known Compounds. Figure S1. Stokes shift () of 8 versus orientation polarizability (Δ f ). The red, straight line represents the best linear fit to the 13 data points [coefficient of determination R 2 = 0.560, slope = (4.32±1.07)×103 cm−1, intercept = (4.41±0.26)×103 cm−1]. Figure S2. Stokes shift () of 9 versus orientation polarizability (Δ f ). The red, straight line represents the best linear fit to the 13 data points [coefficient of determination R 2 = 0.392, slope = (3.00±1.02)×103 cm−1, intercept = (4.61±0.25)×103 cm−1]. Figure S3. Stokes shift () of 10 versus orientation polarizability (Δ f ). The red, straight line represents the best linear fit to the 13 data points [coefficient of determination R 2 = 0.562, slope = (4.07±1.00)×103 cm−1, intercept = (4.53±0.25)×103 cm−1]. Figure S4. Fluorescence spectra of 8 (10 µM) in the presence of various metal ions. Experiments were carried out in HEPES buffer (10 mM, pH 7.4) at 25°C and the fluorescence emission spectra were recorded about 5 min after addition of various metal ions (1 equiv.). Figure S5. Fluorescence spectra of 9 (10 µM) in the presence of various metal ions. Experiments were carried out in HEPES buffer (10 mM, pH 7.4) at 25°C and the fluorescence emission spectra were recorded about 5 min after addition of various metal ions (1 equiv.). Figure S6. Fluorescence spectra of 10 (10 µM) in the presence of various metal ions. Experiments were carried out in HEPES buffer (10 mM, pH 7.4) at 25°C and the fluorescence emission spectra were recorded about 5 min after addition of various metal ions (1 equiv.). Figure S7. UV-Vis spectra of 8 (10 µM) in the presence of various metal ions. Experiments were carried out in HEPES buffer (10 mM, pH 7.4) at 25°C and the UV-Vis spectra were recorded about 5 min after addition of various metal ions (1 equiv.). Figure S8. UV-Vis spectra of 9 (10 µM) in the presence of various metal ions. Ex [file pone.0100761.s001.zip › SI/Figure S29.tif]

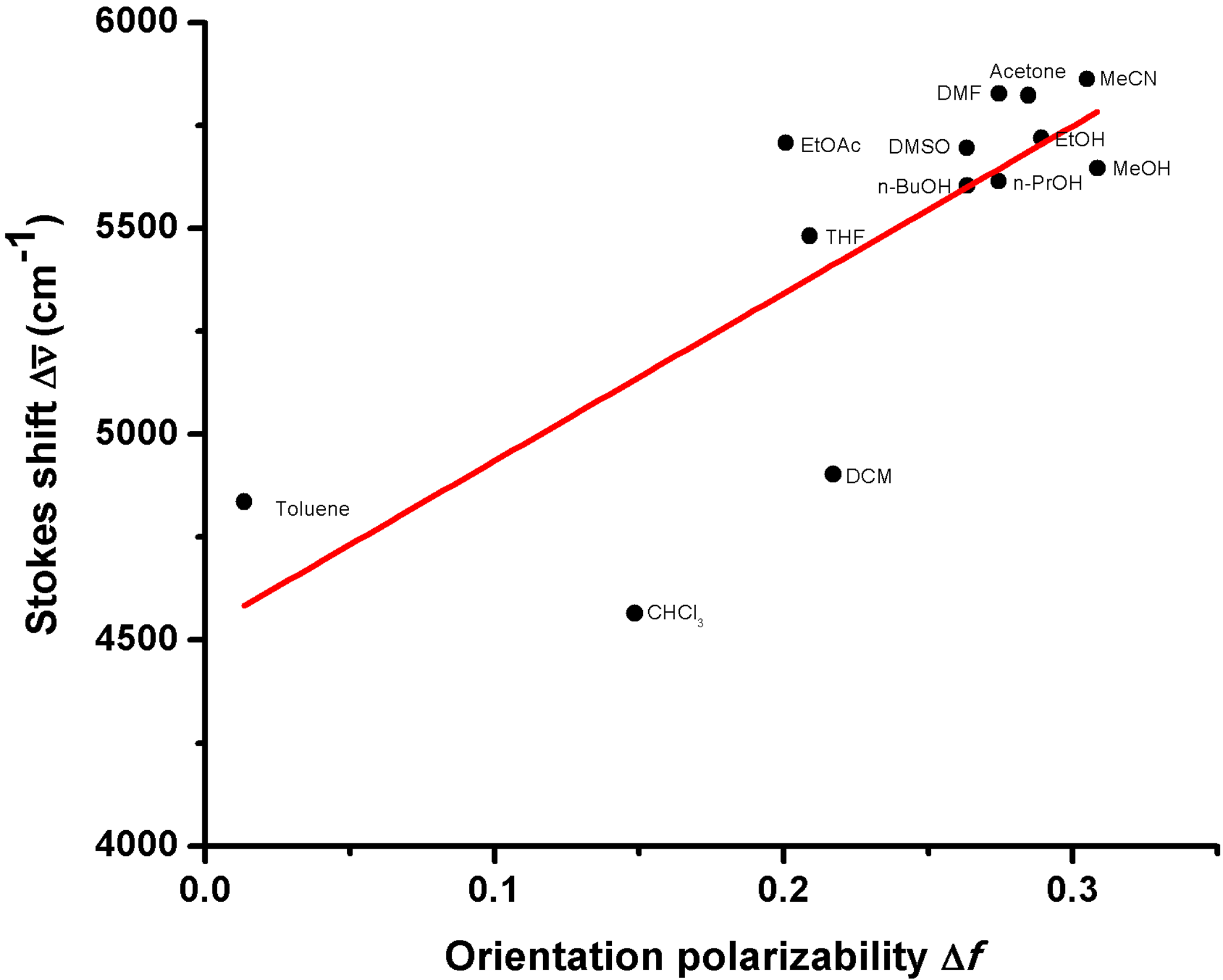

Supplement: File S1 — Contains the files: Text S1. Lippert-Mataga Equation. Text S2. Synthesis of Known Compounds. Figure S1. Stokes shift () of 8 versus orientation polarizability (Δ f ). The red, straight line represents the best linear fit to the 13 data points [coefficient of determination R 2 = 0.560, slope = (4.32±1.07)×103 cm−1, intercept = (4.41±0.26)×103 cm−1]. Figure S2. Stokes shift () of 9 versus orientation polarizability (Δ f ). The red, straight line represents the best linear fit to the 13 data points [coefficient of determination R 2 = 0.392, slope = (3.00±1.02)×103 cm−1, intercept = (4.61±0.25)×103 cm−1]. Figure S3. Stokes shift () of 10 versus orientation polarizability (Δ f ). The red, straight line represents the best linear fit to the 13 data points [coefficient of determination R 2 = 0.562, slope = (4.07±1.00)×103 cm−1, intercept = (4.53±0.25)×103 cm−1]. Figure S4. Fluorescence spectra of 8 (10 µM) in the presence of various metal ions. Experiments were carried out in HEPES buffer (10 mM, pH 7.4) at 25°C and the fluorescence emission spectra were recorded about 5 min after addition of various metal ions (1 equiv.). Figure S5. Fluorescence spectra of 9 (10 µM) in the presence of various metal ions. Experiments were carried out in HEPES buffer (10 mM, pH 7.4) at 25°C and the fluorescence emission spectra were recorded about 5 min after addition of various metal ions (1 equiv.). Figure S6. Fluorescence spectra of 10 (10 µM) in the presence of various metal ions. Experiments were carried out in HEPES buffer (10 mM, pH 7.4) at 25°C and the fluorescence emission spectra were recorded about 5 min after addition of various metal ions (1 equiv.). Figure S7. UV-Vis spectra of 8 (10 µM) in the presence of various metal ions. Experiments were carried out in HEPES buffer (10 mM, pH 7.4) at 25°C and the UV-Vis spectra were recorded about 5 min after addition of various metal ions (1 equiv.). Figure S8. UV-Vis spectra of 9 (10 µM) in the presence of various metal ions. Ex [file pone.0100761.s001.zip › SI/Figure S3.tif]

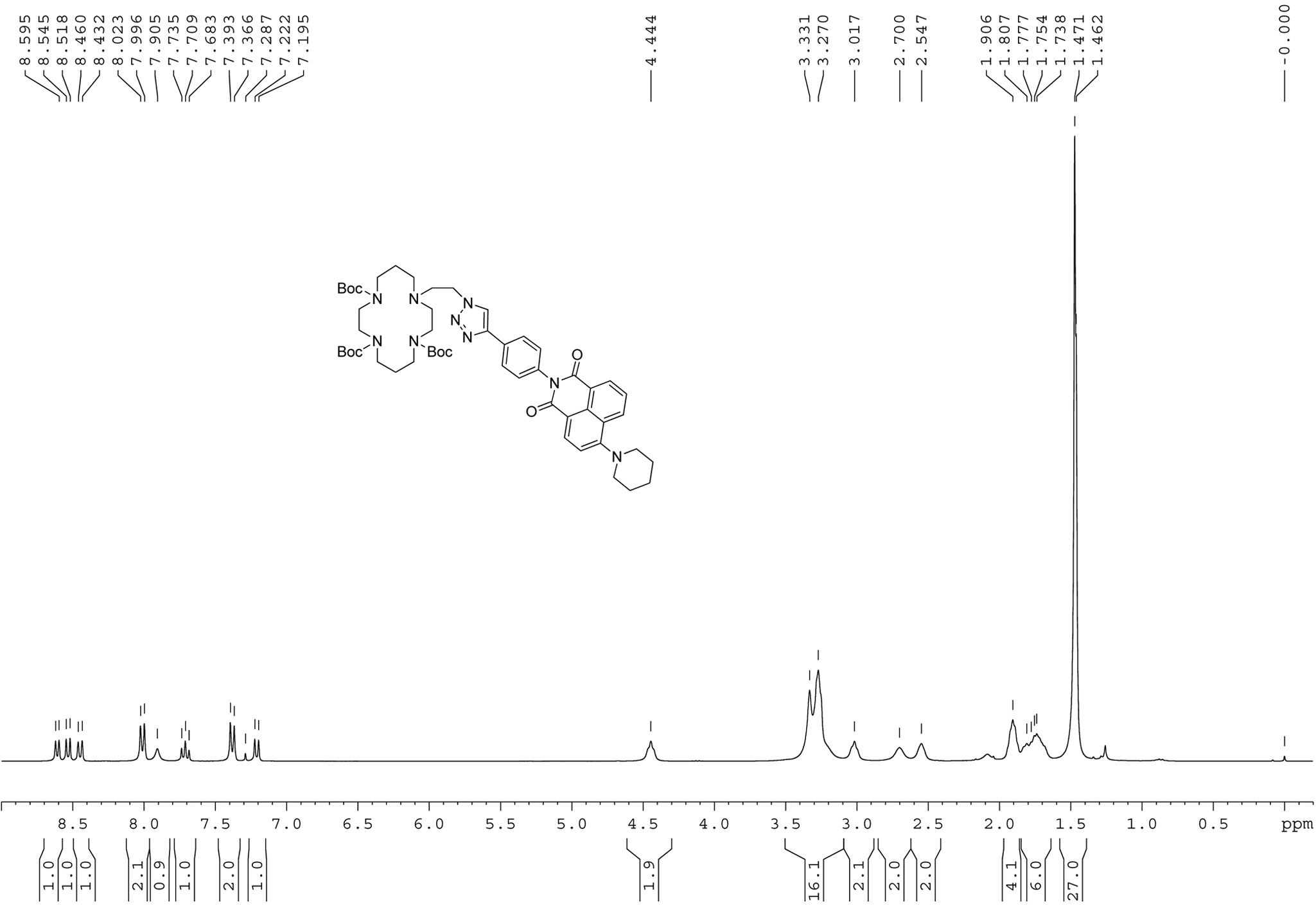

Supplement: File S1 — Contains the files: Text S1. Lippert-Mataga Equation. Text S2. Synthesis of Known Compounds. Figure S1. Stokes shift () of 8 versus orientation polarizability (Δ f ). The red, straight line represents the best linear fit to the 13 data points [coefficient of determination R 2 = 0.560, slope = (4.32±1.07)×103 cm−1, intercept = (4.41±0.26)×103 cm−1]. Figure S2. Stokes shift () of 9 versus orientation polarizability (Δ f ). The red, straight line represents the best linear fit to the 13 data points [coefficient of determination R 2 = 0.392, slope = (3.00±1.02)×103 cm−1, intercept = (4.61±0.25)×103 cm−1]. Figure S3. Stokes shift () of 10 versus orientation polarizability (Δ f ). The red, straight line represents the best linear fit to the 13 data points [coefficient of determination R 2 = 0.562, slope = (4.07±1.00)×103 cm−1, intercept = (4.53±0.25)×103 cm−1]. Figure S4. Fluorescence spectra of 8 (10 µM) in the presence of various metal ions. Experiments were carried out in HEPES buffer (10 mM, pH 7.4) at 25°C and the fluorescence emission spectra were recorded about 5 min after addition of various metal ions (1 equiv.). Figure S5. Fluorescence spectra of 9 (10 µM) in the presence of various metal ions. Experiments were carried out in HEPES buffer (10 mM, pH 7.4) at 25°C and the fluorescence emission spectra were recorded about 5 min after addition of various metal ions (1 equiv.). Figure S6. Fluorescence spectra of 10 (10 µM) in the presence of various metal ions. Experiments were carried out in HEPES buffer (10 mM, pH 7.4) at 25°C and the fluorescence emission spectra were recorded about 5 min after addition of various metal ions (1 equiv.). Figure S7. UV-Vis spectra of 8 (10 µM) in the presence of various metal ions. Experiments were carried out in HEPES buffer (10 mM, pH 7.4) at 25°C and the UV-Vis spectra were recorded about 5 min after addition of various metal ions (1 equiv.). Figure S8. UV-Vis spectra of 9 (10 µM) in the presence of various metal ions. Ex [file pone.0100761.s001.zip › SI/Figure S30.tif]

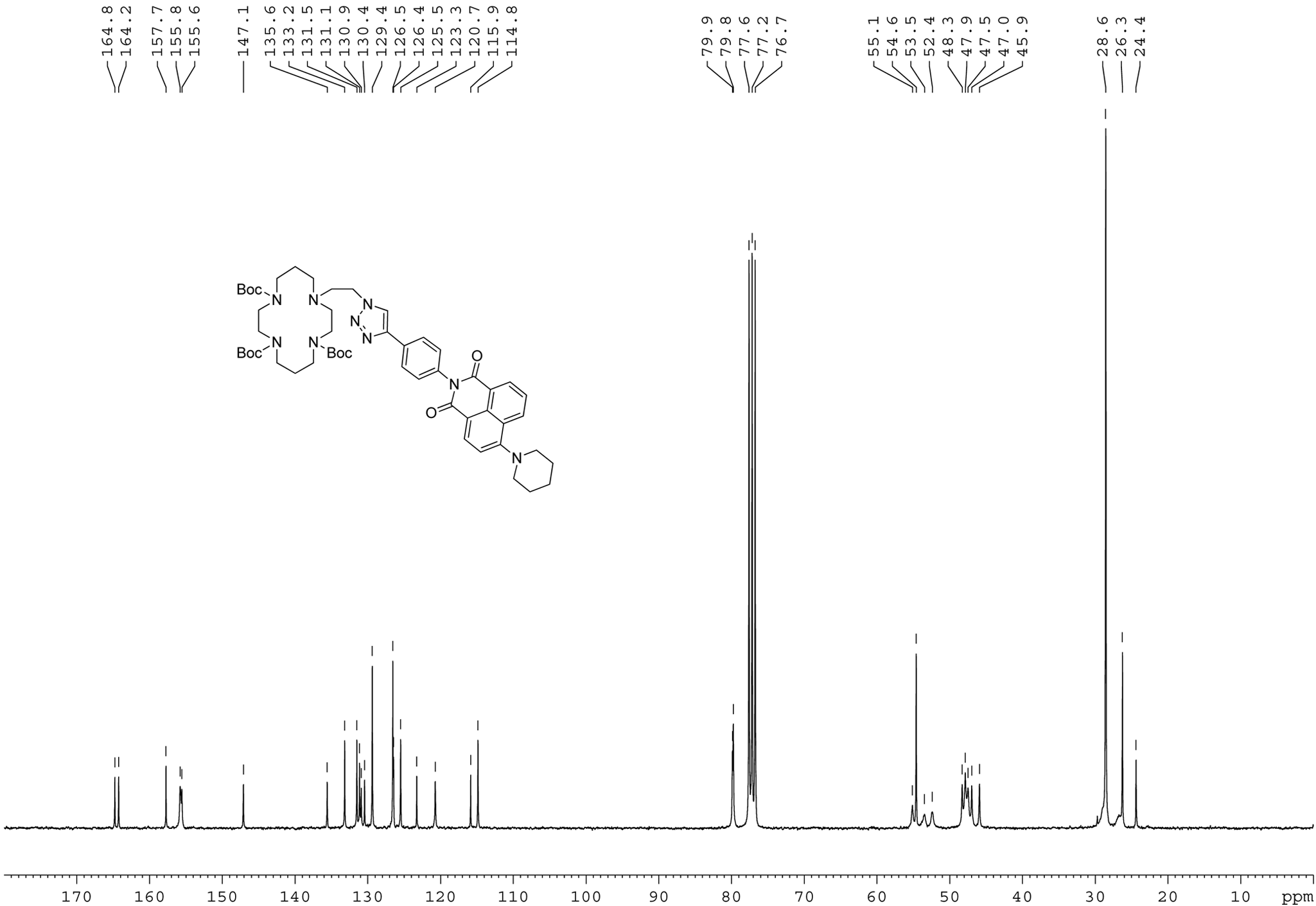

Supplement: File S1 — Contains the files: Text S1. Lippert-Mataga Equation. Text S2. Synthesis of Known Compounds. Figure S1. Stokes shift () of 8 versus orientation polarizability (Δ f ). The red, straight line represents the best linear fit to the 13 data points [coefficient of determination R 2 = 0.560, slope = (4.32±1.07)×103 cm−1, intercept = (4.41±0.26)×103 cm−1]. Figure S2. Stokes shift () of 9 versus orientation polarizability (Δ f ). The red, straight line represents the best linear fit to the 13 data points [coefficient of determination R 2 = 0.392, slope = (3.00±1.02)×103 cm−1, intercept = (4.61±0.25)×103 cm−1]. Figure S3. Stokes shift () of 10 versus orientation polarizability (Δ f ). The red, straight line represents the best linear fit to the 13 data points [coefficient of determination R 2 = 0.562, slope = (4.07±1.00)×103 cm−1, intercept = (4.53±0.25)×103 cm−1]. Figure S4. Fluorescence spectra of 8 (10 µM) in the presence of various metal ions. Experiments were carried out in HEPES buffer (10 mM, pH 7.4) at 25°C and the fluorescence emission spectra were recorded about 5 min after addition of various metal ions (1 equiv.). Figure S5. Fluorescence spectra of 9 (10 µM) in the presence of various metal ions. Experiments were carried out in HEPES buffer (10 mM, pH 7.4) at 25°C and the fluorescence emission spectra were recorded about 5 min after addition of various metal ions (1 equiv.). Figure S6. Fluorescence spectra of 10 (10 µM) in the presence of various metal ions. Experiments were carried out in HEPES buffer (10 mM, pH 7.4) at 25°C and the fluorescence emission spectra were recorded about 5 min after addition of various metal ions (1 equiv.). Figure S7. UV-Vis spectra of 8 (10 µM) in the presence of various metal ions. Experiments were carried out in HEPES buffer (10 mM, pH 7.4) at 25°C and the UV-Vis spectra were recorded about 5 min after addition of various metal ions (1 equiv.). Figure S8. UV-Vis spectra of 9 (10 µM) in the presence of various metal ions. Ex [file pone.0100761.s001.zip › SI/Figure S31.tif]

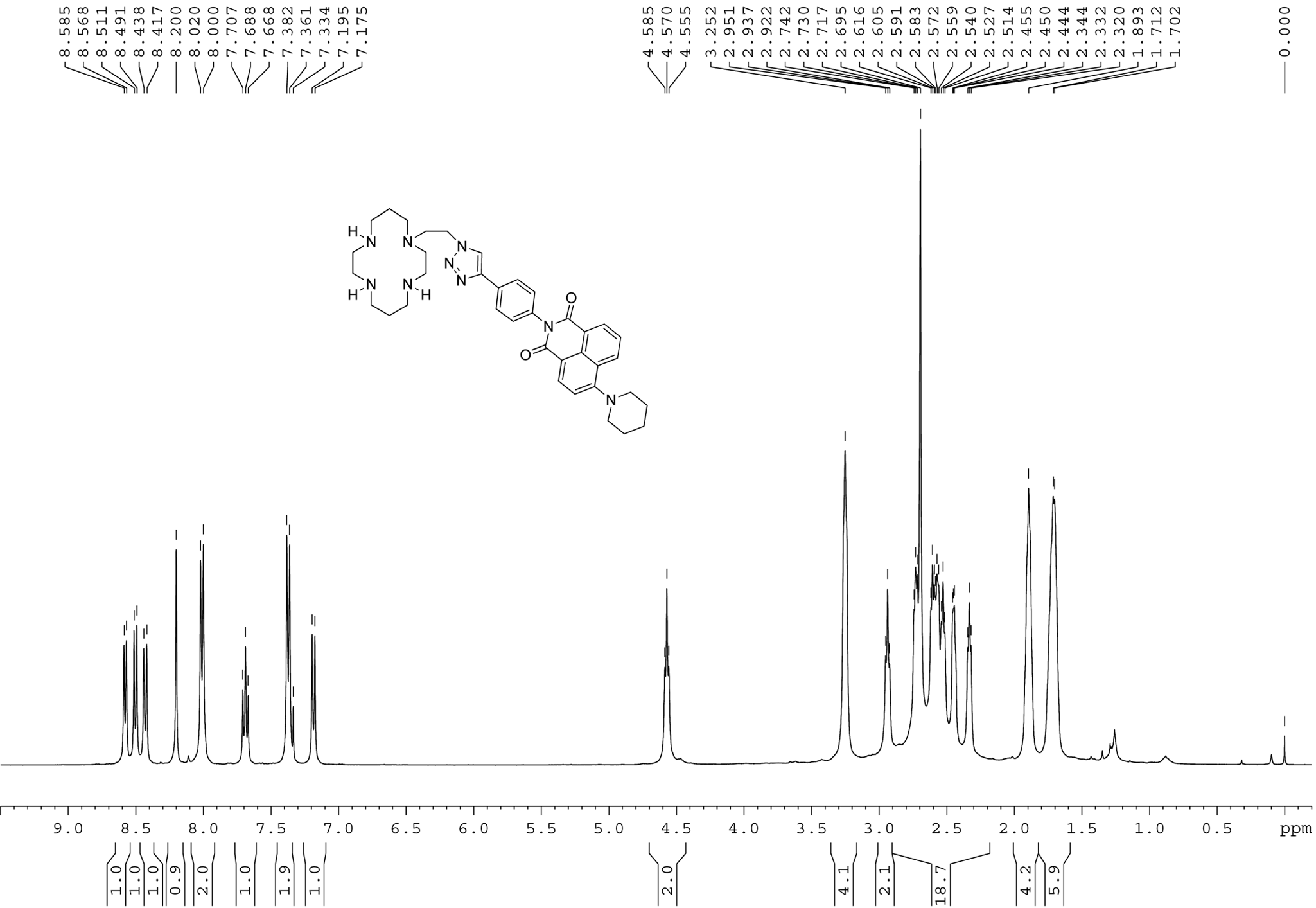

Supplement: File S1 — Contains the files: Text S1. Lippert-Mataga Equation. Text S2. Synthesis of Known Compounds. Figure S1. Stokes shift () of 8 versus orientation polarizability (Δ f ). The red, straight line represents the best linear fit to the 13 data points [coefficient of determination R 2 = 0.560, slope = (4.32±1.07)×103 cm−1, intercept = (4.41±0.26)×103 cm−1]. Figure S2. Stokes shift () of 9 versus orientation polarizability (Δ f ). The red, straight line represents the best linear fit to the 13 data points [coefficient of determination R 2 = 0.392, slope = (3.00±1.02)×103 cm−1, intercept = (4.61±0.25)×103 cm−1]. Figure S3. Stokes shift () of 10 versus orientation polarizability (Δ f ). The red, straight line represents the best linear fit to the 13 data points [coefficient of determination R 2 = 0.562, slope = (4.07±1.00)×103 cm−1, intercept = (4.53±0.25)×103 cm−1]. Figure S4. Fluorescence spectra of 8 (10 µM) in the presence of various metal ions. Experiments were carried out in HEPES buffer (10 mM, pH 7.4) at 25°C and the fluorescence emission spectra were recorded about 5 min after addition of various metal ions (1 equiv.). Figure S5. Fluorescence spectra of 9 (10 µM) in the presence of various metal ions. Experiments were carried out in HEPES buffer (10 mM, pH 7.4) at 25°C and the fluorescence emission spectra were recorded about 5 min after addition of various metal ions (1 equiv.). Figure S6. Fluorescence spectra of 10 (10 µM) in the presence of various metal ions. Experiments were carried out in HEPES buffer (10 mM, pH 7.4) at 25°C and the fluorescence emission spectra were recorded about 5 min after addition of various metal ions (1 equiv.). Figure S7. UV-Vis spectra of 8 (10 µM) in the presence of various metal ions. Experiments were carried out in HEPES buffer (10 mM, pH 7.4) at 25°C and the UV-Vis spectra were recorded about 5 min after addition of various metal ions (1 equiv.). Figure S8. UV-Vis spectra of 9 (10 µM) in the presence of various metal ions. Ex [file pone.0100761.s001.zip › SI/Figure S32.tif]

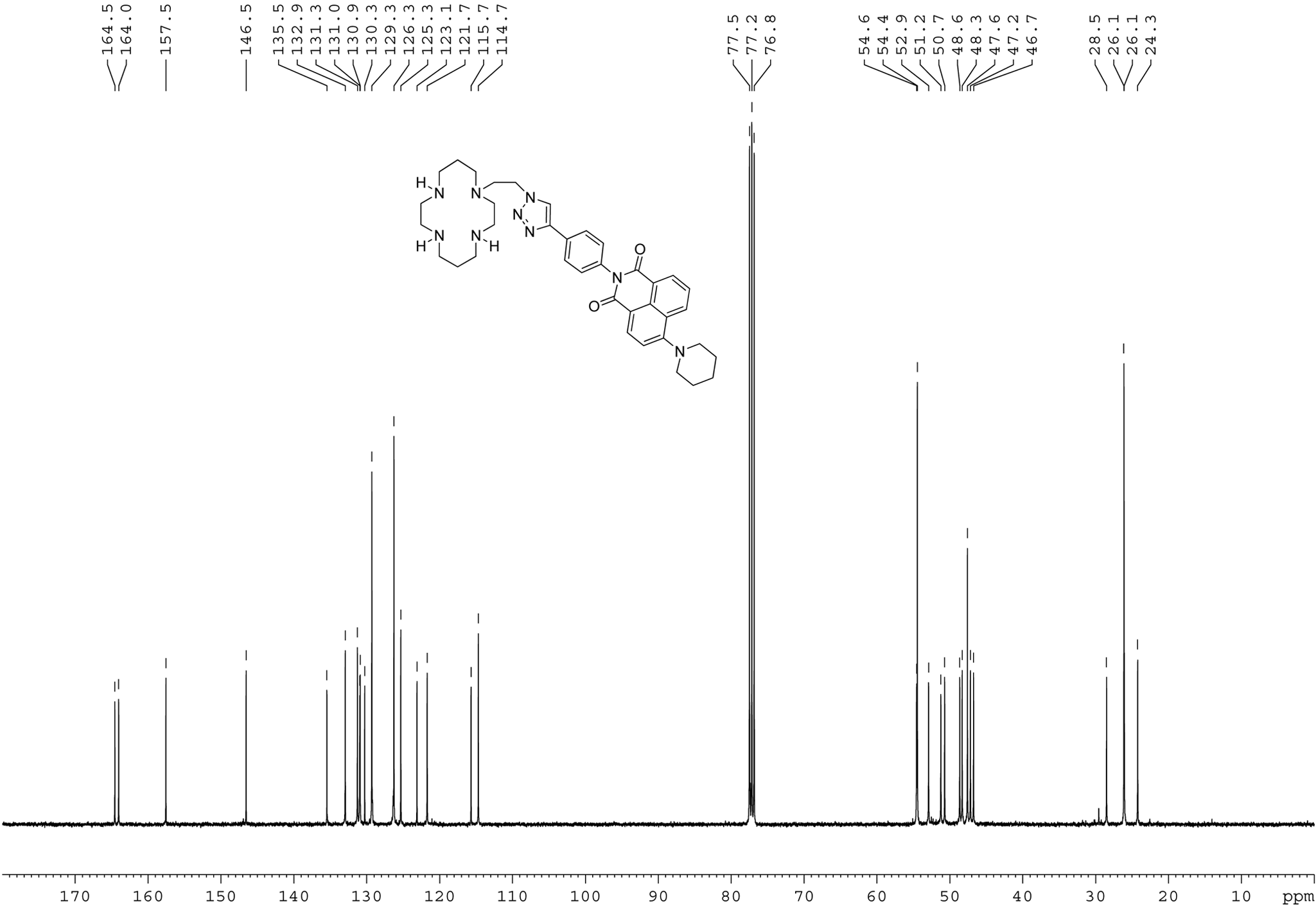

Supplement: File S1 — Contains the files: Text S1. Lippert-Mataga Equation. Text S2. Synthesis of Known Compounds. Figure S1. Stokes shift () of 8 versus orientation polarizability (Δ f ). The red, straight line represents the best linear fit to the 13 data points [coefficient of determination R 2 = 0.560, slope = (4.32±1.07)×103 cm−1, intercept = (4.41±0.26)×103 cm−1]. Figure S2. Stokes shift () of 9 versus orientation polarizability (Δ f ). The red, straight line represents the best linear fit to the 13 data points [coefficient of determination R 2 = 0.392, slope = (3.00±1.02)×103 cm−1, intercept = (4.61±0.25)×103 cm−1]. Figure S3. Stokes shift () of 10 versus orientation polarizability (Δ f ). The red, straight line represents the best linear fit to the 13 data points [coefficient of determination R 2 = 0.562, slope = (4.07±1.00)×103 cm−1, intercept = (4.53±0.25)×103 cm−1]. Figure S4. Fluorescence spectra of 8 (10 µM) in the presence of various metal ions. Experiments were carried out in HEPES buffer (10 mM, pH 7.4) at 25°C and the fluorescence emission spectra were recorded about 5 min after addition of various metal ions (1 equiv.). Figure S5. Fluorescence spectra of 9 (10 µM) in the presence of various metal ions. Experiments were carried out in HEPES buffer (10 mM, pH 7.4) at 25°C and the fluorescence emission spectra were recorded about 5 min after addition of various metal ions (1 equiv.). Figure S6. Fluorescence spectra of 10 (10 µM) in the presence of various metal ions. Experiments were carried out in HEPES buffer (10 mM, pH 7.4) at 25°C and the fluorescence emission spectra were recorded about 5 min after addition of various metal ions (1 equiv.). Figure S7. UV-Vis spectra of 8 (10 µM) in the presence of various metal ions. Experiments were carried out in HEPES buffer (10 mM, pH 7.4) at 25°C and the UV-Vis spectra were recorded about 5 min after addition of various metal ions (1 equiv.). Figure S8. UV-Vis spectra of 9 (10 µM) in the presence of various metal ions. Ex [file pone.0100761.s001.zip › SI/Figure S33.tif]

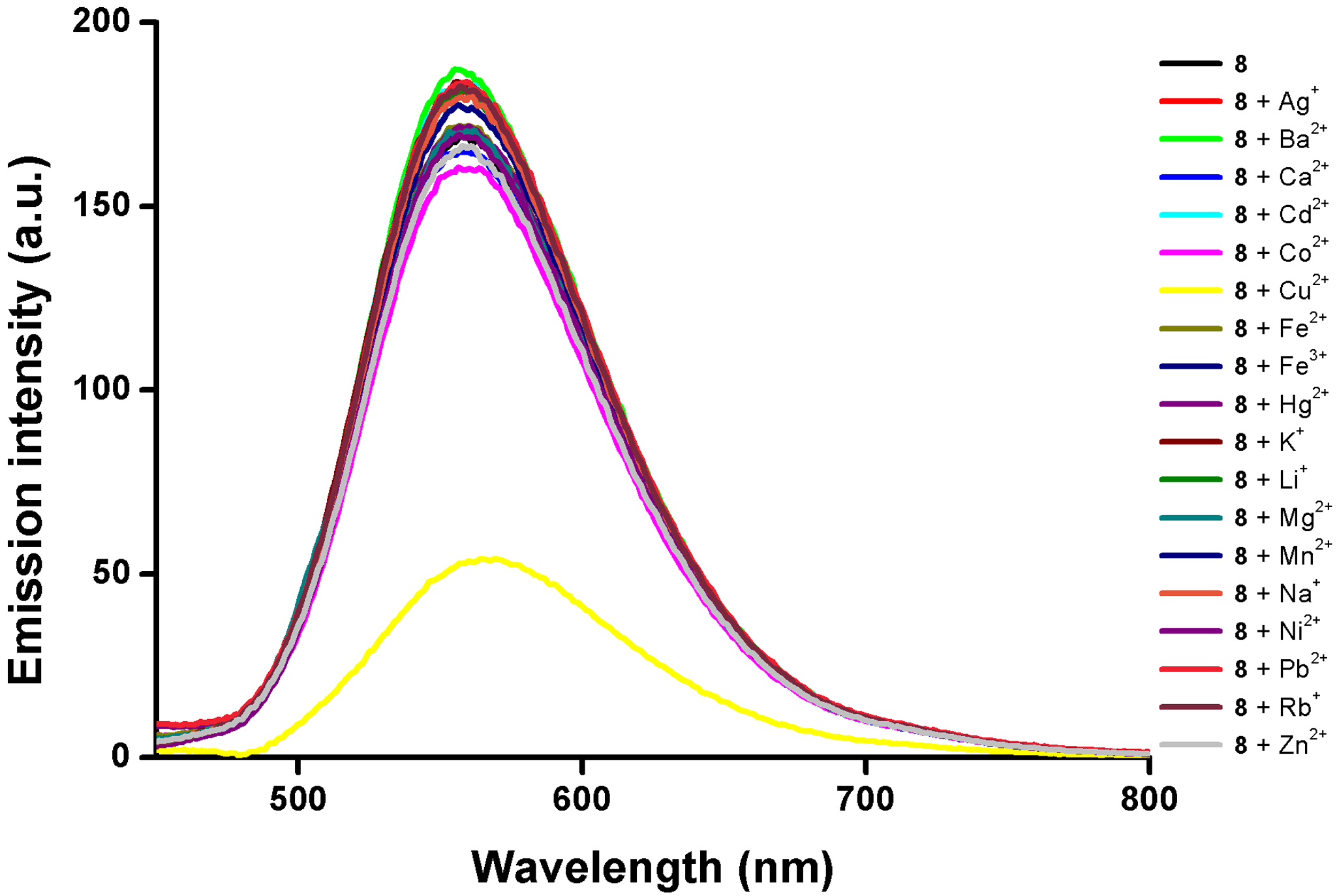

Supplement: File S1 — Contains the files: Text S1. Lippert-Mataga Equation. Text S2. Synthesis of Known Compounds. Figure S1. Stokes shift () of 8 versus orientation polarizability (Δ f ). The red, straight line represents the best linear fit to the 13 data points [coefficient of determination R 2 = 0.560, slope = (4.32±1.07)×103 cm−1, intercept = (4.41±0.26)×103 cm−1]. Figure S2. Stokes shift () of 9 versus orientation polarizability (Δ f ). The red, straight line represents the best linear fit to the 13 data points [coefficient of determination R 2 = 0.392, slope = (3.00±1.02)×103 cm−1, intercept = (4.61±0.25)×103 cm−1]. Figure S3. Stokes shift () of 10 versus orientation polarizability (Δ f ). The red, straight line represents the best linear fit to the 13 data points [coefficient of determination R 2 = 0.562, slope = (4.07±1.00)×103 cm−1, intercept = (4.53±0.25)×103 cm−1]. Figure S4. Fluorescence spectra of 8 (10 µM) in the presence of various metal ions. Experiments were carried out in HEPES buffer (10 mM, pH 7.4) at 25°C and the fluorescence emission spectra were recorded about 5 min after addition of various metal ions (1 equiv.). Figure S5. Fluorescence spectra of 9 (10 µM) in the presence of various metal ions. Experiments were carried out in HEPES buffer (10 mM, pH 7.4) at 25°C and the fluorescence emission spectra were recorded about 5 min after addition of various metal ions (1 equiv.). Figure S6. Fluorescence spectra of 10 (10 µM) in the presence of various metal ions. Experiments were carried out in HEPES buffer (10 mM, pH 7.4) at 25°C and the fluorescence emission spectra were recorded about 5 min after addition of various metal ions (1 equiv.). Figure S7. UV-Vis spectra of 8 (10 µM) in the presence of various metal ions. Experiments were carried out in HEPES buffer (10 mM, pH 7.4) at 25°C and the UV-Vis spectra were recorded about 5 min after addition of various metal ions (1 equiv.). Figure S8. UV-Vis spectra of 9 (10 µM) in the presence of various metal ions. Ex [file pone.0100761.s001.zip › SI/Figure S4.tif]

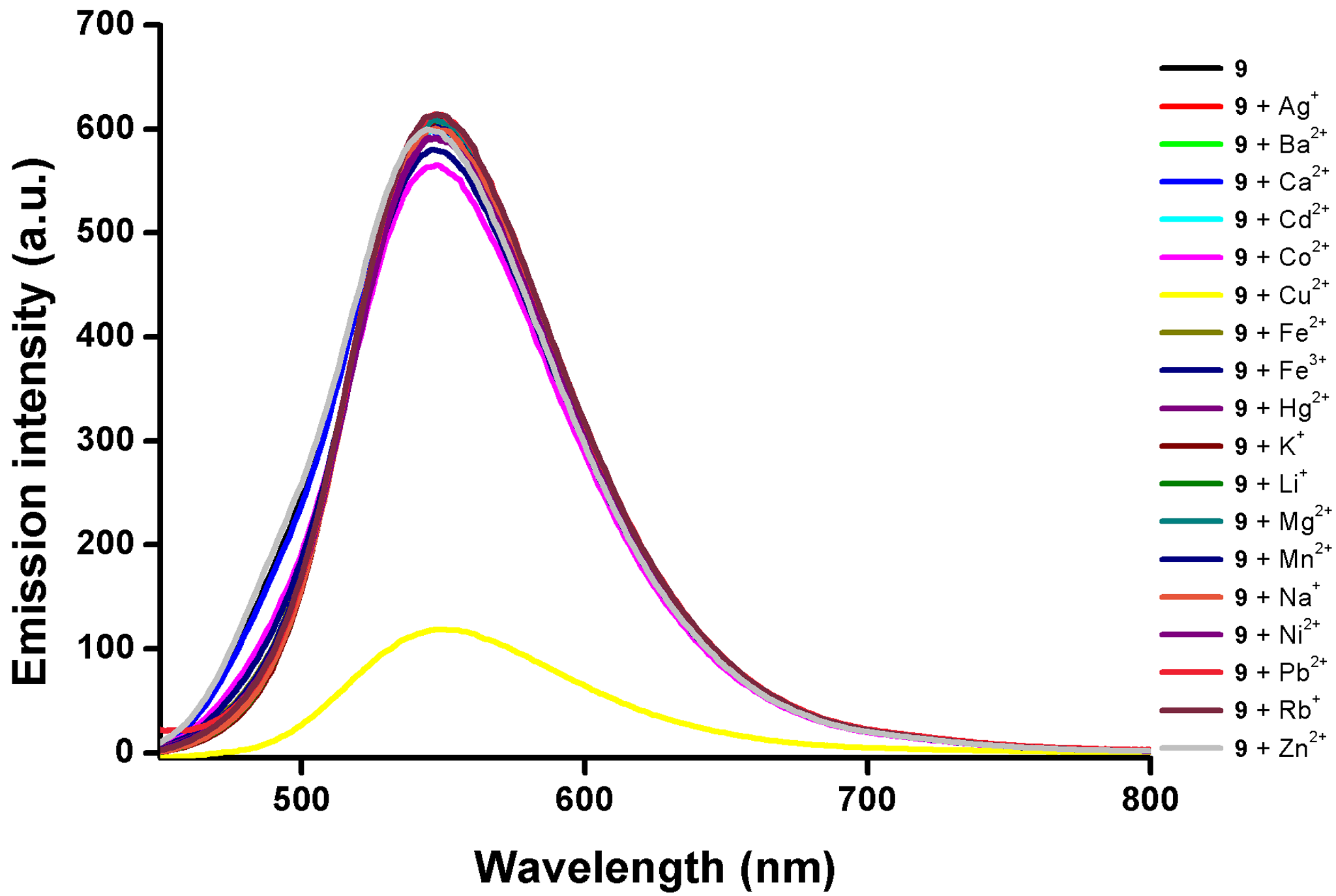

Supplement: File S1 — Contains the files: Text S1. Lippert-Mataga Equation. Text S2. Synthesis of Known Compounds. Figure S1. Stokes shift () of 8 versus orientation polarizability (Δ f ). The red, straight line represents the best linear fit to the 13 data points [coefficient of determination R 2 = 0.560, slope = (4.32±1.07)×103 cm−1, intercept = (4.41±0.26)×103 cm−1]. Figure S2. Stokes shift () of 9 versus orientation polarizability (Δ f ). The red, straight line represents the best linear fit to the 13 data points [coefficient of determination R 2 = 0.392, slope = (3.00±1.02)×103 cm−1, intercept = (4.61±0.25)×103 cm−1]. Figure S3. Stokes shift () of 10 versus orientation polarizability (Δ f ). The red, straight line represents the best linear fit to the 13 data points [coefficient of determination R 2 = 0.562, slope = (4.07±1.00)×103 cm−1, intercept = (4.53±0.25)×103 cm−1]. Figure S4. Fluorescence spectra of 8 (10 µM) in the presence of various metal ions. Experiments were carried out in HEPES buffer (10 mM, pH 7.4) at 25°C and the fluorescence emission spectra were recorded about 5 min after addition of various metal ions (1 equiv.). Figure S5. Fluorescence spectra of 9 (10 µM) in the presence of various metal ions. Experiments were carried out in HEPES buffer (10 mM, pH 7.4) at 25°C and the fluorescence emission spectra were recorded about 5 min after addition of various metal ions (1 equiv.). Figure S6. Fluorescence spectra of 10 (10 µM) in the presence of various metal ions. Experiments were carried out in HEPES buffer (10 mM, pH 7.4) at 25°C and the fluorescence emission spectra were recorded about 5 min after addition of various metal ions (1 equiv.). Figure S7. UV-Vis spectra of 8 (10 µM) in the presence of various metal ions. Experiments were carried out in HEPES buffer (10 mM, pH 7.4) at 25°C and the UV-Vis spectra were recorded about 5 min after addition of various metal ions (1 equiv.). Figure S8. UV-Vis spectra of 9 (10 µM) in the presence of various metal ions. Ex [file pone.0100761.s001.zip › SI/Figure S5.tif]

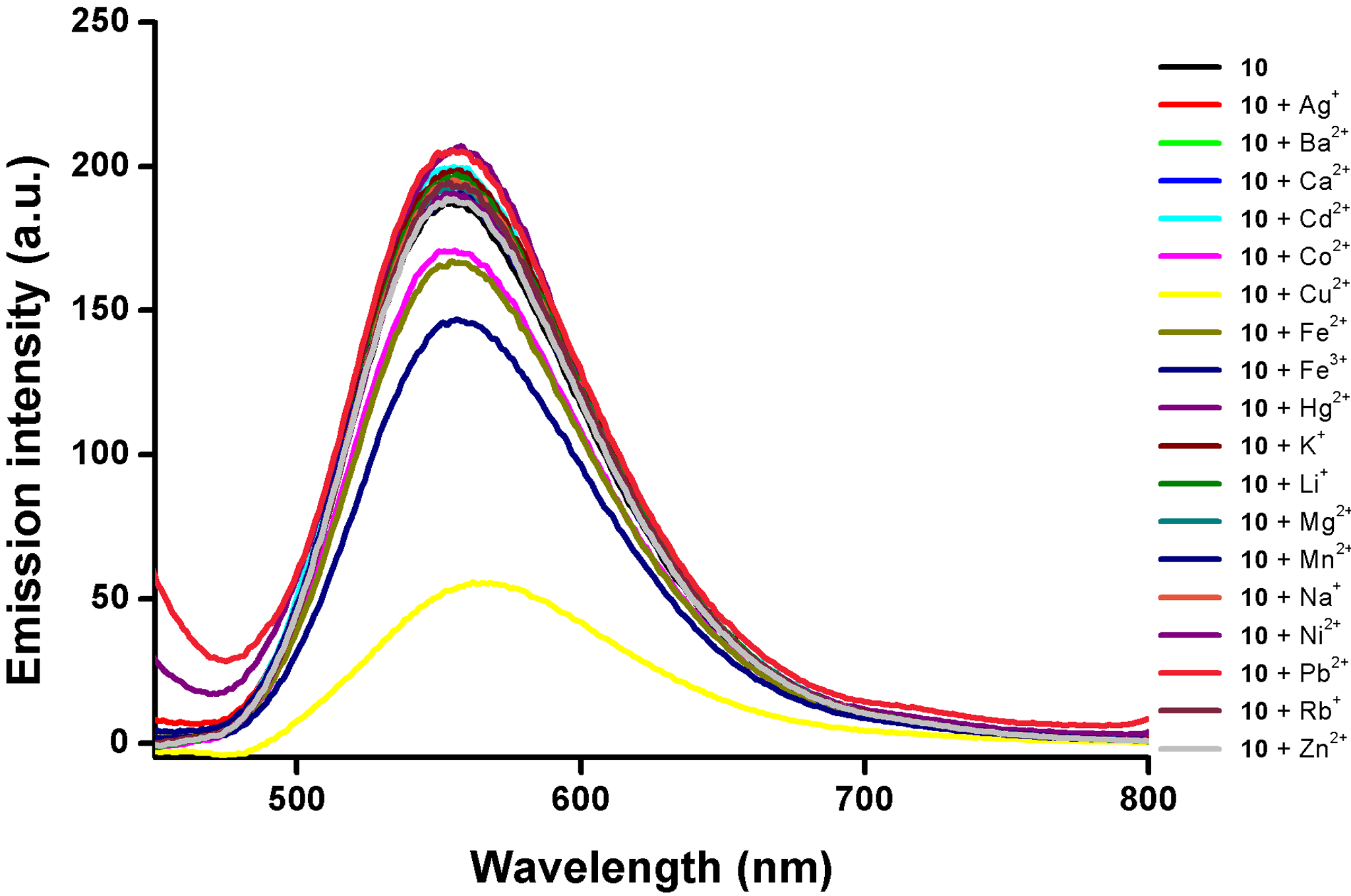

Supplement: File S1 — Contains the files: Text S1. Lippert-Mataga Equation. Text S2. Synthesis of Known Compounds. Figure S1. Stokes shift () of 8 versus orientation polarizability (Δ f ). The red, straight line represents the best linear fit to the 13 data points [coefficient of determination R 2 = 0.560, slope = (4.32±1.07)×103 cm−1, intercept = (4.41±0.26)×103 cm−1]. Figure S2. Stokes shift () of 9 versus orientation polarizability (Δ f ). The red, straight line represents the best linear fit to the 13 data points [coefficient of determination R 2 = 0.392, slope = (3.00±1.02)×103 cm−1, intercept = (4.61±0.25)×103 cm−1]. Figure S3. Stokes shift () of 10 versus orientation polarizability (Δ f ). The red, straight line represents the best linear fit to the 13 data points [coefficient of determination R 2 = 0.562, slope = (4.07±1.00)×103 cm−1, intercept = (4.53±0.25)×103 cm−1]. Figure S4. Fluorescence spectra of 8 (10 µM) in the presence of various metal ions. Experiments were carried out in HEPES buffer (10 mM, pH 7.4) at 25°C and the fluorescence emission spectra were recorded about 5 min after addition of various metal ions (1 equiv.). Figure S5. Fluorescence spectra of 9 (10 µM) in the presence of various metal ions. Experiments were carried out in HEPES buffer (10 mM, pH 7.4) at 25°C and the fluorescence emission spectra were recorded about 5 min after addition of various metal ions (1 equiv.). Figure S6. Fluorescence spectra of 10 (10 µM) in the presence of various metal ions. Experiments were carried out in HEPES buffer (10 mM, pH 7.4) at 25°C and the fluorescence emission spectra were recorded about 5 min after addition of various metal ions (1 equiv.). Figure S7. UV-Vis spectra of 8 (10 µM) in the presence of various metal ions. Experiments were carried out in HEPES buffer (10 mM, pH 7.4) at 25°C and the UV-Vis spectra were recorded about 5 min after addition of various metal ions (1 equiv.). Figure S8. UV-Vis spectra of 9 (10 µM) in the presence of various metal ions. Ex [file pone.0100761.s001.zip › SI/Figure S6.tif]

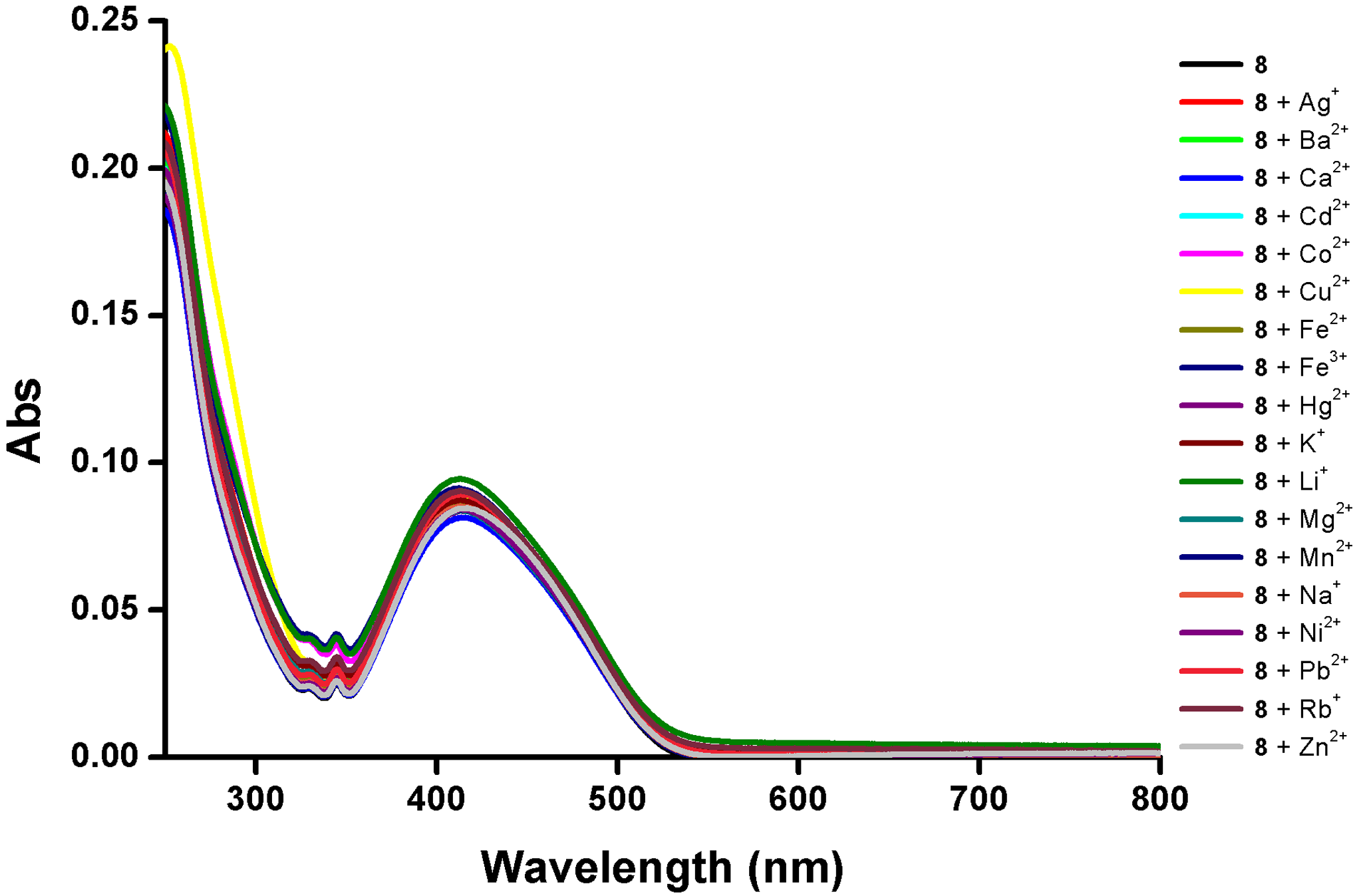

Supplement: File S1 — Contains the files: Text S1. Lippert-Mataga Equation. Text S2. Synthesis of Known Compounds. Figure S1. Stokes shift () of 8 versus orientation polarizability (Δ f ). The red, straight line represents the best linear fit to the 13 data points [coefficient of determination R 2 = 0.560, slope = (4.32±1.07)×103 cm−1, intercept = (4.41±0.26)×103 cm−1]. Figure S2. Stokes shift () of 9 versus orientation polarizability (Δ f ). The red, straight line represents the best linear fit to the 13 data points [coefficient of determination R 2 = 0.392, slope = (3.00±1.02)×103 cm−1, intercept = (4.61±0.25)×103 cm−1]. Figure S3. Stokes shift () of 10 versus orientation polarizability (Δ f ). The red, straight line represents the best linear fit to the 13 data points [coefficient of determination R 2 = 0.562, slope = (4.07±1.00)×103 cm−1, intercept = (4.53±0.25)×103 cm−1]. Figure S4. Fluorescence spectra of 8 (10 µM) in the presence of various metal ions. Experiments were carried out in HEPES buffer (10 mM, pH 7.4) at 25°C and the fluorescence emission spectra were recorded about 5 min after addition of various metal ions (1 equiv.). Figure S5. Fluorescence spectra of 9 (10 µM) in the presence of various metal ions. Experiments were carried out in HEPES buffer (10 mM, pH 7.4) at 25°C and the fluorescence emission spectra were recorded about 5 min after addition of various metal ions (1 equiv.). Figure S6. Fluorescence spectra of 10 (10 µM) in the presence of various metal ions. Experiments were carried out in HEPES buffer (10 mM, pH 7.4) at 25°C and the fluorescence emission spectra were recorded about 5 min after addition of various metal ions (1 equiv.). Figure S7. UV-Vis spectra of 8 (10 µM) in the presence of various metal ions. Experiments were carried out in HEPES buffer (10 mM, pH 7.4) at 25°C and the UV-Vis spectra were recorded about 5 min after addition of various metal ions (1 equiv.). Figure S8. UV-Vis spectra of 9 (10 µM) in the presence of various metal ions. Ex [file pone.0100761.s001.zip › SI/Figure S7.tif]

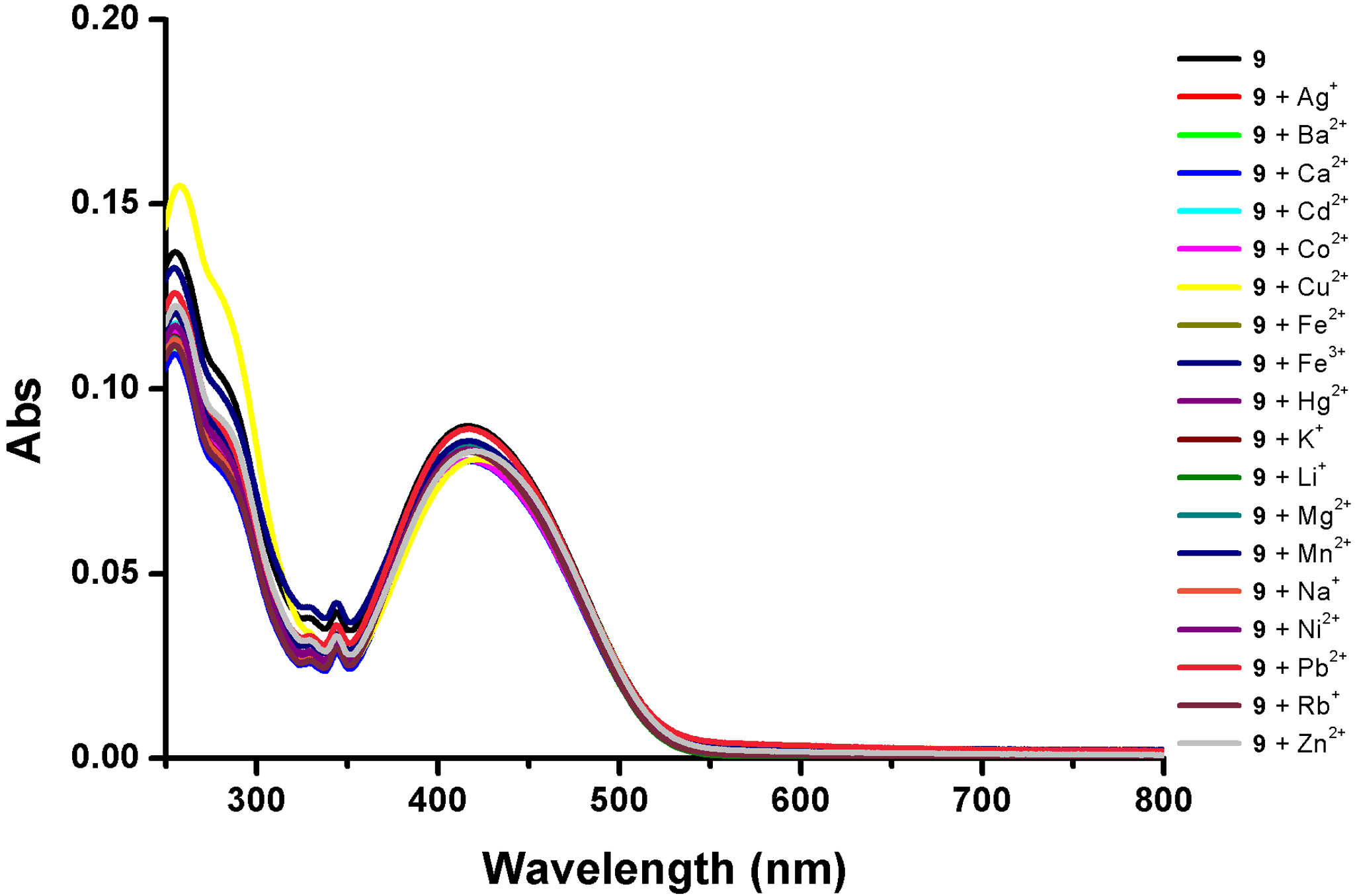

Supplement: File S1 — Contains the files: Text S1. Lippert-Mataga Equation. Text S2. Synthesis of Known Compounds. Figure S1. Stokes shift () of 8 versus orientation polarizability (Δ f ). The red, straight line represents the best linear fit to the 13 data points [coefficient of determination R 2 = 0.560, slope = (4.32±1.07)×103 cm−1, intercept = (4.41±0.26)×103 cm−1]. Figure S2. Stokes shift () of 9 versus orientation polarizability (Δ f ). The red, straight line represents the best linear fit to the 13 data points [coefficient of determination R 2 = 0.392, slope = (3.00±1.02)×103 cm−1, intercept = (4.61±0.25)×103 cm−1]. Figure S3. Stokes shift () of 10 versus orientation polarizability (Δ f ). The red, straight line represents the best linear fit to the 13 data points [coefficient of determination R 2 = 0.562, slope = (4.07±1.00)×103 cm−1, intercept = (4.53±0.25)×103 cm−1]. Figure S4. Fluorescence spectra of 8 (10 µM) in the presence of various metal ions. Experiments were carried out in HEPES buffer (10 mM, pH 7.4) at 25°C and the fluorescence emission spectra were recorded about 5 min after addition of various metal ions (1 equiv.). Figure S5. Fluorescence spectra of 9 (10 µM) in the presence of various metal ions. Experiments were carried out in HEPES buffer (10 mM, pH 7.4) at 25°C and the fluorescence emission spectra were recorded about 5 min after addition of various metal ions (1 equiv.). Figure S6. Fluorescence spectra of 10 (10 µM) in the presence of various metal ions. Experiments were carried out in HEPES buffer (10 mM, pH 7.4) at 25°C and the fluorescence emission spectra were recorded about 5 min after addition of various metal ions (1 equiv.). Figure S7. UV-Vis spectra of 8 (10 µM) in the presence of various metal ions. Experiments were carried out in HEPES buffer (10 mM, pH 7.4) at 25°C and the UV-Vis spectra were recorded about 5 min after addition of various metal ions (1 equiv.). Figure S8. UV-Vis spectra of 9 (10 µM) in the presence of various metal ions. Ex [file pone.0100761.s001.zip › SI/Figure S8.tif]

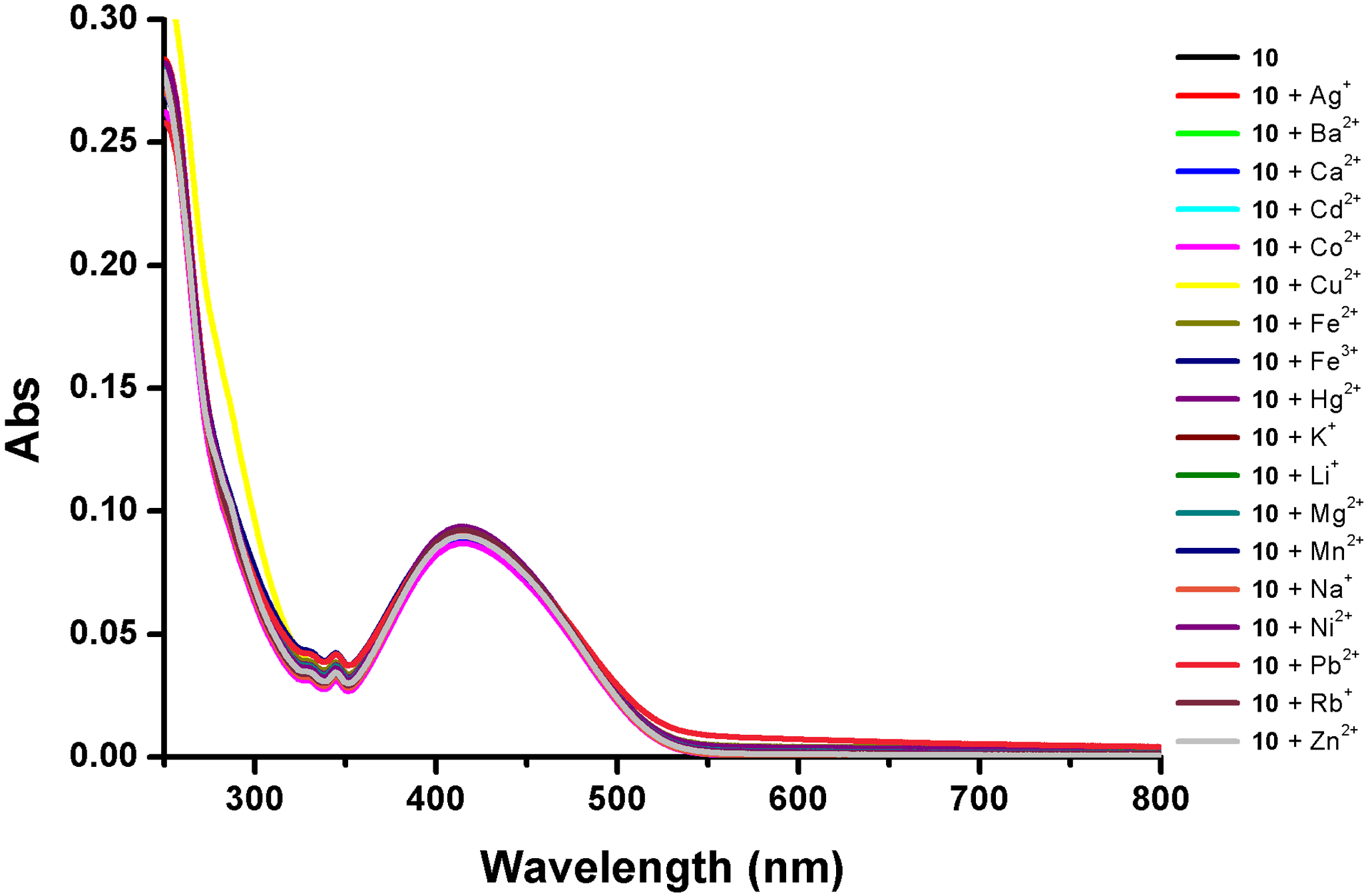

Supplement: File S1 — Contains the files: Text S1. Lippert-Mataga Equation. Text S2. Synthesis of Known Compounds. Figure S1. Stokes shift () of 8 versus orientation polarizability (Δ f ). The red, straight line represents the best linear fit to the 13 data points [coefficient of determination R 2 = 0.560, slope = (4.32±1.07)×103 cm−1, intercept = (4.41±0.26)×103 cm−1]. Figure S2. Stokes shift () of 9 versus orientation polarizability (Δ f ). The red, straight line represents the best linear fit to the 13 data points [coefficient of determination R 2 = 0.392, slope = (3.00±1.02)×103 cm−1, intercept = (4.61±0.25)×103 cm−1]. Figure S3. Stokes shift () of 10 versus orientation polarizability (Δ f ). The red, straight line represents the best linear fit to the 13 data points [coefficient of determination R 2 = 0.562, slope = (4.07±1.00)×103 cm−1, intercept = (4.53±0.25)×103 cm−1]. Figure S4. Fluorescence spectra of 8 (10 µM) in the presence of various metal ions. Experiments were carried out in HEPES buffer (10 mM, pH 7.4) at 25°C and the fluorescence emission spectra were recorded about 5 min after addition of various metal ions (1 equiv.). Figure S5. Fluorescence spectra of 9 (10 µM) in the presence of various metal ions. Experiments were carried out in HEPES buffer (10 mM, pH 7.4) at 25°C and the fluorescence emission spectra were recorded about 5 min after addition of various metal ions (1 equiv.). Figure S6. Fluorescence spectra of 10 (10 µM) in the presence of various metal ions. Experiments were carried out in HEPES buffer (10 mM, pH 7.4) at 25°C and the fluorescence emission spectra were recorded about 5 min after addition of various metal ions (1 equiv.). Figure S7. UV-Vis spectra of 8 (10 µM) in the presence of various metal ions. Experiments were carried out in HEPES buffer (10 mM, pH 7.4) at 25°C and the UV-Vis spectra were recorded about 5 min after addition of various metal ions (1 equiv.). Figure S8. UV-Vis spectra of 9 (10 µM) in the presence of various metal ions. Ex [file pone.0100761.s001.zip › SI/Figure S9.tif]
