# Supplementary material for: Incorporating a Piperidinyl Group in the Fluorophore Extends the Fluorescence Lifetime of Click-Derived Cyclam-Naphthalimide Conjugates
Source: PLoS One. 2014 Jul 1;9(7):e100761. doi: 10.1371/journal.pone.0100761 (PMC4077572; doi:10.1371/journal.pone.0100761)
Supplement: File S1 — Contains the files: Text S1. Lippert-Mataga Equation. Text S2. Synthesis of Known Compounds. Figure S1. Stokes shift () of 8 versus orientation polarizability (Δ f ). The red, straight line represents the best linear fit to the 13 data points [coefficient of determination R 2 = 0.560, slope = (4.32±1.07)×103 cm−1, intercept = (4.41±0.26)×103 cm−1]. Figure S2. Stokes shift () of 9 versus orientation polarizability (Δ f ). The red, straight line represents the best linear fit to the 13 data points [coefficient of determination R 2 = 0.392, slope = (3.00±1.02)×103 cm−1, intercept = (4.61±0.25)×103 cm−1]. Figure S3. Stokes shift () of 10 versus orientation polarizability (Δ f ). The red, straight line represents the best linear fit to the 13 data points [coefficient of determination R 2 = 0.562, slope = (4.07±1.00)×103 cm−1, intercept = (4.53±0.25)×103 cm−1]. Figure S4. Fluorescence spectra of 8 (10 µM) in the presence of various metal ions. Experiments were carried out in HEPES buffer (10 mM, pH 7.4) at 25°C and the fluorescence emission spectra were recorded about 5 min after addition of various metal ions (1 equiv.). Figure S5. Fluorescence spectra of 9 (10 µM) in the presence of various metal ions. Experiments were carried out in HEPES buffer (10 mM, pH 7.4) at 25°C and the fluorescence emission spectra were recorded about 5 min after addition of various metal ions (1 equiv.). Figure S6. Fluorescence spectra of 10 (10 µM) in the presence of various metal ions. Experiments were carried out in HEPES buffer (10 mM, pH 7.4) at 25°C and the fluorescence emission spectra were recorded about 5 min after addition of various metal ions (1 equiv.). Figure S7. UV-Vis spectra of 8 (10 µM) in the presence of various metal ions. Experiments were carried out in HEPES buffer (10 mM, pH 7.4) at 25°C and the UV-Vis spectra were recorded about 5 min after addition of various metal ions (1 equiv.). Figure S8. UV-Vis spectra of 9 (10 µM) in the presence of various metal ions. Ex [file pone.0100761.s001.zip › SI/Text S2_Synthesis of Known Compounds_20140505.doc]

**Synthesis of Known Compounds**

*Synthetic routes to known compounds have been shown in Figures S13-S15.*

**2-Azidoethyl 4-methylbenzenesulfonate (S2).**

To a solution of sodium azide (3.90 g, 60.0 mmol) in H_2_O (15 mL) was added 2-bromoethanol (6.25 g, 50.0 mmol). The reaction mixture was heated at reflux overnight, cooled to room temperature and extracted with DCM (2 × 20 mL). The combined organic extracts were dried over MgSO_4_ and filtered. To the filtrate were added *p*-toluenesulfonyl chloride (9.80 g, 51.4 mmol) and Et_3_N (10.0 mL, 71.8 mmol). The reaction mixture was stirred at room temperature for 6 h, and glycine (751 mg, 10.0 mmol) was added. After 2 h, the reaction mixture was washed with 1 M NaOH (2 × 50 mL) and saturated aqueous NH_4_Cl (2 × 50 mL), dried over MgSO_4_ and concentrated under reduced pressure. The residue was purified by flash column chromatography (silica gel, EtOAc:hexane = 5:95 ramping to 20:80) to give **S2** as a pale yellow oil (7.86 g, 65%). ***R*_F_** (EtOAc:hexane = 1:4) 0.33. **IR** *ν*_max_/cm^-1^ 2103, 1597, 1493, 1447, 1355, 1297, 1220, 1174, 1097, 1013, 909, 846, 816. **^1^H NMR** (300 MHz, CDCl_3_) *δ* 2.43 (s, 3H, CH_3_), 3.47 (t, 2H, *J* 5.1), 4.14 (t, 2H, *J* 5.1) (total 4H, N_3_CH_2_CH_2_), 7.36 (d, 2H, *J* 8.1, Ph-H), 7.79 (d, 2H, *J* 8.1, Ph-H). **^13^C NMR** (75 MHz, CDCl_3_) *δ* 21.3, 49.3, 68.2, 127.6, 129.8, 132.2, 145.1 (two carbon signals overlapping or obscured). **MS** (APCI) 213.9 ([M-N_2_+H]^+^, 100%). The spectroscopic data were in agreement with those in the literature.

**Tri-*tert*-butyl 11-(2-azidoethyl)-1,4,8,11-tetraazacyclotetradecane-1,4,8-tricarboxylate (S3).**

To a solution of tri-Boc cyclam (3.45 g, 6.89 mmol) in CH_3_CN (200 mL) were added azide **S2** (5.49 g, 22.8 mmol) and Na_2_CO_3_ (18.3 g, 17.3 mmol). The reaction mixture was heated at reflux for 96 h, cooled to room temperature and filtered. The filtrate was concentrated under reduced pressure, and the residue was purified by flash column chromatography (silica gel, EtOAc:hexane = 1:4 ramping to 1:3) to give **S3** as a pale yellow oil (2.59 g, 66%). ***R*_F_** (EtOAc:hexane = 1:1) 0.70. **IR** *ν*_max_/cm^-1^ 2974, 2932, 2869, 2818, 2097, 1683, 1463, 1410, 1391, 1365, 1296, 1245, 1153, 1112, 1060, 988, 946, 895, 862. **^1^H NMR** (300 MHz, CDCl_3_) *δ* 1.46 (s, 27H, 3 × C(CH_3_)_3_), 1.60-1.80 (m, 2H, CH_2_C*H*_2_CH_2_), 1.80-2.00 (m, 2H, CH_2_C*H*_2_CH_2_), 2.42-2.54 (m, 2H, C*H*_2_N(CH_2_CH_2_N_3_)CH_2_), 2.56-2.74 (m, 4H, CH_2_N(C*H*_2_CH_2_N_3_)C*H*_2_), 3.20-3.50 (m, 14H, 3 × C*H*_2_N(Boc)C*H*_2_ & CH_2_C*H*_2_N_3_). **^13^C NMR** (100 MHz, CDCl_3_) *δ* 26.6, 28.5, 45.7, 46.9, 47.3, 49.1, 52.2, 53.8, 54.4, 79.6, 155.5, 155.7 (fifteen carbon signals overlapping or obscured). **MS** (ESI) 570.1 ([M+H]^+^, 53%), 592.2 ([M+Na]^+^, 39%), 1161.1 ([2M+Na]^+^, 100%). The spectroscopic data were in agreement with those in the literature.

**6-(Piperidin-1-yl)benzo[*de*]isochromene-1,3-dione (11).**

To a solution of 4-bromo-1,8-naphthalic anhydride **S4** (2.77 g, 10.0 mmol) in 2-methoxyethanol (25 mL) was added piperidine (1.98 mL, 20.0 mmol). The reaction mixture was heated at reflux under Ar for 36 h, cooled to room temperature and concentrated under reduced pressure. The residue was purified by flash column chromatography (silica gel, DCM) to give **11** as an orange solid (2.41 g, 86%). ***R*_F_** (DCM) 0.25. **m.p.** 175-176 °C (lit. **m.p.** 175-176 °C). **IR** *ν*_max_/cm^-1^ 2929, 2849, 1754, 1721, 1575, 1516, 1442, 1377, 1306, 1229, 1115, 1079, 1008, 908, 858. **^1^H NMR** (300 MHz, CDCl_3_) *δ* 1.72-1.80 (m, 2H, CH_2_CH_2_C*H*_2_CH_2_CH_2_), 1.87-1.95 (m, 4H, CH_2_C*H*_2_CH_2_C*H*_2_CH_2_), 3.30 (t, 4H, *J* 5.4, CH_2_NCH_2_), 7.16 (d, 1H, *J* 8.1, naphthalene-H), 7.67 (dd, 1H, *J* 8.4 & 7.5, naphthalene-H), 8.40 (d, 2H, *J* 8.1, naphthalene-H), 8.49 (dd, 1H, *J* 7.5 & 1.2, naphthalene-H). **^13^C NMR** (75 MHz, CDCl_3_) *δ* 24.3, 26.2, 54.4, 110.6, 114.9, 119.2, 125.6, 126.1, 132.3, 132.4, 133.1, 135.0, 158.5, 160.6, 161.5 (two carbon signals overlapping or obscured). **MS** (APCI) *m/z* 282.1 ([M+H]^+^, 100%). **HRMS** (ESI) 282.11227 ([M+H]^+^); calcd. for C_17_H_16_NO_3_ ([M+H]^+^) 282.11247. The spectroscopic data were in agreement with those in the literature.

**2-(4-Bromophenyl)-6-(piperidin-1-yl)-1*H*-benzo[*de*]isoquinoline-1,3(2*H*)-dione (12).**

To a solution of **11** (1.13 g, 4.02 mmol) and 4-bromoaniline (1.03 g, 5.99 mmol) in 2-methoxyethanol (25 mL) was added piperidine (1.50 mL, 15.2 mmol). The reaction mixture was heated at reflux under Ar for 72 h and cooled to room temperature. The precipitate was filtered off and washed with H_2_O to give **12** as a yellow solid (1.57 g, 90%). ***R*_F_** (DCM) 0.57. **m.p.** 294-295 °C (no lit. **m.p.**). **IR** *ν*_max_/cm^-1^ 2927, 2852, 2787, 1704, 1657, 1586, 1514, 1483, 1450, 1373, 1231, 1147, 1071, 1010. **^1^H NMR** (300 MHz, CDCl_3_) *δ* 1.70-1.78 (m, 2H, CH_2_CH_2_C*H*_2_CH_2_CH_2_), 1.86-1.95 (m, 4H, CH_2_C*H*_2_CH_2_C*H*_2_CH_2_), 3.27 (t, 4H, *J* 5.4, CH_2_NCH_2_), 7.16-7.22 (m, 2H, Ph-H), 7.20 (d, 1H, *J* 8.1, naphthalene-H), 7.62-7.68 (m, 2H, Ph-H), 7.70 (dd, 1H, *J* 8.4 & 7.5, naphthalene-H), 8.44 (dd, 1H, *J* 8.4 & 1.2, naphthalene-H), 8.52 (d, 1H, *J* 8.4, naphthalene-H), 8.60 (dd, 1H, *J* 7.5 & 1.2, naphthalene-H). **^13^C NMR** (75 MHz, CDCl_3_) *δ* 24.5, 26.4, 54.7, 114.9, 115.7, 122.6, 123.2, 125.6, 126.5, 130.5, 130.7, 131.3, 131.7, 132.6, 133.4, 134.9, 157.9, 164.2, 164.7 (four carbon signals overlapping or obscured). **HRMS** (ESI) 435.07028 ([M(^79^Br)+H]^+^), 437.06820 ([M(^81^Br)+H]^+^); calcd. for C_23_H_20_BrN_2_O_2_ 435.07027 ([M(^79^Br)+H]^+^), 437.06822 ([M(^81^Br)+H]^+^). The spectroscopic data were in agreement with those in the literature.

**2-(2-Hydroxyethyl)-6-(piperidin-1-yl)-1*H*-benzo[*de*]isoquinoline-1,3(2*H*)-dione (15).**

To a solution of **11** (844 mg, 3.00 mmol) in EtOH (10 mL) was added 2-aminoethanol (226 μL, 3.74 mmol). The reaction mixture was heated at reflux under Ar for 22 h and cooled to room temperature. The precipitate was filtered off and washed with ice-cold EtOH to give **15** as a yellow solid (900 mg, 92%). ***R*_F_** (EtOAc:hexane = 1:1) 0.42. **m.p.** 152-153 °C (lit. **m.p.** 136-138 °C). **IR** *ν*_max_/cm^-1^ 3461, 2935, 2852, 2811, 1690, 1646, 1582, 1515, 1452, 1424, 1377, 1348, 1229, 1178, 1132, 1075, 1050, 1029, 981, 896, 860, 839. **^1^H NMR** (400 MHz, CDCl_3_) *δ* 1.69-1.75 (m, 2H, CH_2_CH_2_C*H*_2_CH_2_CH_2_), 1.84-1.91 (m, 4H, CH_2_C*H*_2_CH_2_C*H*_2_CH_2_), 2.91 (t, 1H, *J* 5.6, CH_2_CH_2_O*H*), 3.21 (t, 4H, *J* 5.2, CH_2_NCH_2_), 3.96 (q, 2H, *J* 5.6, CH_2_C*H*_2_OH), 4.41 (t, 2H, *J* 5.6, C*H*_2_CH_2_OH), 7.11 (d, 1H, *J* 8.0, naphthalene-H), 7.62 (t, 1H, *J* 8.0, naphthalene-H), 8.32 (d, 1H, *J* 8.4, naphthalene-H), 8.42 (d, 1H, *J* 8.0, naphthalene-H), 8.50 (d, 1H, *J* 7.2, naphthalene-H). **^13^C NMR** (100 MHz, CDCl_3_) *δ* 24.4, 26.3, 42.7, 54.6, 62.0, 114.7, 115.4, 122.8, 125.3, 126.1, 130.0, 130.9, 131.3, 133.0, 157.6, 165.0, 165.4 (two carbon signals overlapping or obscured). **MS** (ESI) *m/z* 324.9 ([M+H]^+^, 5%), 346.8 ([M+Na]^+^, 12%), 670.7 ([2M+Na]^+^, 100%). **HRMS** (ESI) 325.15440 ([M+H]^+^); calcd. for C_19_H_21_N_2_O_3_ ([M+H]^+^) 325.15467. The spectroscopic data were in agreement with those in the literature.

**2-(2-Bromoethyl)-6-(piperidin-1-yl)-1*H*-benzo[*de*]isoquinoline-1,3(2*H*)-dione (16).**

To a solution of **15** (324 mg, 0.999 mmol) in THF (10 mL) were added pyridine (242 μL, 3.00 mmol) and PBr_3_ (285 μL, 3.00 mmol). The reaction mixture was heated at 50 °C for 16 h, poured into ice-cold H_2_O (10 mL) and extracted with CHCl_3_ (3 × 25 mL). The combined organic extracts were dried over MgSO_4_ and concentrated under reduced pressure. The residue was purified by flash column chromatography (silica gel, DCM) to give **16** as a yellow solid (232 mg, 60%). ***R*_F_** (DCM) 0.57. **m.p.** 185-186 °C (lit. **m.p.** 184.3-185.5 °C). **IR** *ν*_max_/cm^-1^ 2934, 2851, 2814, 1694, 1656, 1584, 1514, 1431, 1381, 1345, 1232, 1108. **^1^H NMR** (400 MHz, CDCl_3_) *δ* 1.69-1.76 (m, 2H, CH_2_CH_2_C*H*_2_CH_2_CH_2_), 1.85-1.92 (m, 4H, CH_2_C*H*_2_CH_2_C*H*_2_CH_2_), 3.23 (t, 4H, *J* 5.2, CH_2_NCH_2_), 3.65 (t, 2H, *J* 7.6, CH_2_C*H*_2_Br), 4.57 (t, 2H, *J* 7.6, C*H*_2_CH_2_Br), 7.16 (d, 1H, *J* 8.0, naphthalene-H), 7.66 (dd, 1H, *J* 8.4 & 7.2, naphthalene-H), 8.38 (dd, 1H, *J* 8.4 & 0.8, naphthalene-H), 8.47 (d, 1H, *J* 8.0, naphthalene-H), 8.54 (dd, 1H, *J* 7.2 & 1.2, naphthalene-H). **^13^C NMR** (100 MHz, CDCl_3_) *δ* 24.4, 26.3, 28.1, 41.1, 54.6, 114.8, 115.4, 122.8, 125.4, 126.3, 130.1, 131.1, 131.4, 133.1, 157.7, 163.8, 164.4 (two carbon signals overlapping or obscured). **HRMS** (ESI) 387.07035 ([M(^79^Br)+H]^+^), 389.06839 ([M(^81^Br)+H]^+^); calcd. for C_19_H_20_BrN_2_O_2_ 387.07027 ([M(^79^Br)+H]^+^), 389.06822 ([M(^81^Br)+H]^+^). The spectroscopic data were in agreement with those in the literature.

**2-(2-Azidoethyl)-6-(piperidin-1-yl)-1*H*-benzo[*de*]isoquinoline-1,3(2*H*)-dione (17).**

To a solution of **16** (387 mg, 0.999 mmol) in EtOH (10 mL) was added sodium azide (300 mg, 4.61 mmol). The reaction mixture was heated at reflux for 6 h and concentrated under reduced pressure. The residue was purified by flash column chromatography (silica gel, DCM:petroleum benzine = 1:1 ramping to DCM) to give **17** as an orange solid (279 mg, 80%). ***R*_F_** (DCM:hexane = 4:1) 0.28. **m.p.** 142-143 °C (lit. **m.p.** 138.0-139.6 °C). **IR** *ν*_max_/cm^-1^ 2936, 2851, 2811, 2099, 1694, 1654, 1583, 1515, 1452, 1382, 1356, 1278, 1232, 1177, 1137, 1079, 1047, 992, 918, 861. **^1^H NMR** (400 MHz, CDCl_3_) *δ* 1.69-1.75 (m, 2H, CH_2_CH_2_C*H*_2_CH_2_CH_2_), 1.85-1.91 (m, 4H, CH_2_C*H*_2_CH_2_C*H*_2_CH_2_), 3.22 (t, 4H, *J* 5.2, CH_2_NCH_2_), 3.64 (t, 2H, *J* 6.4, CH_2_C*H*_2_N_3_), 4.40 (t, 2H, *J* 6.4, C*H*_2_CH_2_N_3_), 7.14 (d, 1H, *J* 8.0, naphthalene-H), 7.64 (t, 1H, *J* 8.0, naphthalene-H), 8.36 (d, 1H, *J* 8.4, naphthalene-H), 8.46 (d, 1H, *J* 8.0, naphthalene-H), 8.54 (d, 1H, *J* 7.2, naphthalene-H). **^13^C NMR** (100 MHz, CDCl_3_) *δ* 24.4, 26.3, 38.7, 49.0, 54.6, 114.7, 115.4, 122.7, 125.4, 126.2, 130.1, 130.9, 131.3, 133.0, 157.6, 164.0, 164.6 (two carbon signals overlapping or obscured). **MS** (APCI) *m/z* 322.1 ([M-N_2_+H]^+^, 100%), 350.1 ([M+H]^+^, 20%). The spectroscopic data were in agreement with those in the literature.

**References**

1. Demko ZP and Sharpless KB (2001) An intramolecular [2 + 3] cycloaddition route to fused 5-heterosubstituted tetrazoles. Org Lett 3: 4091-4094.

2. Lau YH, Price JR, Todd MH and Rutledge PJ (2011) A click fluorophore sensor that can distinguish Cu^II^ and Hg^II^ *via* selective anion-induced demetallation. Chem Eur J 17: 2850-2858.

3. Yu M, Yu Q, Rutledge PJ and Todd MH (2013) A fluorescent “allosteric scorpionand” complex visualizes a biological recognition event. ChemBioChem 14: 224-229.

4. Yu M, Price JR, Jensen P, Lovitt CJ, Shelper T, et al. (2011) Copper, nickel, and zinc cyclam–amino acid and cyclam–peptide complexes may be synthesized with “click” chemistry and are noncytotoxic. Inorg Chem 50: 12823-12835.

5. Wang D, Zhang X, He C and Duan C (2010) Aminonaphthalimide-based imidazolium podands for turn-on fluorescence sensing of nucleoside polyphosphates. Org Biomol Chem 8: 2923-2925.

6. Peters AT and Bide MJ (1985) Amino derivatives of 1,8-naphthalic anhydride and derived dyes for synthetic-polymer fibres. Dyes Pigm 6: 349-375.

7. Xu H, Zeng X and Dai H (2011) A new fluorescent chemosensor for metal ions based upon 1,8-naphthalimide and 8-hydroxyquinoline. Chin J Chem 29: 2165-2168.

8. Dai H and Xu H (2012) Selective and sensitive fluorescent chemosensors for Cu^2+^ ion based upon bis-1,8-naphthalimide dyads. Chin J Chem 30: 267-272.

9. Ding G, Xu Z, Zhong G, Jing S, Li F, et al. (2008) Synthesis, photophysical and electroluminescent properties of novel naphthalimide derivatives containing an electron-transporting unit. Res Chem Intermed 34: 299-308.

10. Lukas AS, Bushard PJ and Wasielewski MR (2002) Electron transfer involving nonbonded superexchange interactions in rigid donor−acceptor arrays. J Phys Chem A 106: 2074-2082.

11. Dai H, Yan Y, Guo Y, Fan L, Che Z, et al. (2012) A selective and sensitive “turn-on” fluorescent chemosensor for recognition of Hg^2+^ ions in water. Chem Eur J 18: 11188-11191.

12. Zhang G-F, Aldred MP, Gong W-L, Li C and Zhu M-Q (2012) Utilising tetraphenylethene as a dual activator for intramolecular charge transfer and aggregation induced emission. Chem Commun 48: 7711-7713.

13. Tagg T, McAdam CJ, Robinson BH and Simpson J (2008) *N*-(2-Bromoethyl)-4-piperidino-1,8-naphthalimide and *N*-(3-bromopropyl)-4-piperidino-1,8-naphthalimide. Acta Crystallogr C 64: o388-o391.
